# Night shift work exposure shapes neurobehavioral and cardiometabolic profiles in female healthcare workers: a cross-sectional study

**DOI:** 10.3389/fpubh.2026.1860519

**Published:** 2026-06-10

**Authors:** Silvia Vivarelli, Tania Formica, Francesca Simona Fiorino, Manuela Pollicino, Saveria Savasta, Caterina Oliveri, Laura Trifilò, Federica Giambò, Concettina Fenga

**Affiliations:** 1 Department of Biomedical and Dental Sciences, Morphological and Functional Imaging, Section of Occupational Medicine, University of Messina, Messina, Italy; 2 Vita-Salute San Raffaele University, Milan, Italy

**Keywords:** cardiometabolic risk, cumulative exposure, female healthcare workers, insulin resistance, night shift work, precision prevention, sleep quality

## Abstract

**Background:**

Night shift work has been associated with adverse neurobehavioral and cardiometabolic outcomes, but the role of cumulative and long-term exposure, particularly among female healthcare workers, remains incompletely characterized. This study adopted a multidimensional approach to examine associations between night shift exposure and integrated health profiles.

**Methods:**

This cross-sectional study included 243 female healthcare workers (96 day-shift, 147 night-shift). Night shift exposure was assessed using multiple metrics: status, cumulative lifetime shifts (<500 vs. ≥ 500), and duration (1–10 vs. 11–35 years). Sociodemographic, reproductive, lifestyle, and occupational data were collected. Neurobehavioral outcomes were evaluated using validated questionnaires (sleep quality, psychological well-being, work ability), while cardiometabolic assessment included BMI, WHtR, insulin resistance indices (METS-IR, TyG, WyG), and cardiovascular risk index (IRCV). Analyses comprised stratification, correlation, and multivariable regression adjusted for age, smoking, and menopausal status.

**Results:**

Higher cumulative exposure was associated with lower physical activity (*p* = 0.012), poorer sleep (*p* = 0.019), reduced work ability (*p* = 0.023), and early insulin resistance (*p* = 0.043). Long-term exposure was linked to poorer psychological well-being (*p* = 0.009), higher adiposity (*p* = 0.034; *p* = 0.028), and increased IRCV (*p* = 0.006). In adjusted models, sleep quality was consistently associated with all exposure metrics, while metabolic outcomes were more strongly linked to duration of exposure and individual factors. Correlation analyses revealed clustering of sleep, neurobehavioral, and cardiometabolic variables, with strength of the associations increasing with exposure level.

**Conclusion:**

Night shift work, particularly with cumulative and long-term exposure, is associated with sleep disruption, reduced well-being, and early cardiometabolic alterations in female healthcare workers. Sleep impairment emerges as a robust exposure-related marker, while metabolic risk reflects combined occupational and individual susceptibility. These findings support life-course exposure assessment and suggests the use of early subclinical indicators for implementation of precision prevention, as well as early intervention strategies. Future research may further benefit from a life-course exposome framework integrating environmental, occupational, and biological exposures.

## 1 Introduction

Shift work is essential to modern healthcare systems, ensuring continuity of care across 24-h services, but it also represents a relevant occupational exposure with potential implications for worker health (1). Among different shift patterns, night work is particularly disruptive due to its misalignment with endogenous circadian rhythms, making it a priority area for occupational and public health research (2). Healthcare professionals frequently experience chronic misalignment between circadian rhythms and work-rest schedules, which has been associated with a range of adverse short- and long-term health outcomes (3).

Acute effects of night shift work include sleep disruption, excessive daytime sleepiness, impaired alertness, mood alterations, and reduced work ability, which may compromise both worker well-being and patient safety (4–6). Beyond these immediate effects, evidence suggests that persistent exposure to night work may contribute to longer-term health alterations (7). Chronic night work is associated with obesity, insulin resistance, type 2 diabetes, dyslipidemia, hypertension, cardiovascular disease, and metabolic syndrome (8, 9). Beneath mechanisms include: circadian disruption, hormonal imbalance, impaired glucose and lipid metabolism, low-grade inflammation, autonomic dysfunction, and nocturnal light-induced melatonin suppression (10). In addition, night shift work has been investigated in relation to an increased risk of hormone-sensitive cancers, particularly breast cancer in women, although evidence remains heterogeneous across populations and exposure definitions (11, 12).

Women may represent a potentially vulnerable subgroup due to complex interactions between occupational exposures, circadian biology, and reproductive factors, including parity, breastfeeding history, menopausal status, and hormonal therapy use (13, 14). In addition, female healthcare workers are often exposed to additional psychosocial stressors such as caregiving responsibilities, work-life imbalance, and emotional labor, which may further influence health outcomes and coping capability in shift work conditions (15–17). Lifestyle-related behaviors also play a relevant role, as night shift workers have been reported to more frequently exhibit lower diet quality, irregular meal timing, reduced physical activity, increased sedentary behavior, and higher prevalence of smoking and alcohol consumption (18).

Despite these concerns, most existing studies have focused on single health outcomes or have been conducted in heterogeneous or predominantly male populations, limiting the understanding of sex-specific and multidimensional health profiles (4, 19, 20). Moreover, there is still limited integration of occupational exposure data with reproductive, lifestyle, metabolic, and clinical domains, which restricts the ability to capture early subclinical alterations and cumulative risk patterns in exposed workers (4). Consequently, there is a growing need for integrative approaches that consider cumulative and multidimensional exposure-outcome relationships rather than isolated associations (21). Such approaches may improve understanding of early health changes associated with occupational night work and support more comprehensive preventive strategies in working populations.

Accordingly, this study examined associations between night shift work exposure and a multidimensional set of neurobehavioral, metabolic, and cardiovascular outcomes in female healthcare workers. Multiple exposure indicators were used to capture cumulative occupational history and to explore exposure-response patterns across increasing levels of night shift work. Regression models were adjusted for key potential confounders, including age, smoking habits, and menopausal status. The aim was not to infer causality, but to provide an integrated approach of exposure-related health profiles, to support hypothesis generation for future longitudinal and mechanistic studies in occupational health, as well as guide tailored prevention in healthcare settings.

## 2 Methods

### 2.1 Study design and population

This is an observational cross-sectional study conducted within the occupational health surveillance program of a university hospital located in south Italy and included female healthcare workers employed in clinical and administrative roles. Eligible participants were aged ≥18 years and had been working under their current shift schedule for at least 6 months. Exclusion criteria included diabetes mellitus, breast cancer, or other chronic conditions affecting metabolic, cardiovascular, or psychological outcomes.

Night shift work was defined as work including at least 3 h of a daily shift (or a defined proportion of annual working time) performed during a reference period of 7 h that includes the interval between midnight and 5 a.m., in accordance with Directive 2003/88/EC (Articles 2.4 and 2.5) and institutional shift organization practices (14). Duration and history of exposure to night shift work were recorded (22).

Of 264 consenting workers, 243 met inclusion criteria and were classified as day shift workers (DSW) or night shift workers (NSW). All participants provided written informed consent, and the study was approved by the local Ethical Committee of the University Hospital “G. Martino,” Messina (C. E. L. Prot. No. 28–24, dated 03/04/2024), and by Resolution of the Extraordinary Commissioner (Del. No. 830, dated 23/05/2024), in accordance with Helsinki Declaration and institutional occupational health surveillance standards.

### 2.2 Occupational health surveillance protocol

Participants underwent standardized clinical and occupational assessments conducted by occupational physicians and psychologists, between January 2024 and December 2025. Medical and reproductive history included age at menarche, parity, breastfeeding, menopausal status and age, use of hormonal therapies or oral contraceptives. Anthropometrics (height, weight, waist circumference) and blood pressure were measured using standard procedures, and lifestyle factors were collected via structured interviews, including smoking, alcohol use, physical activity, and self-reported sleep quality. All measurements and questionnaires were administered using the same standardized protocols for DSW and NSW, ensuring comparability between groups. Given the cross-sectional nature of the study and potential age-related confounding, sensitivity analyses were conducted in participants aged ≥30 years.

Night shift exposure was categorized by recency (recent: <6 vs. ≥ 6 shifts/month in the past 6 months), cumulative (<500 vs. ≥ 500 lifetime night shifts), duration (1–10 vs. 11–35 years), and intensity (low: <4 shifts/month; medium: ≥4 shifts/month for 1–9 years; high: ≥4 shifts/month for ≥10 years). These categorizations were based on distributional properties of the cohort and previously reported exposure metrics in occupational epidemiology literature (23–25). While recency and intensity were used for descriptive and stratified analyses, cumulative exposure and lifetime exposure duration were selected as primary analytical variables as indicators of long-term circadian disruption and occupational burden.

### 2.3 Neurobehavioral assessments

Neurobehavioral and lifestyle domains were assessed using validated self-administered questionnaires, including adherence to the Mediterranean diet (Medi-Lite), physical activity (International Physical Activity Questionnaire, IPAQ), sleep quality (Pittsburgh Sleep Quality Index, PSQI), psychological well-being (WHO-5 Well-Being Index), and work ability (Work Ability Index, WAI) (26–30). All instruments were scored according to validated methods, and established cut-offs were applied for categorical and binary analyses, as detailed in Supplementary Table S1.

### 2.4 Blood sampling and laboratory testing

Venous blood samples were collected after an overnight fast of ≥8 h under standardized conditions. Laboratory analyses included biochemical measurements relevant to metabolic and cardiovascular risk assessment, including fasting glucose and lipid profile. Complete blood count parameters, blood urea nitrogen, creatinine, liver enzymes, were also collected as part of routine occupational health surveillance, but were not included in the present analyses. All analyses were performed using standard procedures in certified clinical laboratories (31).

### 2.5 Composite health indices

Anthropometric, metabolic, and cardiovascular risk were evaluated using established composite indices derived from anthropometric and fasting biochemical measurements. These included body mass index (BMI), waist-to-height ratio (WHtR), insulin resistance indices (METS-IR, TyG, WyG), lipid ratios (LDL/HDL and total cholesterol/HDL), and a composite cardiovascular risk index (IRCV) (32–39). All indices were analyzed as continuous variables and, where appropriate, categorized or dichotomized using validated cut-offs, as detailed in Supplementary Table S2.

### 2.6 Statistical analyses

Statistical analyses were performed using IBM SPSS v23 and GraphPad Prism v9. Data distribution was assessed using the Kolmogorov–Smirnov test. Continuous variables are presented as mean ± standard deviation or median (range), and categorical variables as frequencies and percentages. Between-group comparisons were conducted using the Mann–Whitney U test for continuous variables and Chi-square tests for categorical variables. Associations among exposure variables, clinical outcomes, and questionnaire scores were assessed using Spearman’s rank correlation coefficients (*ρ*). *p*-values were adjusted for multiple testing using false discovery rate (FDR) correction. Mantel tests based on distance matrices were used to evaluate similarity patterns across correlation structures, and results were visualized using ChiPlot (40). Potential confounding in the associations between night shift exposure and health outcomes was addressed using multiple linear regression models (ordinary least squares), adjusting for age, smoking and menopausal status. For each outcome, three separate models were constructed using different exposure metrics (NSW status, cumulative exposure, and years of exposure). All models were adjusted for age, smoking status, and menopausal status. Regression coefficients (*β*), *p*-values, and coefficients of determination (R^2^) were reported. Analyses were performed on complete-case datasets; participants with missing values in exposure or outcome variables were excluded from the respective models. Statistical significance was set at *p* < 0.05.

## 3 Results

### 3.1 Study population characteristics show differences by work schedule

The study included 243 female healthcare workers: 96 day shift workers (DSW, 39.5%) and 147 night shift workers (NSW, 60.5%). NSW were older (45.5 ± 11.1 vs. 40.1 ± 13.0 years, *p* < 0.001) and had slightly higher waist circumference (85.7 ± 9.9 vs. 83.3 ± 10.0 cm, *p* = 0.025), whereas weight and height were comparable between groups (Supplementary Tables S3, S4).

Differences in reproductive history were observed, with NSW showing higher prevalence of ever pregnancy (66.0% vs. 38.5%, *p* < 0.001), having children (66.0% vs. 38.5%, *p* < 0.001), breastfeeding history (55.1% vs. 35.4%, *p* = 0.003), and menopause (34.7% vs. 19.8%, *p* = 0.012). Use of hormonal contraceptives also differed between groups (*p* = 0.013), whereas hormone replacement therapy did not (Supplementary Table S5).

Lifestyle and occupational characteristics also differed. Smoking prevalence was higher among NSW (31.3% vs. 16.7%, *p* = 0.029), and poor sleep quality was more frequently reported (46.3% vs. 33.3%, *p* = 0.003), while alcohol consumption and physical activity levels were similar between groups. Occupational roles differed significantly (*p* < 0.001), with nurses being more prevalent among NSW (62.8%) and healthcare executives among DSW (41.0%; Supplementary Table S6).

Among NSW, heterogeneity in exposure patterns was observed: most participants reported short-term exposure (1–10 years; 57.1%), cumulative exposure <500 night shifts (64.6%), and 54.5% reported working ≥4 night shifts/month for ≥10 years (Supplementary Table S7).

In a sensitivity analysis restricted to participants aged ≥30 years (*N* = 197), no significant differences were observed in age, weight, height, or waist circumference between groups. However, differences persisted for reproductive history (ever pregnancy: 73.5% vs. 56.9%, *p* = 0.019), smoking (31.1% vs. 15.4%, *p* = 0.044), poor sleep quality (17.4% vs. 4.6%, *p* = 0.019), and occupational roles (Supplementary Tables S8–S12).

### 3.2 Neurobehavioral outcomes associate with night shift exposure

**Figure 1:**
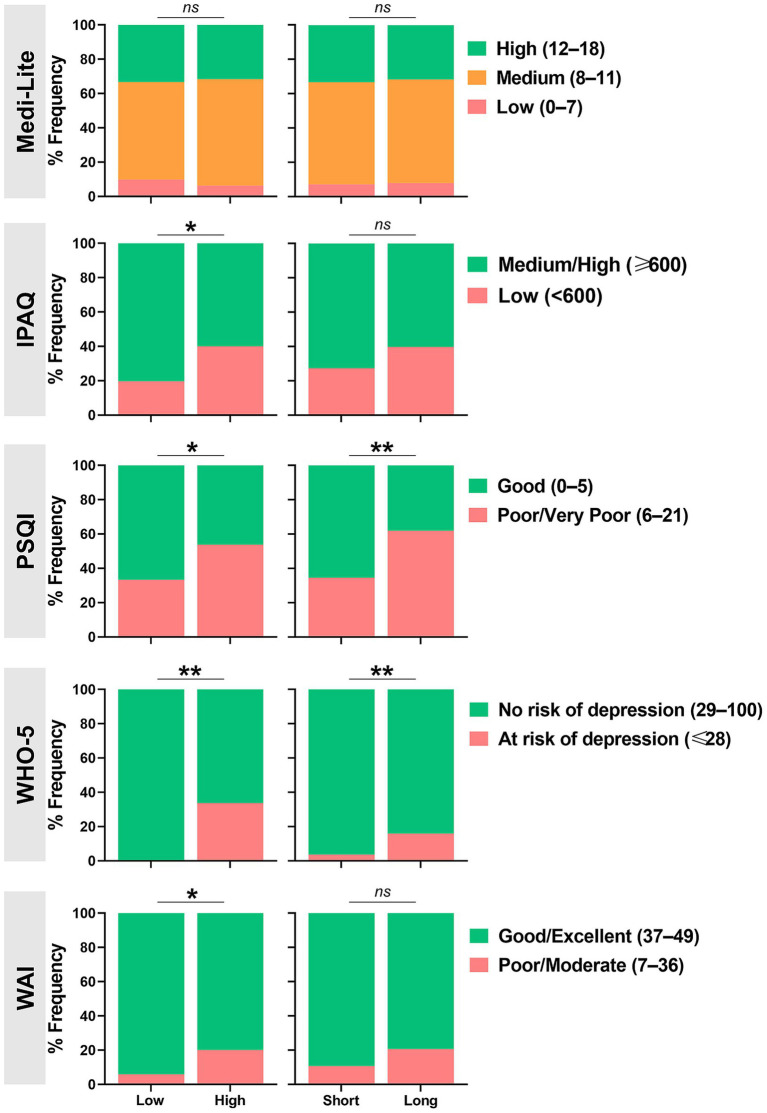
Neurobehavioral outcomes in female healthcare workers by night shift exposure. Bar plots show percentage distributions of night shift workers (NSW) stratified by low vs. high cumulative exposure (left) and short vs. long duration (right) for: Medi-Lite (Mediterranean diet adherence), IPAQ (physical activity), PSQI (sleep quality), WHO-5 (psychological well-being), and WAI (work ability). Significance: *p < 0.01, *p* < 0.05; ns, not significant.

Adherence to the Mediterranean diet (Medi-Lite) was comparable between DSW and NSW, with medium adherence most frequently reported in both groups (DSW 67.7%, NSW 59.9%). Within NSW, neither cumulative exposure (<500 vs. ≥ 500 lifetime shifts) nor duration (1–10 vs. 11–35 years) showed significant associations with dietary adherence (Supplementary Table S13; Figure 1).

Physical activity (IPAQ) showed broadly similar distributions between groups, with medium activity predominating (DSW 37.5%, NSW 43.5%). However, low-to-moderate activity levels were more frequent among NSW (75.8% vs. 61.5%, *p* = 0.039), and higher cumulative exposure was associated with a greater prevalence of low physical activity (40.0% vs. 19.6%, *p* = 0.012; Supplementary Table S14; Figure 1).

Sleep quality (PSQI) was worse in NSW, with a lower percentage reporting good sleep (53.7% vs. 66.7%) and a higher percentage reporting poor/very poor sleep (46.3% vs. 33.3%, *p* = 0.045). Within NSW, both higher cumulative exposure and longer duration were associated with progressively poorer sleep quality (*p* = 0.019 and *p* = 0.001, respectively; Supplementary Table S15; Figure 1).

Although no statistically significant between-group differences were observed in psychological well-being (WHO-5; good/very good: 80.2% in DSW vs. 70.7% in NSW), a higher percentage of poor/very poor well-being was observed among NSW (19.8% vs. 29.3%). Within NSW, higher cumulative exposure and longer duration were associated with increased prevalence of very poor/poor well-being and higher risk of depressive symptomatology (WHO-5 ≤ 28: cumulative 13.7% vs. 0%, *p* = 0.006; duration 15.9% vs. 3.6%, *p* = 0.009; Supplementary Table S16; Figure 1).

Work ability (WAI) remained generally high in both groups (good/excellent: DSW 89.6%, NSW 85.0%), although poor/moderate scores were slightly more frequent in NSW (15.0% vs. 10.4%). Among NSW, higher cumulative exposure was associated with poorer work ability (20.0% vs. 5.9%, *p* = 0.023), whereas longer exposure showed a non-significant trend in the same direction (20.6% vs. 10.7%, *p* = 0.095; Supplementary Table S17; Figure 1). Combined mental and physical effort was more frequently reported among NSW, whereas mental-only effort predominated in DSW (*p* < 0.05).

Overall, these results suggest heterogeneous associations between night shift exposure and neurobehavioral outcomes, with more consistent patterns observed for sleep quality and selected psychosocial and functional outcomes, particularly when exposure is considered in terms of cumulative history and duration.

### 3.3 Prolonged night shift work is linked to early anthropometric, cardiovascular, and metabolic changes

Body mass index (BMI) did not differ significantly between DSW and NSW, with most participants in the normal weight range and a low prevalence of obesity (median BMI: 22 vs. 23 kg/m^2^; BMI ≥ 25: 29.2% vs. 33.3%, *p* = 0.495). Central adiposity (WHtR >0.5) showed a non-significant trend toward higher prevalence in NSW (61.2% vs. 50.0%, *p* = 0.084; Supplementary Table S18). Similar patterns were observed in the age-restricted sensitivity analysis (≥30 years; Supplementary Table S19).

**Figure 2:**
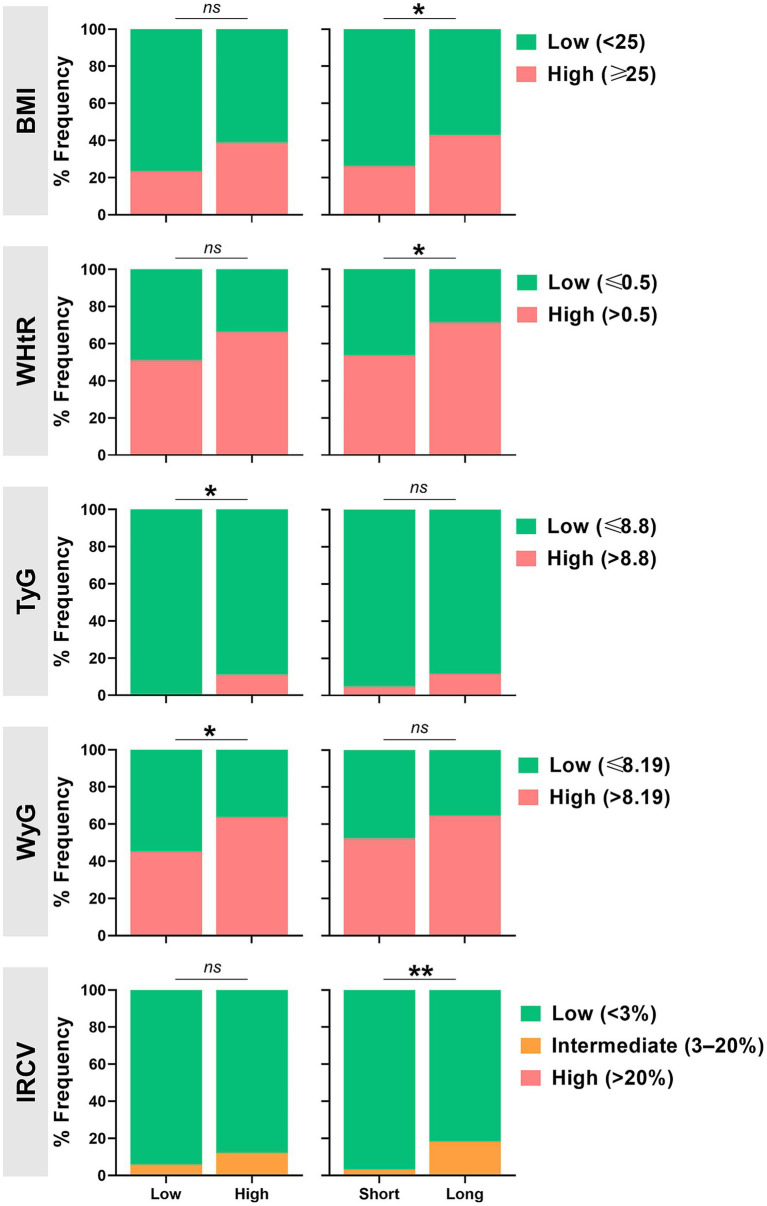
Anthropometric, metabolic, and cardiovascular indices in female healthcare workers by night shift exposure. Bar plots show percentage distributions of NSW stratified by low vs. high cumulative exposure (left) and short vs. long duration (right) for: BMI (body mass index), WHtR (waist-to-height ratio), TyG (triglyceride–glucose index), WyG (waist–triglyceride–glucose index), and IRCV (cardiovascular risk index). Significance: ^*^p < 0.01, *p* < 0.05; ns, not significant.

Within NSW, higher cumulative exposure showed a non-significant tendency toward higher prevalence of overweight/obesity and central adiposity (BMI ≥ 25: 38.9% vs. 23.5%, *p* = 0.060; WHtR >0.5: 66.3% vs. 51.0%, *p* = 0.070), whereas longer duration of exposure was significantly associated with both outcomes (BMI ≥ 25: 42.9% vs. 26.2%, *p* = 0.034; WHtR >0.5: 71.4% vs. 53.6%, *p* = 0.028; Supplementary Tables S20, S21; Figure 2).

Metabolic indices (METS-IR, TyG, WyG) and lipid ratios (TC/HDL, LDL/HDL) were overall comparable between DSW and NSW, suggesting no clear differences in metabolic profiles according to shift status alone (Supplementary Tables S18, S19). Within NSW, higher cumulative exposure was associated with a higher prevalence of selected adverse metabolic thresholds, including TyG > 8.8 (11.1% vs. 0%, *p* = 0.043) and WyG > 8.19 (63.8% vs. 45.1%, *p* = 0.029; Supplementary Tables S20, S21; Figure 2).

The Cardiovascular Risk Index (IRCV) remained generally low in both groups (median 0.58 in DSW vs. 0.65 in NSW), with most participants classified at low risk (<3%) and no participants in the high-risk category. Within NSW, higher cumulative exposure showed no statistically significant difference in IRCV (0.32 vs. 0.80, *p* = 0.304), whereas longer duration of exposure was associated with a more unfavorable distribution, with fewer low-risk and more intermediate-risk participants (low risk: 81.7% vs. 96.9%; intermediate risk: 18.3% vs. 3.1%, *p* = 0.006; Supplementary Tables S20, S21; Figure 2).

Overall, these findings suggest that anthropometric and cardiometabolic profiles are more consistently related to longer duration of night shift exposure than to shift status alone or cumulative exposure, although effect sizes were generally modest and several associations did not reach statistical significance.

### 3.4 Exposure-dependent associations of night shift work with sleep quality, work ability, and cardiometabolic indicators in confounder-adjusted regression models

In fully adjusted multiple linear regression models, different operationalizations of night shift exposure (i.e., night shift status, cumulative exposure, and years of exposure) were evaluated primarily to assess the robustness of exposure-outcome relationships after adjustment for key potential confounders, including age, smoking status, and menopausal status. Across models, exposure metrics showed heterogeneous associations with outcomes, with different degrees of sensitivity to adjustment variables (Tables 1–3).

**Table 1:** Multivariable linear regression models for associations between night shift status and neurobehavioral, cardiometabolic outcomes.

Outcome	NSW β (95% CI)	Age β (95% CI)	Smoking β (95% CI)	Menopause β (95% CI)	R^2^
PSQI	0.8857* (0.1617 to 1.610)	0.0393 (−0.00387 to 0.08248)	−0.0133 (−0.8063 to 0.7798)	1.737** (0.5921 to 2.881)	0.2020
WAI	0.1426 (−1.019 to 1.304)	−0.1023** (−0.1716 to −0.03307)	−0.1768 (−1.449 to 1.095)	−2.047* (−3.883 to −0.2112)	0.1824
IRCV	−0.0104 (−0.3653 to 0.3444)	0.0561*** (0.03482 to 0.07742)	0.8408*** (0.4696 to 1.212)	0.7337** (0.2491 to 1.218)	0.4806
METS-IR	0.3775 (−1.921 to 2.676)	0.1943** (0.05527 to 0.3333)	−0.8334 (−3.251 to 1.584)	−0.5167 (−3.651 to 2.617)	0.0795
TyG	−0.0398 (−0.1936 to 0.1139)	0.0155** (0.00619 to 0.02475)	−0.0213 (−0.1841 to 0.1416)	0.0502 (−0.1606 to 0.2609)	0.1500
WyG	0.0169 (−0.0267 to 0.0605)	0.00418** (0.00159 to 0.00677)	−0.0330 (−0.0808 to 0.0149)	0.0324 (−0.0363 to 0.1011)	0.1475

β coefficients are adjusted for age, smoking status, and menopausal status. R^2^ indicates model explanatory power. PSQI, Pittsburgh Sleep Quality Index; WAI, Work Ability Index; IRCV, Cardiovascular Risk Index; METS-IR, metabolic score for insulin resistance; TyG, triglyceride-glucose index; WyG, weight-adjusted glucose index. *p < 0.05, **p < 0.01, ***p < 0.001.

**Table 2:** Multivariable linear regression models for associations between cumulative night shift exposure and neurobehavioral, cardiometabolic outcomes.

Outcome	CUM β (95% CI)	Age β (95% CI)	Smoking β (95% CI)	Menopause β (95% CI)	R^2^
PSQI	0.00114** (0.0003529 to 0.001928)	0.00542 (−0.06404 to 0.07488)	−0.1997 (−1.192 to 0.7926)	1.744* (0.3217 to 3.166)	0.2255
WAI	−0.00131* (−0.002603 to −2.114e-005)	−0.0391 (−0.1525 to 0.07430)	−0.5015 (−2.126 to 1.123)	−2.036 (−4.366 to 0.2945)	0.1690
IRCV	7.53e-05 (−0.0002567 to 0.0004073)	0.0629*** (0.03172 to 0.09414)	0.8734*** (0.4142 to 1.333)	0.6581* (0.05259 to 1.264)	0.4573
METS-IR	0.00150 (−0.0005356 to 0.003527)	0.1175 (−0.07242 to 0.3074)	−1.596 (−4.377 to 1.185)	−1.183 (−4.879 to 2.512)	0.0651
TyG	0.000101 (−2.919e-005 to 0.0002311)	0.00768 (−0.00449 to 0.01985)	−0.0794 (−0.2576 to 0.09879)	0.0622 (−0.1745 to 0.2990)	0.1191
WyG	4.01e-05 (−7.47e-006 to 8.77e-005)	0.00197 (−0.00221 to 0.00615)	−0.0538 (−0.1141 to 0.00643)	0.0100 (−0.07595 to 0.09586)	0.1018

β coefficients are adjusted for age, smoking status, and menopausal status. R^2^ indicates model explanatory power. PSQI, Pittsburgh Sleep Quality Index; WAI, Work Ability Index; IRCV, Cardiovascular Risk Index; METS-IR, metabolic score for insulin resistance; TyG, triglyceride-glucose index; WyG, weight-adjusted glucose index. *p < 0.05, **p < 0.01, ***p < 0.001.

**Table 3:** Multivariable linear regression models for associations between duration of night shift exposure and neurobehavioral, cardiometabolic outcomes.

Outcome	YEARS β (95% CI)	Age β (95% CI)	Smoking β (95% CI)	Menopause β (95% CI)	R^2^
PSQI	0.0713* (0.00949 to 0.1331)	0.0184 (−0.04992 to 0.08671)	−0.1371 (−1.134 to 0.8597)	1.610* (0.1768 to 3.043)	0.2115
WAI	−0.0855 (−0.1865 to 0.01564)	−0.0520 (−0.1638 to 0.05977)	−0.5591 (−2.190 to 1.072)	−1.880 (−4.224 to 0.4644)	0.1618
IRCV	−0.2706 (−0.5829 to 0.04177)	−0.4424* (−0.7877 to −0.09707)	6.842** (1.803 to 11.88)	5.080 (−2.163 to 12.32)	0.1672
METS-IR	0.3477* (0.08019 to 0.6152)	0.4838** (0.1880 to 0.7796)	−4.927* (−9.242 to −0.6110)	−5.982 (−12.19 to 0.2223)	0.2319
TyG	0.0686* (0.00860 to 0.1286)	0.1068** (0.04053 to 0.1731)	−1.021* (−1.988 to −0.05318)	−1.198 (−2.589 to 0.1929)	0.2165
WyG	0.00141 (−0.01424 to 0.01707)	0.00312 (−0.01419 to 0.02042)	−0.2178 (−0.4704 to 0.03468)	0.0689 (−0.2941 to 0.4319)	0.0341

β coefficients are adjusted for age, smoking status, and menopausal status. R^2^ indicates model explanatory power. PSQI, Pittsburgh Sleep Quality Index; WAI, Work Ability Index; IRCV, Cardiovascular Risk Index; METS-IR, metabolic score for insulin resistance; TyG, triglyceride-glucose index; WyG, weight-adjusted glucose index. *p < 0.05, **p < 0.01, ***p < 0.001.

For sleep quality (PSQI), all exposure definitions remained significantly associated with worse scores, including night shift status (*β* = 0.886, *p* = 0.0167), cumulative exposure (*β* = 0.00114, *p* = 0.0048), and years of exposure (*β* = 0.0713, *p* = 0.0241), indicating a robust association that persisted after adjustment for confounders. Work ability (WAI) showed weaker and less consistent associations, with a modest inverse association observed only for cumulative exposure (*β* = −0.00131, *p* = 0.0464), while night shift status and duration of exposure were not statistically significant after adjustment.

**Figure 3:**
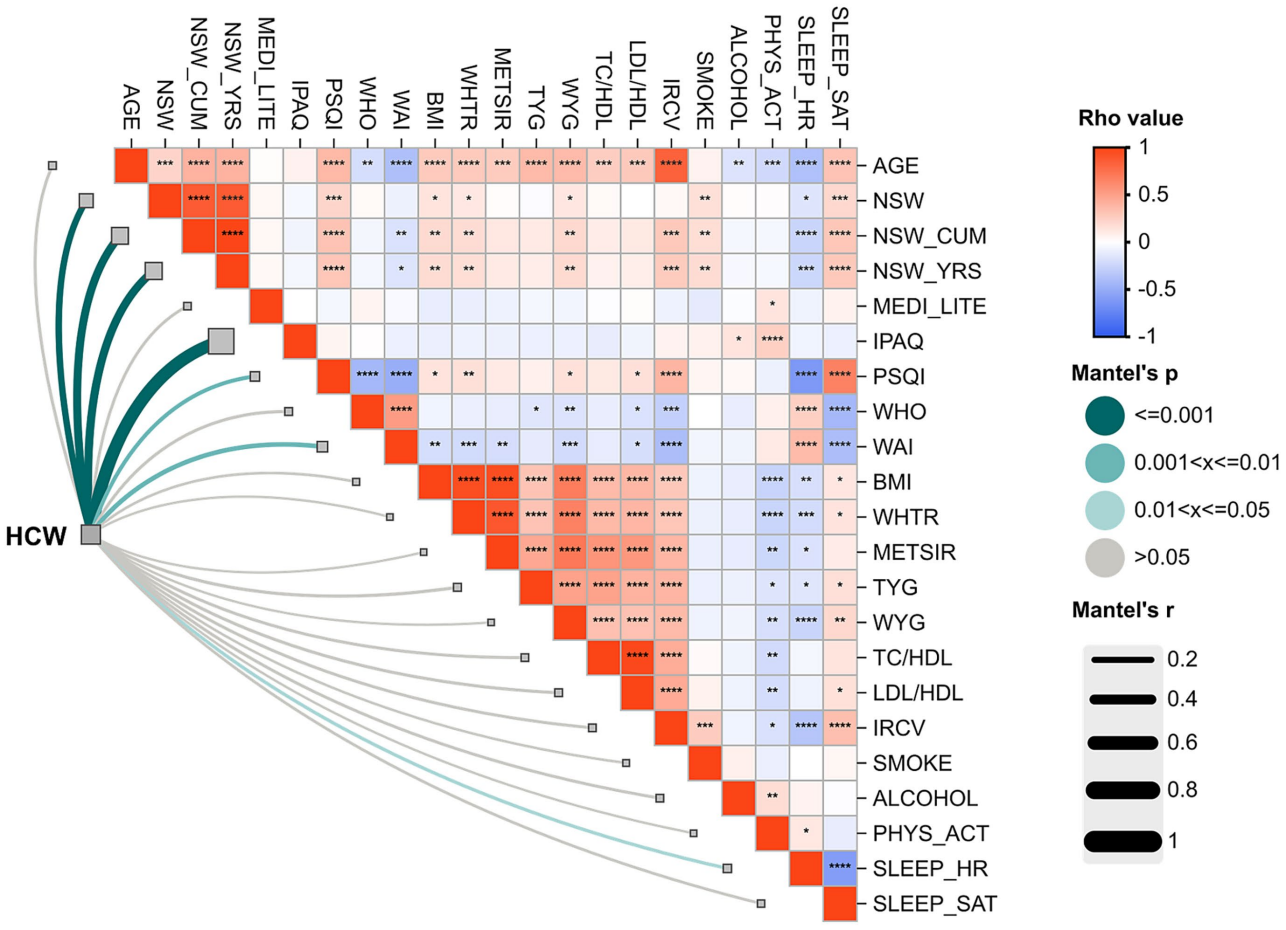
Correlation matrix of sociodemographic, lifestyle, neurobehavioral, and cardiometabolic variables in female healthcare workers. Spearman correlations are shown (red = positive, blue = negative), with Mantel test results overlaid (lines: width = *r* statistic; green shading = *p*-value). Only statistically significant correlations are displayed (*p < 0.05, **p < 0.01, ***p < 0.001, ****p < 0.0001). Variables: AGE (years), NSW (ever night shift), NSW_CUM (cumulative lifetime shifts), NSW_YRS (years of night shift), MEDI_LITE, IPAQ, PSQI, WHO-5, WAI, BMI, WHtR, METS-IR, TyG, WyG, TC/HDL, LDL/HDL, IRCV, SMOKE, ALCOHOL, PHYS_ACT (≥1/week), SLEEP_HR (hours/night), SLEEP_SAT (sleep satisfaction).

For cardiometabolic indices, results were more strongly influenced by adjustment variables. Night shift status showed no significant associations with IRCV, TyG, METS-IR, or WyG, suggesting limited independent contribution of binary exposure classification. In contrast, years of exposure retained significant associations with TyG (*β* = 0.0686, *p* = 0.0253) and METS-IR (*β* = 0.348, *p* = 0.0112), even after adjustment, while cumulative exposure showed no consistent independent effects, with only borderline associations for IRCV (*p* = 0.0978; Figure 3).

Across models, age, smoking, and menopausal status showed variable and, in some cases, strong associations with outcomes (particularly IRCV and METS-IR), supporting their role as relevant confounders in the exposure–outcome relationships. Overall, sleep-related outcomes are robust across adjustment, whereas cardiometabolic outcomes appear more sensitive to exposure definition and confounding structure, particularly with respect to exposure duration.

### 3.5 Night shift exposure is associated with reorganization of neurobehavioral and cardiometabolic risk clustering

**Figure 4:**
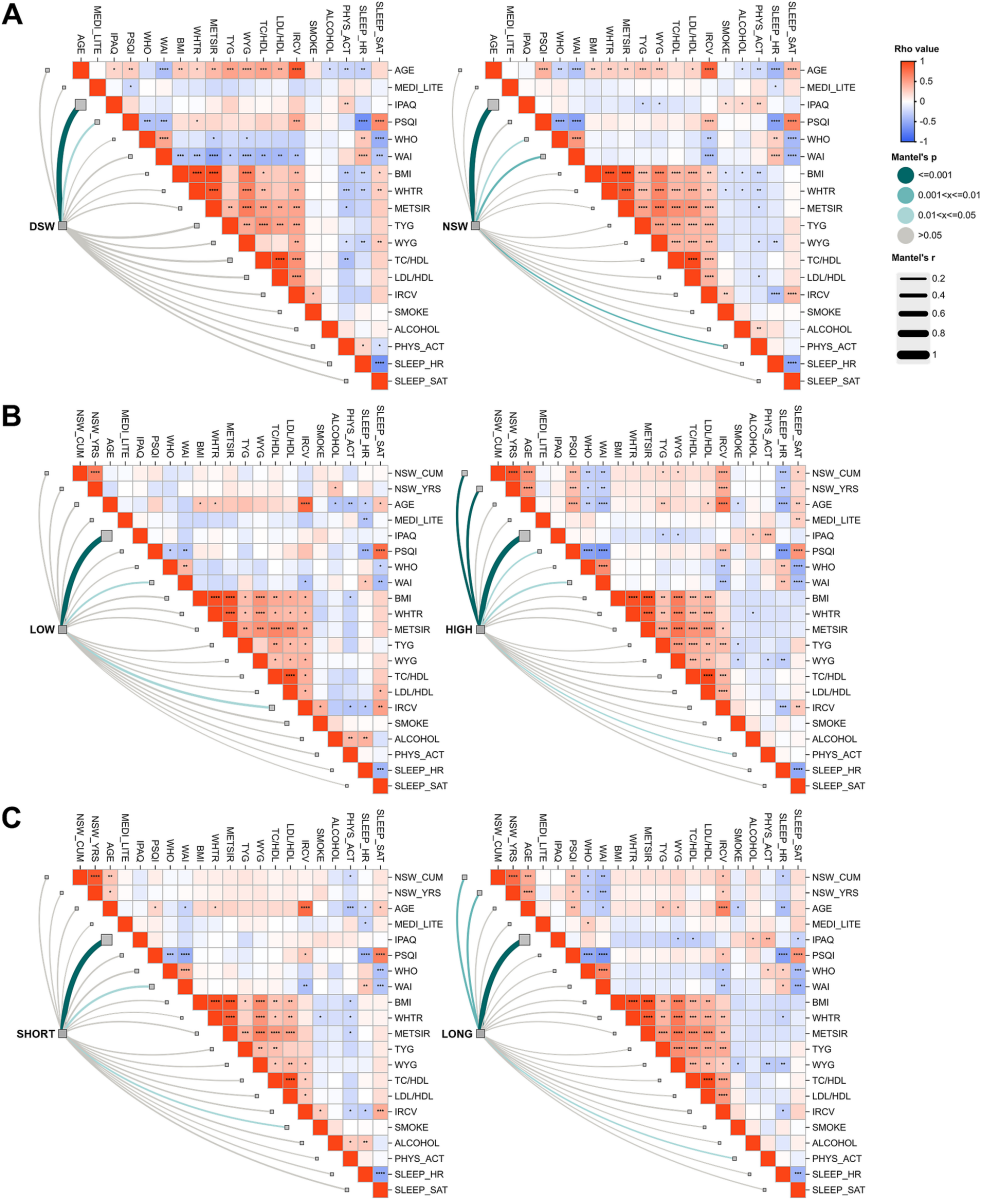
Correlation matrices stratified by night shift exposure in female healthcare workers. Spearman correlations and Mantel test results are shown for: **(A)** Day shift workers (DSW) vs. night shift workers (NSW), **(B)** NSW with low vs. high cumulative exposure, **(C)** NSW with short vs. long duration. Red = positive correlations (*ρ* 0–1); blue = negative correlations (ρ 0 to −1). Only statistically significant correlations are shown (*p < 0.05, **p < 0.01, ***p < 0.001, ****p < 0.0001). Mantel tests: lines width = r statistic; green shading = p-value. Variables: AGE (years), NSW_CUM (cumulative lifetime shifts), NSW_YRS (years of night shift), MEDI_LITE, IPAQ, PSQI, WHO-5, WAI, BMI, WHtR, METS-IR, TyG, WyG, TC/HDL, LDL/HDL, IRCV, SMOKE, ALCOHOL, PHYS_ACT (≥1/week), SLEEP_HR (hours/night), SLEEP_SAT (sleep satisfaction).

Stratified correlation and strength analyses revealed distinct patterns of health-related clustering according to work schedule and intensity of night shift exposure. For instance, in DSW, age emerged as the primary organizing factor of the health network, showing strong associations with cardiovascular risk (IRCV; *ρ* = 0.89, *p* < 0.001) and adiposity- and metabolic-related indices (BMI, WHtR, METS-IR, TyG, WyG, TC/HDL, LDL/HDL; ρ = 0.25–0.49, *p* < 0.01). Age was also linked to poorer sleep quality (PSQI, sleep duration) and lower work ability (WAI; *ρ* = −0.39, *p* < 0.001). Within this group, sleep quality was closely interconnected with sleep duration, psychological well-being (WHO-5), work ability, and sleep dissatisfaction, suggesting a coherent but predominantly age-driven clustering structure. Mantel tests further highlighted contributions of physical activity and PSQI to the overall correlation architecture (Supplementary Table S22; Figure 4A).

In night NSW, a more complex and exposure-sensitive clustering pattern emerged. Age remained strongly associated with cardiovascular risk (IRCV; *ρ* = 0.82, *p* < 0.001) and metabolic indices (METS-IR ρ = 0.37, TyG ρ = 0.31, WyG ρ = 0.27; *p* < 0.001), but stronger and more widespread interconnections were observed between sleep disruption, work ability, and psychological well-being. In particular, PSQI acted as a central node within the network, showing robust correlations with sleep duration, WAI, WHO-5, and sleep dissatisfaction (*ρ* = 0.50–0.66, *p* < 0.001), indicating a more integrated sleep–behavior–health clustering pattern compared with DSW. Mantel analyses confirmed the relevance of physical activity, work ability, psychological well-being, and sleep quality in shaping the overall correlation structure (Supplementary Table S22; Figure 4A).

When stratified by cumulative exposure, higher exposure (≥500 lifetime night shifts) was associated with a strengthening and densification of the correlation network, particularly among sleep quality, cardiometabolic indices, and age-related variables (BMI *ρ* = 0.14 and IRCV ρ = 0.49, *p* ≤ 0.001). In this subgroup, sleep disturbances and metabolic alterations showed stronger interdependencies, whereas in low-exposure workers correlations were weaker and more dispersed. Mantel tests suggested a shift in network drivers, with cardiometabolic and sleep-related variables predominating in high-exposure workers, and physical activity playing a relatively greater role in low-exposure participants (Supplementary Table S22; Figure 4B).

Stratification by duration of exposure further supported a progressive reorganization of risk clustering. Short-term night shift workers (1–10 years) exhibited strong coupling between cumulative exposure metrics and metabolic indices (*ρ* > 0.87), alongside moderate associations between sleep impairment, reduced well-being, and lower work ability (ρ = −0.38 to −0.49). In contrast, long-term workers (≥11 years) showed a more pronounced integration of sleep disruption with psychological and occupational outcomes, with stronger associations between night shift duration, sleep quality, reduced well-being (ρ = −0.53), and lower work ability (ρ = −0.61). In this group, the influence of physical activity on the overall network structure was negligible (*p* = 0.77; Supplementary Table S22; Figure 4C).

Overall, cumulative and long-term night shift exposure were associated with a progressive reorganization of neurobehavioral and cardiometabolic risk clustering, characterized by increasingly interconnected sleep, lifestyle, psychological, occupational, and cardiometabolic domains at higher exposure levels.

## 4 Discussion

This cross-sectional study of 243 female healthcare workers provides a multidimensional characterization of health profiles associated with night shift work, integrating reproductive history, lifestyle behaviors, occupational exposure patterns, neurobehavioral outcomes, and cardiometabolic risk indicators. The cohort was largely composed of normal-weight individuals (DSW 65.6%, NSW 65.3%) with moderate adherence to the Mediterranean diet (DSW 67.7%, NSW 59.9%), but showed marked heterogeneity in occupational roles and exposure, with nurses predominating among night shift workers (62.8%) and a substantial percentage reporting long-term and high cumulative exposure (11–35 years: 42.9%; ≥500 lifetime shifts: 35.4%). This occupational structure is relevant, as nursing roles combine sustained psychosocial demands, physical workload, and circadian disruption, potentially amplifying exposure-related biological stressors within the female workforce (41–43).

Night shift work operated not as a single binary exposure but as a dynamic and cumulative construct, intersecting with age, reproductive history, and lifestyle behaviors (44). Indeed, higher cumulative exposure was associated with older age and a greater burden of reproductive life events, including pregnancy, childbirth, breastfeeding, and menopause (*p* < 0.05). These findings highlight the importance of considering life-course accumulation of both occupational and biological exposures in female healthcare workers, moving beyond traditional models that treat age or current shift status as isolated covariates (45–48).

Lifestyle factors also appeared embedded within this exposure structure. Smoking prevalence increased with night shift exposure (31.1% vs. 15.4%, *p* = 0.044), while physical activity decreased (low activity: 40.0% vs. 19.6%, *p* = 0.012), suggesting a gradual clustering of detrimental lifestyle habits along the night shift exposure gradient (49–52). Importantly, as demonstrated by the age-restricted analyses, these patterns were at least partially independent of age, supporting the interpretation that occupational exposure contributes to lifestyle reconfiguration rather than merely reflecting age-related differences (53, 54).

Sleep quality emerged as the most consistent and early manifestation of night shift exposure. Night shift workers reported poorer sleep quality than day shift workers (very poor sleep: 8.2% vs. 3.1%, *p* = 0.003), increasing with higher cumulative (≥500 shifts: 53.7% vs. 33.3%, *p* = 0.019) and long-term exposure (11–35 years: 61.9% vs. 34.5%, *p* = 0.001). Age-restricted analyses confirmed independence from age (17.4% vs. 4.6%, *p* = 0.019). In confounder-adjusted models, sleep impairment remained consistently associated with night shift regardless of whether exposure was operationalized as status, cumulative shifts, or years of work. This suggests that sleep disruption may represent a core and relatively exposure-insensitive feature of circadian misalignment, likely reflecting acute and chronic desynchronization of sleep–wake regulatory systems (6, 55–58).

In parallel, psychological well-being showed a worsening pattern with increasing cumulative and long-term exposure, with a higher prevalence of individuals at risk of depression in more highly exposed workers (13.7% vs. 0.0% for cumulative exposure; 15.9% vs. 3.6% for long-term exposure, *p* < 0.05), alongside a shift toward poorer well-being categories. This is consistent with evidence indicating that sleep disturbances may activate the hypothalamic–pituitary–adrenal (HPA) axis, leading to dysregulated cortisol secretion (59). Such neuroendocrine alterations have also been linked to adverse cardiometabolic outcomes, including insulin resistance and central adiposity (60). However, the lack of direct assessment of circadian and neuroendocrine biomarkers, such as melatonin and cortisol, should be considered when interpreting these proposed mechanistic pathways. Additionally, sleep loss may promote low-grade systemic inflammation, which could further contribute to both mood and metabolic alterations, potentially reinforcing these processes through a positive feedback loop (61).

In contrast, work ability showed a more heterogeneous pattern, with weaker associations overall and greater sensitivity to cumulative exposure rather than binary status. This supports the interpretation that occupational functioning is more dependent on accumulated workload and long-term adaptation processes, rather than on exposure *per se* (62, 63). This trend may potentially increase the risk of productivity loss and errors, especially in healthcare (64–66).

The cardiometabolic assessment was complemented by novel anthropometric and composite indices (WyG and IRCV), which may enhance the characterization of central adiposity in women and early cardiovascular risk beyond conventional BMI-based measures, highlighting their utility in occupational health surveillance and early disease prevention strategies (67). Specifically, metabolic evaluation was based on routine biochemical markers and surrogate indices (METS-IR, TyG, WyG, LDL/HDL, and TC/HDL), which should be interpreted as early indicators of metabolic risk rather than direct clinical outcomes of cardiovascular disease (68). Additionally, cardiovascular risk was assessed using IRCV, a validated composite score integrating established risk factors (including age, lipid profile, systolic blood pressure, antihypertensive treatment, and diabetes status), and therefore representing an early stratification tool rather than a definitive outcome measure (69). As shown by the regression analyses, while binary night shift status was not independently associated with metabolic indices in adjusted models, years of exposure showed consistent associations with insulin resistance markers (TyG and METS-IR), suggesting a duration-dependent physiological burden. Long-term night shift workers showed early unfavorable cardiometabolic patterns, including higher overweight/obesity (BMI ≥ 25 kg/m^2^: 42.9% vs. 26.2%, *p* = 0.034), central adiposity (WHtR >0.5: 71.4% vs. 53.6%, *p* = 0.028), and insulin resistance (TyG > 8.8: 11.1% vs. 0%, *p* = 0.043; WyG > 8.19: 63.8% vs. 45.1%, *p* = 0.029). If persistent, these alterations may promote low-grade inflammation and precede diabetes or dyslipidemia, especially in women (70–73). Intermediate cardiovascular risk was also elevated with longer night shift exposure (IRCV: 18.3% vs. 3.1%, *p* = 0.006), consistent with previous observations highlighting early vulnerability linked to shift work-related metabolic and vascular disturbances (74–77). Cumulative exposure showed intermediate effects, but with less stability across outcomes, indicating that simple aggregation of shifts may not fully capture biologically relevant exposure timing or intensity (5). Importantly, age, smoking, and menopausal status were among the strongest predictors of cardiometabolic indices, particularly IRCV, reinforcing the need for confounder-aware interpretation of occupational effects in female populations (78). This finding is consistent with the established role of menopausal transition in modulating metabolic regulation and cardiovascular risk trajectories in women (79).

Correlation analyses further highlight exposure-dependent clustering of health outcomes. For instance, higher cumulative or long-term exposure correlated with poorer sleep quality (PSQI *ρ* = 0.31–0.32), shorter sleep (*ρ* = −0.25), lower psychological well-being (WHO-5 ρ = −0.50 to −0.57), reduced work ability (WAI ρ = −0.36 to −0.64), and modest increases in BMI (ρ = 0.14–0.20), WHtR (ρ = 0.11–0.15), insulin resistance (METS-IR ρ = 0.17, TyG ρ = 0.21, WyG ρ = 0.21), and cardiovascular risk (IRCV ρ = 0.49; all *p* ≤ 0.001). Rather than isolated associations, night shift exposure was linked with a reorganization of health-related clustering, particularly in higher exposure groups. Once again, sleep quality emerged as a central node linking neurobehavioral outcomes (work ability, psychological well-being) and early cardiometabolic markers (80). In highly exposed workers, these interconnections became denser: dose–response patterns were strongest among high cumulative (PSQI–WAI ρ = −0.64; PSQI–WHO-5 ρ = −0.57) and long-term workers (well-being ρ = −0.53; WAI ρ = −0.61), suggesting a convergence of sleep disruption, functional impairment, and metabolic dysregulation into an integrated risk phenotype (81). This pattern might be consistent with an accumulation model, in which repeated circadian and psychosocial stressors progressively reshape the structure of health interactions (82). Within this framework, sleep satisfaction has been increasingly conceptualized not only as an outcome of occupational stress but also as a key mediating factor linking chronic work-related exposures to reduced work ability. A recent meta-analysis in nursing populations supports this interpretation, indicating that impaired sleep is strongly associated with burnout-related processes and contributes to reduced work ability and functional decline (83). In addition, long duration and high cumulative night shift exposure may further amplify cardiometabolic risk through behavioral pathways, as circadian misalignment and time constraints can plausibly reduce physical activity levels and limit adherence to a Mediterranean dietary pattern. This may contribute to the multi-layered risk profile observed in highly exposed workers, in which occupational constraints interact with behavioral adaptations, reinforcing metabolic vulnerability over time (84, 85).

Overall, this study supports a framework in which night shift work is associated with a reorganization of sleep, behavioral, and cardiometabolic domains, with sleep disturbance emerging as an early pathway linking exposure to downstream functional and metabolic alterations (86, 87). Circadian misalignment likely mediates these effects, as experimental studies show disrupted rhythms can alter insulin, cortisol, and leptin, promoting insulin resistance and central adiposity (88).

These results reinforce an important methodological implication: the interpretation of night shift effects depends strongly on how exposure is defined (89). As corroborated by our data, binary status captures acute circadian misalignment effects, cumulative exposure reflects behavioral and functional outcomes, and, finally, duration of exposure better captures metabolic embedding and physiological adaptation processes. This highlights the importance of adopting multidimensional exposure metrics in occupational research. This multidimensional operationalization is also consistent with recent large-scale exposome-oriented initiatives such as the EPHOR-NIGHT cohort, which combined information on shift type (i.e., permanent day, permanent night, and rotating schedules), years of night work, frequency of night shifts, long shifts, rest periods, and objective measures of sleep and light exposure to better characterize biologically relevant patterns of circadian disruption and chronic disease risk among healthcare workers (21). In line with this perspective, future studies could further refine exposure assessment by using more granular exposure categories, schedule intensity indicators, and repeated objective measures of circadian disruption.

Strengths of this study include its multidimensional design, combining occupational exposure quantification with reproductive, behavioral, neurobehavioral, and cardiometabolic domains, as well as the use of multiple complementary analytical approaches, including regression modeling, stratified analyses, and correlation matrices. This allowed for a systems-level characterization of health effects rather than isolated outcome assessment.

Limitations include the cross-sectional design, which precludes causal inference; reliance on self-reported measures for some behavioral variables; restriction to female healthcare workers, which limits generalizability; and potential “healthy worker” selection bias, whereby individuals intolerant to night work may have left exposure earlier or changed schedules, or exited night shift roles due to emerging health conditions, possibly leading to an underestimation of exposure-related effects (90). Additionally, some variables included a percentage of missing or “not answering” responses, particularly for occupational role and hormone-related factors. Although analyses were conducted using complete-case approaches and missingness was generally limited, these incomplete responses could have slightly affected estimates and should be considered when interpreting the findings. Also, residual confounding cannot be excluded despite adjustment for key covariates. Finally, recency and intensity of night shift exposure were not included in multivariable models due to limited additional explanatory value and sample size constraints within exposure strata. Future studies should adopt more comprehensive exposure models within longitudinal designs.

Although the present study primarily focused on occupational history and internal neurobehavioral and cardiometabolic responses, additional external occupational exposures potentially relevant to circadian disruption, including workplace light-at-night intensity, environmental stressors, and chemical exposures, were not directly assessed. Future research integrating objective environmental and biological exposure measurements may help to further clarify the complex interactions between shift work, circadian disruption, and chronic disease risk within a multilayered life-course occupational exposome context (21). In particular, the use of wearable and passive sampling technologies could allow a more comprehensive assessment of environmental and occupational dimensions, including light-at-night exposure, noise, temperature, and objective measures of sleep and physical activity (82).

From a public health and occupational medicine perspective, these findings support a shift from a binary classification of night work toward a cumulative and life-course exposure assessment. The results provide evidence-based insights for the development of targeted occupational health strategies, emphasizing a multilevel preventive approach. At the primary prevention level, interventions should focus on optimizing shift scheduling and reducing excessive night shift burden, with the aim of minimizing circadian disruption, alongside the promotion of healthy lifestyle behaviors such as physical activity, sleep hygiene, and dietary regularity (66, 91–93). At the secondary level, early identification of sleep disturbances emerges as a key priority, given their role as an early and consistent marker of neurobehavioral impairment, including depressive symptomatology, as well as functional outcomes (94). In parallel, surveillance of metabolic risk trajectories should be considered, particularly among long-term exposed workers, to enable timely detection of early cardiometabolic alterations (95). Within this context, sleep quality represents a central target for preventive action, while periodic monitoring of metabolic markers may support early risk stratification in high-exposure female healthcare workers (66, 96).

## 5 Conclusion

These findings support multilayered framework in which night shift work contributes to the progressive accumulation and interaction of occupational, behavioral, and biological risk factors, ultimately leading to an integrated clustering of neurobehavioral and cardiometabolic indicators. The identification of early, subclinical alterations, particularly in sleep quality and insulin resistance-related indices, highlights their potential value as intermediate markers for risk stratification in occupational settings.

While causality cannot be established due to the cross-sectional design, this multidimensional approach emphasizes the need to move beyond simplistic binary classifications of exposure toward cumulative and life-course measurements. From a public health and occupational medicine perspective, these findings reinforce the importance of implementing targeted, exposure-based strategies, including enhanced surveillance, circadian-aware scheduling, early detection of sleep disturbances, and promotion of healthy behaviors, especially among long-term exposed female workers.

## Data Availability

The raw data supporting the conclusions of this article will be made available by the authors, without undue reservation.
