# Supplementary material for: Night shift work exposure shapes neurobehavioral and cardiometabolic profiles in female healthcare workers: a cross-sectional study
Source: Front Public Health. 2026 Jun 10;14:1860519. doi: 10.3389/fpubh.2026.1860519 (PMC13290798; doi:10.3389/fpubh.2026.1860519)
Supplement: Supplementary file 1 [file Data_Sheet_1.docx]

Night shift work exposure shapes neurobehavioral and cardiometabolic profiles in female healthcare workers: a cross-sectional study

Silvia Vivarelli^1*^, Tania Formica^1^, Francesca Simona Fiorino^1^, Manuela Pollicino^1^, Saveria Savasta^1^, Caterina Oliveri^1^, Laura Trifilò^1^, Federica Giambò^2^, Concettina Fenga^1^

^1^ Department of Biomedical and Dental Sciences, Morphological and Functional Imaging, Section of Occupational Medicine, University of Messina, Italy

^2^ Vita-Salute San Raffaele University, Milan, Italy

*** Correspondence:**Silvia Vivarelli
[silvia.vivarelli@unime.it](mailto:silvia.vivarelli@unime.it)

Supplementary Material

# Supplementary Tables

**Supplementary Table S1. Neurobehavioral questionnaires: domains, scoring, and cut-offs**

| **Instrument** | **Domain assessed** | **Score range** | **Categories / cut-offs used** | **Binary classification** | **Reference** |
| --- | --- | --- | --- | --- | --- |
| Medi-Lite | Mediterranean diet adherence | 0–18 | Low: 0–7; Medium: 8–11; High: 12–18 | ≤11 vs >11 | (1) |
| IPAQ | Physical activity (MET-min/week) | Continuous | Low: <600; Moderate: 600–3000; High: >3000 | ≤3000 vs >3000 | (2) |
| PSQI | Sleep quality | 0–21 | Poor sleep: >5 | ≤5 vs >5 | (3) |
| WHO-5 | Psychological well-being | 0–100 | Reduced well-being: <50; Possible depression: ≤28 | ≥50 vs <50 | (4) |
| WAI | Work ability | 7–49 | Poor: 7–27; Moderate: 28–36; Good: 37–43; Excellent: 44–49 | ≤36 vs >36 | (5) |

**Supplementary Table S2. Composite anthropometric, metabolic, and cardiovascular indices: formulas and cut-offs**

| **Index** | **Domain** | **Formula / Definition** | **Categories / cut-offs used** | **Binary classification** | **Reference** |
| --- | --- | --- | --- | --- | --- |
| BMI | General adiposity | Weight (kg) / height² (m²) | WHO: <18.5; 18.5–24.9; 25–29.9; 30–34.9; 35–39.9; ≥40 kg/m² | <25 vs ≥25 kg/m² | (6) |
| WHtR | Central adiposity | Waist circumference (cm) / height (cm) | — | ≤0.5 vs >0.5 | (7) |
| METS-IR | Insulin resistance | ln[(2 × fasting glucose) + triglycerides] × BMI / ln(HDL-C) | Low <30; Moderate 30–45; High >45 | ≤45 vs >45 | (8) |
| TyG | Insulin resistance | ln[triglycerides × fasting glucose / 2] | Low <8.5; Medium 8.5–8.8; High >8.8 | ≤8.8 vs >8.8 | (9) |
| WyG | Insulin resistance / central adiposity | ln[waist circumference × fasting glucose / 2] | — | ≤8.19 vs >8.19 (females) | (10) |
| LDL/HDL | Atherogenic lipid ratio | LDL cholesterol / HDL cholesterol | <2; 2–3; >3 | ≤3 vs >3 | (11) |
| TC/HDL | Atherogenic lipid ratio | Total cholesterol / HDL cholesterol | <3.5; 3.5–5; >5 | ≤5 vs >5 | (11) |
| IRCV | Cardiovascular risk | 100 × (1 − 0.889^(−RF)) | Low <3%; Intermediate 3–20%; High >20% | — | (12,13) |

RF = weighted combination of age, total cholesterol, HDL cholesterol, systolic blood pressure, antihypertensive treatment, sex, and diabetes status

**Supplementary Table S3. Anthropometric characteristics of female healthcare workers by work schedule**

| **Variable** | **Total (N=243), Mean ± SD** | **DSW (N=96), Mean ± SD** | **NSW (N=147), Mean ± SD** | **p-value** |
| --- | --- | --- | --- | --- |
| Age (years) | 43.3 ± 12.2 | 40.1 ± 13.0 | 45.5 ± 11.1 | **<0.001** |
| Weight (kg) | 62.4 ± 11.2 | 60.8 ± 10.7 | 63.4 ± 11.4 | **0.076** |
| Height (m) | 1.62 ± 0.06 | 1.62 ± 0.06 | 1.62 ± 0.06 | 0.950 |
| Waist circumference (cm) | 84.7 ± 10.0 | 83.3 ± 10.0 | 85.7 ± 9.9 | **0.025** |

**Supplementary Table S4. Distribution of age categories among female healthcare workers by work schedule**

| **Age category (years)** | **Total N (%)** | **DSW N (%)** | **NSW N (%)** | **χ²** | **p-value** |
| --- | --- | --- | --- | --- | --- |
| <30 | 46 (18.9) | 31 (32.3) | 15 (10.2) | 24.327 | **<0.001** |
| 30–39 | 53 (21.8) | 22 (22.9) | 31 (21.1) | — | — |
| 40–49 | 54 (22.2) | 18 (18.8) | 36 (24.5) | — | — |
| 50–59 | 64 (26.3) | 14 (14.6) | 50 (34.0) | — | — |
| ≥60 | 26 (10.7) | 11 (11.5) | 15 (10.2) | — | — |

**Supplementary Table S5. Reproductive, lifestyle, and behavioral characteristics of female healthcare workers by work schedule**

| **Variable** | **Category** | **Total N (%)** | **DSW N (%)** | **NSW N (%)** | **χ²** | **p-value** |
| --- | --- | --- | --- | --- | --- | --- |
| **Pregnancy** | No | 109 (44.9) | 59 (61.5) | 50 (34.0) | 17.685 | **<0.001** |
|  | Yes | 134 (55.1) | 37 (38.5) | 97 (66.0) | — | — |
| **Number of children** | None | 109 (44.9) | 59 (61.5) | 50 (34.0) | 18.033 | **<0.001** |
|  | 1 | 53 (21.8) | 13 (13.5) | 40 (27.2) | — | — |
|  | ≥2 | 81 (33.3) | 24 (25.0) | 57 (38.8) | — | — |
| **Breastfeeding** | No | 128 (52.7) | 62 (64.6) | 66 (44.9) | 9.028 | **0.003** |
|  | Yes | 115 (47.3) | 34 (35.4) | 81 (55.1) | — | — |
| **Menopause** | No | 173 (71.2) | 77 (80.2) | 96 (65.3) | 6.289 | **0.012** |
|  | Yes | 70 (28.8) | 19 (19.8) | 51 (34.7) | — | — |
| **Contraceptives** | No | 110 (45.3) | 49 (51.0) | 61 (41.5) | 8.615 | **0.013** |
|  | Yes in the past | 24 (9.9) | 14 (14.6) | 10 (6.8) | — | — |
|  | Yes currently | 109 (44.9) | 33 (34.4) | 76 (51.7) | — | — |
| **Hormone therapy** | No | 233 (97.1) | 94 (98.9) | 139 (95.9) | 0.899 | 0.638 |
|  | Yes in the past | 5 (2.1) | 1 (1.1) | 4 (2.8) | — | — |
|  | Yes currently | 2 (0.8) | 0 (0.0) | 2 (1.4) | — | — |
|  | Not answering | 3 (1.2) | 1 (1.0) | 2 (1.4) | — | — |
| **Smoking** | No | 152 (62.6) | 69 (71.9) | 83 (56.5) | 7.105 | **0.029** |
|  | Yes in the past | 29 (11.9) | 11 (11.5) | 18 (12.2) | — | — |
|  | Yes currently | 62 (25.5) | 16 (16.7) | 46 (31.3) | — | — |
| **Alcohol drinking** | No | 74 (30.5) | 30 (31.3) | 44 (29.9) | 1.174 | 0.827 |
|  | Yes, occasionally | 160 (65.8) | 64 (66.7) | 96 (65.3) | — | — |
|  | Yes, regularly | 9 (3.7) | 2 (2.1) | 7 (4.8) | — | — |
| **Regular physical activity** | No | 138 (56.8) | 55 (57.3) | 83 (56.5) | 0.016 | 0.899 |
|  | Yes | 105 (43.2) | 41 (42.7) | 64 (43.5) | — | — |
| **Sleep quality** | Very good | 29 (11.9) | 17 (17.7) | 12 (8.2) | 14.221 | **0.003** |
|  | Fairly good | 107 (44.0) | 46 (47.9) | 61 (41.5) | — | — |
|  | Poor | 80 (32.9) | 30 (31.3) | 50 (34.0) | — | — |
|  | Very poor | 27 (11.1) | 3 (3.1) | 24 (16.3) | — | — |

**Supplementary Table S6. Distribution of job roles among female healthcare workers by work schedule**

| **Job role** | **Total N (%)** | **DSW N (%)** | **NSW N (%)** | **χ²** | **p-value** |
| --- | --- | --- | --- | --- | --- |
| Healthcare executive | 35 (16.3) | 32 (41.0) | 3 (2.2) | 73.867 | **<0.001** |
| Physician | 51 (23.7) | 21 (26.9) | 30 (21.9) | — | — |
| Nurse | 97 (45.1) | 11 (14.1) | 86 (62.8) | — | — |
| Nursing assistant | 18 (8.4) | 7 (9.0) | 11 (8.0) | — | — |
| Healthcare technician | 14 (6.5) | 7 (9.0) | 7 (5.1) | — | — |
| Not answering | 28 (11.5) | 18 (18.8) | 10 (6.8) | — | — |

**Supplementary Table S7. Patterns and duration of night shift work exposure among female healthcare workers**

| **Variable** | **Category** | **N (%)** |
| --- | --- | --- |
| **NS exposure duration (years)** | Short (1–10 years) | 84 (57.1) |
|  | Long (11–35 years) | 63 (42.9) |
| **Recent exposure (average number of NS/month in last 6 months)** | Low (<6 night shifts/month) | 106 (72.1) |
|  | High (≥6 night shifts/month) | 41 (27.9) |
| **Cumulative exposure (number of lifetime NS)** | Low (<500 lifetime night shifts) | 95 (64.6) |
|  | High (≥500 lifetime night shifts) | 52 (35.4) |
| **Regular NSW period** | Low (<4 night shifts/month) | 13 (8.8) |
|  | Medium (≥4 night shifts/month for 1–9 years) | 54 (36.7) |
|  | High (≥4 night shifts/month for ≥10 years) | 80 (54.5) |

**Supplementary Table S8. Anthropometric characteristics of female healthcare workers aged ≥30 years by work schedule**

| **Variable** | **Total (N=197) Mean ± SD** | **DSW (N=65) Mean ± SD** | **NSW (N=132) Mean ± SD** | **p-value** |
| --- | --- | --- | --- | --- |
| Age (years) | 47.1 ± 10.2 | 46.3 ± 11.3 | 47.6 ± 9.7 | 0.387 |
| Weight (kg) | 63.4 ± 11.3 | 62.4 ± 10.0 | 64.0 ± 11.9 | 0.457 |
| Height (m) | 1.62 ± 0.06 | 1.63 ± 0.06 | 1.62 ± 0.06 | 0.356 |
| Waist circumference (cm) | 85.6 ± 10.0 | 84.4 ± 9.8 | 86.3 ± 10.1 | 0.162 |

**Supplementary Table S9. Distribution of age categories among female healthcare workers aged ≥30 years by work schedule**

| **Age category (years)** | **Total N (%)** | **DSW N (%)** | **NSW N (%)** | **χ²** | **p-value** |
| --- | --- | --- | --- | --- | --- |
| 30–39 | 53 (21.8) | 22 (22.9) | 31 (21.1) | 6.340 | 0.096 |
| 40–49 | 54 (22.2) | 18 (18.8) | 36 (24.5) | — | — |
| 50–59 | 64 (26.3) | 14 (14.6) | 50 (34.0) | — | — |
| ≥60 | 26 (10.7) | 11 (11.5) | 15 (10.2) | — | — |

**Supplementary Table S10. Reproductive, lifestyle, and behavioral characteristics of female healthcare workers aged ≥30 years by work schedule**

| **Variable** | **Category** | **Total N (%)** | **DSW N (%)** | **NSW N (%)** | **χ²** | **p-value** |
| --- | --- | --- | --- | --- | --- | --- |
| **Pregnancy** | No | 63 (32.0) | 28 (43.1) | 35 (26.5) | 5.492 | **0.019** |
|  | Yes | 134 (68.0) | 37 (56.9) | 97 (73.5) | — | — |
| **Number of children** | None | 63 (32.0) | 28 (43.1) | 35 (26.5) | 5.869 | **0.053** |
|  | 1 | 53 (26.9) | 13 (20.0) | 40 (30.3) | — | — |
|  | ≥2 | 81 (41.1) | 24 (36.9) | 57 (43.2) | — | — |
| **Breastfeeding** | No | 82 (41.6) | 31 (47.7) | 51 (38.6) | 1.470 | 0.225 |
|  | Yes | 115 (58.4) | 34 (52.3) | 81 (61.4) | — | — |
| **Menopause** | No | 127 (64.5) | 46 (70.8) | 81 (61.4) | 1.682 | 0.195 |
|  | Yes | 70 (35.5) | 19 (29.2) | 51 (38.6) | — | — |
| **Contraceptives** | No | 89 (45.2) | 35 (53.8) | 54 (40.9) | 5.200 | **0.074** |
|  | Yes in the past | 13 (6.6) | 6 (9.2) | 7 (5.3) | — | — |
|  | Yes currently | 95 (48.2) | 24 (36.9) | 71 (53.8) | — | — |
| **Hormone therapy** | No | 187 (96.4) | 63 (98.4) | 124 (95.4) | 1.408 | 0.495 |
|  | Yes in the past | 5 (2.6) | 1 (1.6) | 4 (3.1) | — | — |
|  | Yes currently | 2 (1.0) | 0 (0.0) | 2 (1.5) | — | — |
|  | Not answering | 3 (1.2) | 1 (1.0) | 2 (1.4) | — | — |
| **Smoking** | No | 120 (60.9) | 47 (72.3) | 73 (55.3) | 6.260 | **0.044** |
|  | Yes in the past | 26 (13.2) | 8 (12.3) | 18 (13.6) | — | — |
|  | Yes currently | 51 (25.9) | 10 (15.4) | 41 (31.1) | — | — |
| **Alcohol drinking** | No | 70 (35.5) | 26 (40.0) | 44 (33.3) | 1.161 | 0.560 |
|  | Yes, occasionally | 118 (59.9) | 37 (56.9) | 81 (61.4) | — | — |
|  | Yes, regularly | 9 (4.6) | 2 (3.1) | 7 (5.3) | — | — |
| **Regular physical activity** | No | 122 (61.9) | 43 (66.2) | 79 (59.8) | 0.734 | 0.391 |
|  | Yes | 75 (38.1) | 22 (33.8) | 53 (40.2) | — | — |
| **Sleep quality** | Very good | 20 (10.2) | 11 (16.9) | 9 (6.8) | 9.952 | **0.019** |
|  | Fairly good | 80 (40.6) | 28 (43.1) | 52 (39.4) | — | — |
|  | Poor | 71 (36.0) | 23 (35.4) | 48 (36.4) | — | — |
|  | Very poor | 26 (13.2) | 3 (4.6) | 23 (17.4) | — | — |

**Supplementary Table S11. Distribution of job roles among female healthcare workers aged ≥30 years by work schedule**

| **Job role** | **Total N (%)** | **DSW N (%)** | **NSW N (%)** | **χ²** | **p-value** |
| --- | --- | --- | --- | --- | --- |
| Healthcare executive | 24 (13.4) | 21 (39.6) | 3 (2.4) | 54.319 | **<0.001** |
| Physician | 35 (19.6) | 9 (17.0) | 26 (20.6) | — | — |
| Nurse | 91 (50.8) | 11 (20.8) | 80 (63.5) | — | — |
| Nursing assistant | 18 (10.1) | 7 (13.2) | 11 (8.7) | — | — |
| Healthcare technician | 11 (6.1) | 5 (9.4) | 6 (4.8) | — | — |
| Not answering | 18 (9.1) | 12 (18.5) | 6 (4.5) | — | — |

**Supplementary Table S12. Patterns and duration of night shift work exposure among female healthcare workers aged ≥30 years**

| **Variable** | **Category** | **N (N%)** |
| --- | --- | --- |
| **NS exposure duration (years)** | Short (1–10 years) | 69 (52.3) |
|  | Long (11–35 years) | 63 (47.7) |
| **Recent exposure (average number of NS/month in last 6 months)** | Low (<6 night shifts/month) | 96 (72.7) |
|  | High (≥6 night shifts/month) | 36 (27.3) |
| **Cumulative exposure (number of lifetime NS)** | Low (<500 lifetime night shifts) | 38 (28.8) |
|  | High (≥500 lifetime night shifts) | 94 (71.2) |
| **Regular NSW period** | Low (<4 night shifts/month) | 7 (5.3) |
|  | Medium (≥4 night shifts/month for 1–9 years) | 45 (34.1) |
|  | High (≥4 night shifts/month for ≥10 years) | 80 (60.6) |

**Supplementary Table S13. Distribution of Medi-Lite score categories by work schedule, age, cumulative night shift exposure, and duration of night shift work**

| **DSW / NSW (overall)** | **n / n (% / %)** | **χ²** | **p-value** |
| --- | --- | --- | --- |
| Low (0–7) | 8 / 11 (8.3% / 7.5%) | 2.124 | 0.346 |
| Medium (8–11) | 65 / 88 (67.7% / 59.9%) | – | – |
| High (12–18) | 23 / 48 (24.0% / 32.7%) | – | – |
| Low/Medium (0–11) vs High (12–18) | 73 / 99 (76.0% / 67.3%) | 2.123 | 0.145 |
| **DSW / NSW (≥30 years)** |  |  |  |
| Low (0–7) | 8 / 10 (12.3% / 7.6%) | 1.878 | 0.391 |
| Medium (8–11) | 41 / 80 (63.1% / 60.6%) | – | – |
| High (12–18) | 16 / 42 (24.6% / 31.8%) | – | – |
| Low/Medium (0–11) vs High (12–18) | 49 / 90 (75.4% / 68.2%) | 1.088 | 0.297 |
| **NSW (Low / High)** |  |  |  |
| Low (0–7) | 5 / 6 (9.8% / 6.3%) | 0.719 | 0.698 |
| Medium (8–11) | 29 / 59 (56.9% / 62.1%) | – | – |
| High (12–18) | 17 / 30 (33.3% / 31.6%) | – | – |
| Low/Medium (0–11) vs High (12–18) | 34 / 65 (66.7% / 68.4%) | 0.047 | 0.829 |
| **NSW (Short / Long)** |  |  |  |
| Low (0–7) | 6 / 5 (7.1% / 7.9%) | 0.062 | 0.970 |
| Medium (8–11) | 50 / 38 (59.5% / 60.3%) | – | – |
| High (12–18) | 28 / 20 (33.3% / 31.7%) | – | – |
| Low/Medium (0–11) vs High (12–18) | 56 / 43 (66.7% / 68.3%) | 0.041 | 0.839 |

**Supplementary Table S14. Distribution of physical activity levels (IPAQ) by work schedule, age, cumulative night shift exposure, and duration of night shift work**

| **DSW / NSW (overall)** | **n / n (% / %)** | **χ²** | **p-value** |
| --- | --- | --- | --- |
| Low (<600) | 29 / 48 (30.2% / 32.7%) | 2.162 | 0.339 |
| Medium (600–3000) | 36 / 64 (37.5% / 43.5%) | – | – |
| High (>3000) | 31 / 35 (32.3% / 23.8%) | – | – |
| Low (<600) vs Medium/High (≥600) | 67 / 99 (69.8% / 67.3%) | 0.1603 | 0.689 |
| Low/Medium (≤3000) vs High (>3000) | 65 / 112 (67.7% / 76.2%) | 2.112 | 0.146 |
| **DSW / NSW (≥30 years)** |  |  |  |
| Low (<600) | 17 / 47 (26.2% / 35.6%) | 4.498 | 0.106 |
| Medium (600–3000) | 23 / 53 (35.4% / 40.2%) | – | – |
| High (>3000) | 25 / 32 (38.5% / 24.2%) | – | – |
| Low (<600) vs Medium/High (≥600) | 48 / 85 (73.8% / 64.4%) | 1.774 | 0.183 |
| Low/Medium (≤3000) vs High (>3000) | 40 / 100 (61.5% / 75.8%) | 4.282 | **0.039** |
| **NSW (Low / High)** |  |  |  |
| Low (<600) | 10 / 38 (19.6% / 40.0%) | 8.848 | **0.012** |
| Medium (600–3000) | 30 / 33 (58.8% / 34.7%) | – | – |
| High (>3000) | 11 / 24 (21.6% / 25.3%) | – | – |
| Low (<600) vs Medium/High (≥600) | 41 / 57 (80.4% / 60.0%) | 6.25 | **0.012** |
| Low/Medium (≤3000) vs High (>3000) | 40 / 71 (78.4% / 74.7%) | 0.249 | 0.618 |
| **NSW (Short / Long)** |  |  |  |
| Low (<600) | 23 / 25 (27.4% / 39.7%) | 4.772 | 0.092 |
| Medium (600–3000) | 43 / 21 (51.2% / 33.3%) | – | – |
| High (>3000) | 18 / 17 (21.4% / 27.0%) | – | – |
| Low (<600) vs Medium/High (≥600) | 61 / 38 (72.6% / 60.3%) | 2.001 | 0.115 |
| Low/Medium (≤3000) vs High (>3000) | 66 / 46 (78.6% / 73.0%) | 0.613 | 0.434 |

**Supplementary Table S15. Distribution of sleep quality categories (PSQI) by work schedule, age, cumulative night shift exposure, and duration of night shift work**

| **DSW / NSW (overall)** | **n / n (% / %)** | **χ²** | **p-value** |
| --- | --- | --- | --- |
| Good (0–5) | 64 / 79 (66.7% / 53.7%) | 4.504 | 0.105 |
| Poor (6–10) | 29 / 58 (30.2% / 39.5%) | – | – |
| Very Poor (11–21) | 3 / 10 (3.1% / 6.8%) | – | – |
| Good (0–5) vs Poor/Very Poor (6–21) | 32 / 68 (33.3% / 46.3%) | 4.006 | **0.045** |
| **DSW / NSW (≥30 years)** |  |  |  |
| Good (0–5) | 39 / 68 (60.0% / 51.5%) | 1.358 | 0.507 |
| Poor (6–10) | 23 / 55 (35.4% / 41.7%) | – | – |
| Very Poor (11–21) | 3 / 9 (4.6% / 6.8%) | – | – |
| Good (0–5) vs Poor/Very Poor (6–21) | 26 / 64 (40.0% / 48.5%) | 1.264 | 0.261 |
| **NSW (Low / High)** |  |  |  |
| Good (0–5) | 34 / 44 (66.7% / 46.3%) | 8.747 | **0.013** |
| Poor (6–10) | 17 / 41 (33.3% / 43.2%) | – | – |
| Very Poor (11–21) | 0 / 10 (0.0% / 10.5%) | – | – |
| Good (0–5) vs Poor/Very Poor (6–21) | 17 / 51 (33.3% / 53.7%) | 5.523 | **0.019** |
| **NSW (Short / Long)** |  |  |  |
| Good (0–5) | 55 / 24 (65.5% / 38.1%) | 11.622 | **0.003** |
| Poor (6–10) | 26 / 32 (31.0% / 50.8%) | – | – |
| Very Poor (11–21) | 3 / 7 (3.6% / 11.1%) | – | – |
| Good (0–5) vs Poor/Very Poor (6–21) | 29 / 39 (34.5% / 61.9%) | 10.857 | **0.001** |

**Supplementary Table S16. Distribution of psychological well-being categories and depression risk (WHO-5) by work schedule, age, cumulative night shift exposure, and duration of night shift work**

| **DSW / NSW (overall)** | **n / n (% / %)** | **χ²** | **p-value** |
| --- | --- | --- | --- |
| Very Poor (0–24) | 2 / 10 (2.1% / 6.8%) | 6.843 | 0.077 |
| Poor (25–50) | 17 / 33 (17.7% / 22.4%) | – | – |
| Good (51–75) | 46 / 49 (47.9% / 33.3%) | – | – |
| Very Good (76–100) | 31 / 55 (32.3% / 37.4%) | – | – |
| Very Poor/Poor (0–50) | 19 / 43 (19.8% / 29.3%) | 2.735 | 0.098 |
| Good/Very Good (51–100) | 77 / 104 (80.2% / 70.7%) | – | – |
| At risk of depression (≤28) | 3 / 13 (3.1% / 8.8%) | 3.088 | 0.079 |
| No risk of depression (29–100) | 93 / 134 (96.9% / 91.2%) | – | – |
| **DSW / NSW (≥30 years)** |  |  |  |
| Very Poor (0–24) | 2 / 9 (3.1% / 6.8%) | 3.466 | 0.325 |
| Poor (25–50) | 14 / 30 (21.5% / 22.7%) | – | – |
| Good (51–75) | 29 / 43 (44.6% / 32.6%) | – | – |
| Very Good (76–100) | 20 / 50 (30.8% / 37.9%) | – | – |
| Very Poor/Poor (0–50) | 16 / 39 (24.6% / 29.5%) | 0.526 | 0.468 |
| Good/Very Good (51–100) | 49 / 93 (75.4% / 70.5%) | – | – |
| At risk of depression (≤28) | 3 / 12 (4.6% / 9.1%) | 1.24 | 0.265 |
| No risk of depression (29–100) | 62 / 120 (95.4% / 90.9%) | – | – |
| **NSW (Low / High)** |  |  |  |
| Very Poor (0–24) | 0 / 10 (0.0% / 10.5%) | 7.608 | 0.055 |
| Poor (25–50) | 11 / 22 (21.6% / 23.2%) | – | – |
| Good (51–75) | 22 / 27 (43.1% / 28.4%) | – | – |
| Very Good (76–100) | 18 / 36 (35.3% / 37.9%) | – | – |
| Very Poor/Poor (0–50) | 11 / 32 (21.6% / 33.7%) | 2.344 | 0.126 |
| Good/Very Good (51–100) | 40 / 63 (78.4% / 66.3%) | – | – |
| At risk of depression (≤28) | 0 / 13 (0.0% / 13.7%) | 7.661 | **0.006** |
| No risk of depression (29–100) | 51 / 82 (100% / 86.3%) | – | – |
| **NSW (Short / Long)** |  |  |  |
| Very Poor (0–24) | 2 / 8 (2.4% / 12.7%) | 8.91 | **0.031** |
| Poor (25–50) | 16 / 17 (19.0% / 27.0%) | – | – |
| Good (51–75) | 33 / 16 (39.3% / 25.4%) | – | – |
| Very Good (76–100) | 33 / 22 (39.3% / 34.9%) | – | – |
| Very Poor/Poor (0–50) | 18 / 25 (21.4% / 39.7%) | 5.796 | **0.016** |
| Good/Very Good (51–100) | 66 / 38 (78.6% / 60.3%) | – | – |
| At risk of depression (≤28) | 3 / 10 (3.6% / 15.9%) | 6.758 | **0.009** |
| No risk of depression (29–100) | 81 / 53 (96.4% / 84.1%) | – | – |

**Supplementary Table S17. Distribution of work ability categories (WAI) and type of effort by work schedule, age, cumulative night shift exposure, and duration of night shift work**

| **DSW / NSW (overall)** | **n / n (% / %)** | **χ²** | **p-value** |
| --- | --- | --- | --- |
| Poor (7–27) | 2 / 3 (2.1% / 2.0%) | 1.449 | 0.694 |
| Moderate (28–36) | 8 / 19 (8.3% / 12.9%) | – | – |
| Good (37–43) | 51 / 78 (53.1% / 53.1%) | – | – |
| Excellent (44–49) | 35 / 47 (36.5% / 32.0%) | – | – |
| Poor/Moderate (7–36) | 10 / 22 (10.4% / 15.0%) | 1.051 | 0.305 |
| Good/Excellent (37–49) | 86 / 125 (89.6% / 85.0%) | – | – |
| Mental Effort | 31 / 25 (32.3% / 17.0%) | 9.175 | **0.010** |
| Physical Effort | 4 / 3 (4.2% / 2.0%) | – | – |
| Mental and Physical Effort | 61 / 119 (63.5% / 81.0%) | – | – |
| **DSW / NSW (≥30 years)** |  |  |  |
| Poor (7–27) | 2 / 3 (3.1% / 2.3%) | 0.144 | 0.986 |
| Moderate (28–36) | 8 / 17 (12.3% / 12.9%) | – | – |
| Good (37–43) | 36 / 72 (55.4% / 54.5%) | – | – |
| Excellent (44–49) | 19 / 40 (29.2% / 30.3%) | – | – |
| Poor/Moderate (7–36) | 10 / 20 (15.4% / 15.2%) | 0.002 | 0.999 |
| Good/Excellent (37–49) | 55 / 112 (84.6% / 84.8%) | – | – |
| Mental Effort | 20 / 22 (30.8% / 16.7%) | 9.162 | **0.010** |
| Physical Effort | 4 / 2 (6.2% / 1.5%) | – | – |
| Mental and Physical Effort | 41 / 108 (63.1% / 81.8%) | – | – |
| **NSW (Low / High)** |  |  |  |
| Poor (7–27) | 0 / 3 (0.0% / 3.2%) | 5.458 | 0.141 |
| Moderate (28–36) | 3 / 16 (5.9% / 16.8%) | – | – |
| Good (37–43) | 30 / 48 (58.8% / 50.5%) | – | – |
| Excellent (44–49) | 18 / 28 (35.3% / 29.5%) | – | – |
| Poor/Moderate (7–36) | 3 / 19 (5.9% / 20.0%) | 5.168 | **0.023** |
| Good/Excellent (37–49) | 48 / 76 (94.1% / 80.0%) | – | – |
| Mental Effort | 9 / 15 (17.6% / 15.8%) | 0.085 | 0.958 |
| Physical Effort | 1 / 2 (2.0% / 2.1%) | – | – |
| Mental and Physical Effort | 41 / 78 (80.4% / 82.1%) | – | – |
| **NSW (Short / Long)** |  |  |  |
| Poor (7–27) | 0 / 3 (0.0% / 4.8%) | 7.168 | 0.067 |
| Moderate (28–36) | 9 / 10 (10.7% / 15.9%) | – | – |
| Good (37–43) | 43 / 35 (51.2% / 55.6%) | – | – |
| Excellent (44–49) | 32 / 15 (38.1% / 23.8%) | – | – |
| Poor/Moderate (7–36) | 9 / 13 (10.7% / 20.6%) | 2.784 | 0.095 |
| Good/Excellent (37–49) | 75 / 50 (89.3% / 79.4%) | – | – |
| Mental Effort | 14 / 11 (16.7% / 17.5%) | 0.124 | 0.940 |
| Physical Effort | 2 / 1 (2.4% / 1.6%) | – | – |
| Mental and Physical Effort | 68 / 51 (81.0% / 81.0%) | – | – |

**Supplementary Table S18. Anthropometric and metabolic parameters in day shift versus night shift workers (total cohort)**

| **Variable** | ***Category*** | **DSW N (%)** | **NSW N (%)** | **χ²** | **p-value** | **DSW Median (min–max)** | **NSW Median (min–max)** |
| --- | --- | --- | --- | --- | --- | --- | --- |
| **BMI (kg/m²)** | ***Underweight (<18.5)*** | 5 (5.2%) | 2 (1.4%) | 3.576 | 0.466 | 22 (17.9–38.1) | 23 (16.2–38.5) |
|  | ***Normal weight (18.5–24.9)*** | 63 (65.6%) | 96 (65.3%) |  |  |  |  |
|  | ***Overweight (25–29.9)*** | 23 (24.0%) | 40 (27.2%) |  |  |  |  |
|  | ***Obesity I (30–34.9)*** | 4 (4.2%) | 6 (4.1%) |  |  |  |  |
|  | ***Obesity II (35–39.9)*** | 1 (1.0%) | 3 (2.0%) |  |  |  |  |
|  | ***Obesity III (≥40)*** | 0 (0%) | 0 (0%) |  |  |  |  |
|  | ***Low (<25)*** | 68 (70.8%) | 98 (66.7%) | 0.466 | 0.495 |  |  |
|  | ***High (≥25)*** | 28 (29.2%) | 49 (33.3%) |  |  |  |  |
| **WHtR** | ***Low (≤0.5)*** | 48 (50.0%) | 57 (38.8%) | 3 | 0.084 | 0.5 (0.4–0.75) | 0.5196 (0.42–0.73) |
|  | ***High (>0.5)*** | 48 (50.0%) | 90 (61.2%) |  |  |  |  |
| **METS-IR** | ***Low (<30)*** | 21 (42.9%) | 55 (44.4%) | 0.352 | 0.839 | 32 (22.59–53.87) | 31 (21.02–66.32) |
|  | ***Moderate (30–45)*** | 25 (51.0%) | 64 (51.6%) |  |  |  |  |
|  | ***High (>45)*** | 3 (6.1%) | 5 (4.0%) |  |  |  |  |
|  | ***Low (≤45)*** | 46 (93.9%) | 119 (96.0%) | 0.348 | 0.555 |  |  |
|  | ***High (>45)*** | 3 (6.1%) | 5 (4.0%) |  |  |  |  |
| **TyG** | ***Low (<8.5)*** | 41 (82.0%) | 101 (81.5%) | 1 | 0.501 | 8 (6.93–10.01) | 8 (7.13–9.63) |
|  | ***Medium (8.5–8.8)*** | 3 (6.0%) | 13 (10.5%) |  |  |  |  |
|  | ***High (>8.8)*** | 6 (12.0%) | 10 (8.1%) |  |  |  |  |
|  | ***Low (≤8.8)*** | 44 (88.0%) | 114 (91.9%) | 0.661 | 0.416 |  |  |
|  | ***High (>8.8)*** | 6 (12.0%) | 10 (8.1%) |  |  |  |  |
| **WyG** | ***Low (≤8.19)*** | 52 (54.7%) | 62 (42.5%) | 3 | 0.062 | 8 (7.86–8.76) | 8 (7.82–8.9) |
|  | ***High (>8.19)*** | 43 (45.3%) | 84 (57.5%) |  |  |  |  |
| **TC/HDL** | ***Low (<3.5)*** | 34 (68.0%) | 79 (63.2%) | 2 | 0.464 | 3 (1.9–7.46) | 3 (1.86–6.94) |
|  | ***Medium (3.5–5)*** | 12 (24.0%) | 40 (32.0%) |  |  |  |  |
|  | ***High (>5)*** | 4 (8.0%) | 6 (4.8%) |  |  |  |  |
|  | ***Low (≤5)*** | 46 (92.0%) | 119 (95.2%) | 0.679 | 0.41 |  |  |
|  | ***High (>5)*** | 4 (8.0%) | 6 (4.8%) |  |  |  |  |
| **LDL/HDL** | ***Low (<2)*** | 26 (60.5%) | 65 (57.0%) | 0.357 | 0.837 | 1.8 (0.69–4.4) | 1.885 (0.65–4.35) |
|  | ***Medium (2–3)*** | 13 (30.2%) | 40 (35.1%) |  |  |  |  |
|  | ***High (>3)*** | 4 (9.3%) | 9 (7.9%) |  |  |  |  |
|  | ***Low (≤3)*** | 39 (90.7%) | 105 (92.1%) | 0.081 | 0.775 |  |  |
|  | ***High (>3)*** | 4 (9.3%) | 9 (7.9%) |  |  |  |  |
| **IRCV** | ***Low (<3%)*** | 43 (91.5%) | 111 (89.5%) | 0.148 | 0.7 | 0.58 (0.11–4.94) | 0.65 (0.07–8.64) |
|  | ***Intermediate (3–20%)*** | 4 (8.5%) | 13 (10.5%) |  |  |  |  |
|  | ***High (>20%)*** | 0 (0%) | 0 (0%) |  |  |  |  |

**Supplementary Table S19. Anthropometric and metabolic parameters in day shift versus night shift workers aged ≥30 years**

| **Variable** | **Category** | **DSW N (%)** | **NSW N (%)** | **χ²** | **p-value** | **DSW Median (min–max)** | **NSW Median (min–max)** |
| --- | --- | --- | --- | --- | --- | --- | --- |
| **BMI (kg/m²)** | ***Underweight (<18.5)*** | 1 (1.5%) | 2 (1.5%) | 2 | 0.784 | 23 (18.3–34.5) | 24 (16.2–38.5) |
|  | ***Normal weight (18.5–24.9)*** | 42 (64.6%) | 83 (62.9%) |  |  |  |  |
|  | ***Overweight (25–29.9)*** | 18 (27.7%) | 38 (28.8%) |  |  |  |  |
|  | ***Obesity I (30–34.9)*** | 4 (6.2%) | 6 (4.5%) |  |  |  |  |
|  | ***Obesity II (35–39.9)*** | 0 (0.0%) | 3 (2.3%) |  |  |  |  |
|  | ***Obesity III (≥40)*** | 0 (0.0%) | 0 (0.0%) |  |  |  |  |
|  | ***Low (<25)*** | 43 (66.2%) | 85 (64.4%) | 0.059 | 0.808 |  |  |
|  | ***High (≥25)*** | 22 (33.8%) | 47 (35.6%) |  |  |  |  |
| **WHtR** | ***Low (≤0.5)*** | 30 (46.2%) | 47 (35.6%) | 2 | 0.154 | 0.5066 (0.4–0.71) | 0.524 (0.42–0.73) |
|  | ***High (>0.5)*** | 35 (53.8%) | 85 (64.4%) |  |  |  |  |
| **METS-IR** | ***Low (<30)*** | 16 (38.1%) | 47 (41.2%) | 0.53 | 0.767 | 32.6842 (22.59–53.87) | 31.4983 (21.02–66.32) |
|  | ***Moderate (30–45)*** | 23 (54.8%) | 62 (54.4%) |  |  |  |  |
|  | ***High (>45)*** | 3 (7.1%) | 5 (4.4%) |  |  |  |  |
|  | ***Low (≤45)*** | 39 (92.9%) | 109 (95.6%) | 0.479 | 0.489 |  |  |
|  | ***High (>45)*** | 3 (7.1%) | 5 (4.4%) |  |  |  |  |
| **TyG** | ***Low (<8.5)*** | 34 (81.0%) | 91 (79.8%) | 2.323 | 0.313 | 8.1395 (6.93–10.01) | 8.0765 (7.13–9.63) |
|  | ***Medium (8.5–8.8)*** | 2 (4.8%) | 13 (11.4%) |  |  |  |  |
|  | ***High (>8.8)*** | 6 (14.3%) | 10 (8.8%) |  |  |  |  |
|  | ***Low (≤8.8)*** | 36 (85.7%) | 104 (91.2%) | 1.014 | 0.314 |  |  |
|  | ***High (>8.8)*** | 6 (14.3%) | 10 (8.8%) |  |  |  |  |
| **WyG** | ***Low (≤8.19)*** | 29 (45.3%) | 51 (38.9%) | 0.724 | 0.395 | 8.2186 (7.92–8.76) | 8.2477 (7.82–8.9) |
|  | ***High (>8.19)*** | 35 (54.7%) | 80 (61.1%) |  |  |  |  |
| **TC/HDL** | ***Low (<3.5)*** | 26 (61.9%) | 71 (61.7%) | 2 | 0.426 | 3.09 (1.9–7.46) | 3.19 (1.87–6.94) |
|  | ***Medium (3.5–5)*** | 12 (28.6%) | 39 (33.9%) |  |  |  |  |
|  | ***High (>5)*** | 4 (9.5%) | 5 (4.3%) |  |  |  |  |
|  | ***Low (≤5)*** | 38 (90.5%) | 110 (95.7%) | 2 | 0.217 |  |  |
|  | ***High (>5)*** | 4 (9.5%) | 5 (4.3%) |  |  |  |  |
| **LDL/HDL** | ***Low (<2)*** | 22 (56.4%) | 58 (55.2%) | 0.359 | 0.836 | 1.84 (0.69–4.4) | 1.92 (0.65–4.35) |
|  | ***Medium (2–3)*** | 13 (33.3%) | 39 (37.1%) |  |  |  |  |
|  | ***High (>3)*** | 4 (10.3%) | 8 (7.6%) |  |  |  |  |
|  | ***Low (≤3)*** | 35 (89.7%) | 97 (92.4%) | 0.259 | 0.611 |  |  |
|  | ***High (>3)*** | 4 (10.3%) | 8 (7.6%) |  |  |  |  |
| **IRCV** | ***Low (<3%)*** | 35 (89.7%) | 101 (88.6%) | 0.039 | 0.844 | 0.7 (0.11–4.94) | 0.75 (0.08–8.64) |
|  | ***Intermediate (3–20%)*** | 4 (10.3%) | 13 (11.4%) |  |  |  |  |
|  | ***High (>20%)*** | 0 (0%) | 0 (0%) |  |  |  |  |

**Supplementary Table S20. Anthropometric and metabolic parameters among night shift workers by cumulative exposure (<500 vs. ≥500 lifetime night shifts)**

| **Variable** | **Category** | **Low N (%)** | **High N (%)** | **χ²** | **p-value** | **Low Median (min–max)** | **High Median (min–max)** |
| --- | --- | --- | --- | --- | --- | --- | --- |
| **BMI (kg/m²)** | ***Underweight (<18.5)*** | 1 (2.0%) | 1 (1.1%) | 4.627 | 0.328 | 23.05 (16.2–30.8) | 24 (17.2–38.5) |
|  | ***Normal weight (18.5–24.9)*** | 38 (74.5%) | 57 (60.0%) |  |  |  |  |
|  | ***Overweight (25–29.9)*** | 10 (19.6%) | 30 (31.6%) |  |  |  |  |
|  | ***Obesity I (30–34.9)*** | 2 (3.9%) | 4 (4.2%) |  |  |  |  |
|  | ***Obesity II (35–39.9)*** | 0 (0.0%) | 3 (3.2%) |  |  |  |  |
|  | ***Obesity III (≥40)*** | 0 (0.0%) | 0 (0.0%) |  |  |  |  |
|  | ***Low (<25)*** | 39 (76.5%) | 58 (61.1%) | 3.538 | 0.06 |  |  |
|  | ***High (≥25)*** | 12 (23.5%) | 37 (38.9%) |  |  |  |  |
| **WHtR** | ***Low (≤0.5)*** | 25 (49.0%) | 32 (33.7%) | 3.279 | 0.07 | 0.5118 (0.42–0.65) | 0.5258 (0.43–0.73) |
|  | ***High (>0.5)*** | 26 (51.0%) | 63 (66.3%) |  |  |  |  |
| **METS-IR** | ***Low (<30)*** | 17 (50.0%) | 38 (42.2%) | 2.249 | 0.325 | 30 (22.04–43.97) | 31 (21.02–66.32) |
|  | ***Moderate (30–45)*** | 17 (50.0%) | 47 (52.2%) |  |  |  |  |
|  | ***High (>45)*** | 0 (0.0%) | 5 (5.6%) |  |  |  |  |
|  | ***Low (≤45)*** | 34 (100%) | 85 (94.4%) | 1.968 | 0.161 |  |  |
|  | ***High (>45)*** | 0 (0.0%) | 5 (5.6%) |  |  |  |  |
| **TyG** | ***Low (<8.5)*** | 32 (94.1%) | 69 (76.7%) | 5.647 | 0.059 | 8 (7.28–8.75) | 8 (7.13–9.63) |
|  | ***Medium (8.5–8.8)*** | 2 (5.9%) | 11 (12.2%) |  |  |  |  |
|  | ***High (>8.8)*** | 0 (0.0%) | 10 (11.1%) |  |  |  |  |
|  | ***Low (≤8.8)*** | 34 (100%) | 80 (88.9%) | 4.109 | **0.043** |  |  |
|  | ***High (>8.8)*** | 0 (0.0%) | 10 (11.1%) |  |  |  |  |
| **WyG** | ***Low (≤8.19)*** | 28 (54.9%) | 34 (36.2%) | 4.74 | **0.029** | 81.648 (7.85–8.64) | 82.567 (7.82–8.9) |
|  | ***High (>8.19)*** | 23 (45.1%) | 60 (63.8%) |  |  |  |  |
| **TC/HDL** | ***Low (<3.5)*** | 23 (67.6%) | 56 (61.5%) | 0.706 | 0.702 | 3.135 (1.86–5.55) | 3.22 (1.87–6.94) |
|  | ***Medium (3.5–5)*** | 9 (26.5%) | 31 (34.1%) |  |  |  |  |
|  | ***High (>5)*** | 2 (5.9%) | 4 (4.4%) |  |  |  |  |
|  | ***Low (≤5)*** | 32 (94.1%) | 87 (95.6%) | 0.12 | 0.729 |  |  |
|  | ***High (>5)*** | 2 (5.9%) | 4 (4.4%) |  |  |  |  |
| **LDL/HDL** | ***Low (<2)*** | 23 (69.7%) | 42 (51.9%) | 3.064 | 0.216 | 1.64 (0.75–3.7) | 1.98 (0.65–4.35) |
|  | ***Medium (2–3)*** | 8 (24.2%) | 32 (39.5%) |  |  |  |  |
|  | ***High (>3)*** | 2 (6.1%) | 7 (8.6%) |  |  |  |  |
|  | ***Low (≤3)*** | 31 (93.9%) | 74 (91.4%) | 0.215 | 0.643 |  |  |
|  | ***High (>3)*** | 2 (6.1%) | 7 (8.6%) |  |  |  |  |
| **IRCV** | ***Low (<3%)*** | 32 (94.1%) | 79 (87.8%) | 1.057 | 0.304 | 0.315 (0.07–4.69) | 0.795 (0.08–8.64) |
|  | ***Intermediate (3–20%)*** | 2 (5.9%) | 11 (12.2%) |  |  |  |  |
|  | ***High (>20%)*** | 0 (0%) | 0 (0%) |  |  |  |  |

**Supplementary Table S21. Anthropometric and metabolic parameters among night shift workers by duration of exposure (1–10 vs. 11–35 years)**

| **Variable** | **Category** | **Short N (%)** | **Long N (%)** | **χ²** | **p-value** | **Short Median (min–max)** | **Long Median (min–max)** |
| --- | --- | --- | --- | --- | --- | --- | --- |
| **BMI (kg/m²)** | ***Underweight (<18.5)*** | 1 (1.2%) | 1 (1.6%) | 7.29 | 0.121 | 22.2 (16.2–34.9) | 24.01 (17.2–38.5) |
|  | ***Normal weight (18.5–24.9)*** | 61 (72.6%) | 35 (55.6%) |  |  |  |  |
|  | ***Overweight (25–29.9)*** | 19 (22.6%) | 21 (33.3%) |  |  |  |  |
|  | ***Obesity I (30–34.9)*** | 3 (3.6%) | 3 (4.8%) |  |  |  |  |
|  | ***Obesity II (35–39.9)*** | 0 (0.0%) | 3 (4.8%) |  |  |  |  |
|  | ***Obesity III (≥40)*** | 0 (0.0%) | 0 (0.0%) |  |  |  |  |
|  | ***Low (<25)*** | 62 (73.8%) | 36 (57.1%) | 4.5 | **0.034** |  |  |
|  | ***High (≥25)*** | 22 (26.2%) | 27 (42.9%) |  |  |  |  |
| **WHtR** | ***Low (≤0.5)*** | 39 (46.4%) | 18 (28.6%) | 4.836 | **0.028** | 0.5126 (0.42–0.73) | 0.5268 (0.43–0.73) |
|  | ***High (>0.5)*** | 45 (53.6%) | 45 (71.4%) |  |  |  |  |
| **METS-IR** | ***Low (<30)*** | 31 (49.2%) | 24 (39.3%) | 2.722 | 0.256 | 30.779 (22.04–49.97) | 32.048 (21.02–66.32) |
|  | ***Moderate (30–45)*** | 31 (49.2%) | 33 (54.1%) |  |  |  |  |
|  | ***High (>45)*** | 1 (1.6%) | 4 (6.6%) |  |  |  |  |
|  | ***Low (≤45)*** | 62 (98.4%) | 57 (93.4%) | 1.978 | 0.16 |  |  |
|  | ***High (>45)*** | 1 (1.6%) | 4 (6.6%) |  |  |  |  |
| **TyG** | ***Low (<8.5)*** | 56 (88.9%) | 45 (73.8%) | 4.69 | 0.096 | 7.954 (7.13–9.13) | 8.191 (7.45–9.63) |
|  | ***Medium (8.5–8.8)*** | 4 (6.3%) | 9 (14.8%) |  |  |  |  |
|  | ***High (>8.8)*** | 3 (4.8%) | 7 (11.5%) |  |  |  |  |
|  | ***Low (≤8.8)*** | 60 (95.2%) | 54 (88.5%) | 1.884 | 0.17 |  |  |
|  | ***High (>8.8)*** | 3 (4.8%) | 7 (11.5%) |  |  |  |  |
| **WyG** | ***Low (≤8.19)*** | 40 (47.6%) | 22 (35.5%) | 2.15 | 0.143 | 82.058 (7.83–8.64) | 82.745 (7.82–8.9) |
|  | ***High (>8.19)*** | 44 (52.4%) | 40 (64.5%) |  |  |  |  |
| **TC/HDL** | ***Low (<3.5)*** | 45 (70.3%) | 34 (55.7%) | 3.028 | 0.22 | 3.135 (1.86–5.55) | 3.23 (1.87–6.94) |
|  | ***Medium (3.5–5)*** | 17 (26.6%) | 23 (37.7%) |  |  |  |  |
|  | ***High (>5)*** | 2 (3.1%) | 4 (6.6%) |  |  |  |  |
|  | ***Low (≤5)*** | 62 (96.9%) | 57 (93.4%) | 0.805 | 0.37 |  |  |
|  | ***High (>5)*** | 2 (3.1%) | 4 (6.6%) |  |  |  |  |
| **LDL/HDL** | ***Low (<2)*** | 38 (64.4%) | 27 (49.1%) | 3.125 | 0.21 | 1.7 (0.75–3.7) | 2.02 (0.65––) |
|  | ***Medium (2–3)*** | 18 (30.5%) | 22 (40.0%) |  |  |  |  |
|  | ***High (>3)*** | 3 (5.1%) | 6 (10.9%) |  |  |  |  |
|  | ***Low (≤3)*** | 56 (94.9%) | 49 (89.1%) | 1.328 | 0.249 |  |  |
|  | ***High (>3)*** | 3 (5.1%) | 6 (10.9%) |  |  |  |  |
| **IRCV** | ***Low (<3%)*** | 62 (96.9%) | 49 (81.7%) | 7.632 | **0.006** | 0.37 (0.07–4.69) | 1.095 (0.14–8.64) |
|  | ***Intermediate (3–20%)*** | 2 (3.1%) | 11 (18.3%) |  |  |  |  |
|  | ***High (>20%)*** | 0 (0%) | 0 (0%) |  |  |  |  |

**Supplementary Table S22. Spearman correlation coefficients (ρ) and p-values, and Mantel test statistics (r) with p-values for neurobehavioral, anthropometric, metabolic, and cardiovascular indices by population and night-shift exposure.** Population groups: ALL = all participants; DSW = day shift workers; NSW = night shift workers. Within NSW, participants were further classified by cumulative exposure and duration: LOW (<500 night shifts) and HIGH (≥500 night shifts) indicate total lifetime number of night shifts; SHORT (1–10 years) and LONG (11–35 years) indicate duration of night shift work.

|  | rValue | | | | | | | | | | | | | | | | | | | | | |
| --- | --- | --- | --- | --- | --- | --- | --- | --- | --- | --- | --- | --- | --- | --- | --- | --- | --- | --- | --- | --- | --- | --- |
| **ALL** | AGE | NSW | NSW_CUM | NSW_YRS | MEDI_LITE | IPAQ | PSQI | WHO | WAI | BMI | WHTR | METSIR | TYG | WYG | TC/HDL | LDL/HDL | IRCV | SMOKE | ALCOHOL | PHYS_ACT | SLEEP_HR | SLEEP_SAT |
| AGE | 1,000 | 0,228 | 0,410 | 0,404 | 0,012 | 0,072 | 0,377 | -0,204 | -0,387 | 0,284 | 0,283 | 0,274 | 0,369 | 0,366 | 0,267 | 0,287 | 0,838 | 0,070 | -0,170 | -0,229 | -0,380 | 0,318 |
| NSW | 0,228 | 1,000 | 0,875 | 0,874 | 0,044 | -0,045 | 0,226 | 0,031 | -0,083 | 0,144 | 0,126 | 0,018 | -0,022 | 0,132 | 0,030 | 0,000 | 0,036 | 0,167 | 0,014 | 0,008 | -0,161 | 0,212 |
| NSW_CUM | 0,410 | 0,875 | 1,000 | 0,991 | 0,043 | -0,064 | 0,322 | -0,059 | -0,177 | 0,197 | 0,186 | 0,127 | 0,114 | 0,204 | 0,099 | 0,120 | 0,291 | 0,178 | -0,049 | -0,049 | -0,257 | 0,302 |
| NSW_YRS | 0,404 | 0,874 | 0,991 | 1,000 | 0,033 | -0,052 | 0,305 | -0,055 | -0,164 | 0,190 | 0,178 | 0,114 | 0,095 | 0,200 | 0,073 | 0,093 | 0,281 | 0,188 | -0,040 | -0,042 | -0,246 | 0,291 |
| MEDI_LITE | 0,012 | 0,044 | 0,043 | 0,033 | 1,000 | 0,000 | -0,055 | 0,062 | -0,036 | -0,112 | -0,099 | -0,080 | -0,052 | -0,063 | -0,012 | 0,011 | -0,082 | -0,122 | -0,017 | 0,142 | -0,078 | 0,071 |
| IPAQ | 0,072 | -0,045 | -0,064 | -0,052 | 0,000 | 1,000 | 0,055 | 0,005 | -0,092 | -0,095 | -0,080 | -0,083 | -0,092 | -0,091 | -0,113 | -0,069 | 0,069 | 0,076 | 0,143 | 0,252 | -0,072 | -0,090 |
| PSQI | 0,377 | 0,226 | 0,322 | 0,305 | -0,055 | 0,055 | 1,000 | -0,433 | -0,494 | 0,148 | 0,167 | 0,110 | 0,079 | 0,160 | 0,118 | 0,165 | 0,397 | 0,052 | 0,035 | -0,086 | -0,622 | 0,667 |
| WHO | -0,204 | 0,031 | -0,059 | -0,055 | 0,062 | 0,005 | -0,433 | 1,000 | 0,529 | -0,073 | -0,095 | -0,111 | -0,152 | -0,168 | -0,107 | -0,178 | -0,285 | 0,000 | -0,109 | 0,080 | 0,254 | -0,431 |
| WAI | -0,387 | -0,083 | -0,177 | -0,164 | -0,036 | -0,092 | -0,494 | 0,529 | 1,000 | -0,202 | -0,220 | -0,205 | -0,122 | -0,217 | -0,117 | -0,198 | -0,402 | -0,062 | -0,065 | 0,112 | 0,370 | -0,401 |
| BMI | 0,284 | 0,144 | 0,197 | 0,190 | -0,112 | -0,095 | 0,148 | -0,073 | -0,202 | 1,000 | 0,947 | 0,947 | 0,317 | 0,695 | 0,369 | 0,386 | 0,294 | -0,075 | -0,112 | -0,265 | -0,193 | 0,132 |
| WHTR | 0,283 | 0,126 | 0,186 | 0,178 | -0,099 | -0,080 | 0,167 | -0,095 | -0,220 | 0,947 | 1,000 | 0,899 | 0,320 | 0,666 | 0,376 | 0,388 | 0,303 | -0,087 | -0,106 | -0,259 | -0,212 | 0,147 |
| METSIR | 0,274 | 0,018 | 0,127 | 0,114 | -0,080 | -0,083 | 0,110 | -0,111 | -0,205 | 0,947 | 0,899 | 1,000 | 0,468 | 0,722 | 0,568 | 0,552 | 0,389 | -0,096 | -0,117 | -0,245 | -0,175 | 0,104 |
| TYG | 0,369 | -0,022 | 0,114 | 0,095 | -0,052 | -0,092 | 0,079 | -0,152 | -0,122 | 0,317 | 0,320 | 0,468 | 1,000 | 0,500 | 0,493 | 0,407 | 0,396 | -0,091 | -0,088 | -0,160 | -0,149 | 0,169 |
| WYG | 0,366 | 0,132 | 0,204 | 0,200 | -0,063 | -0,091 | 0,160 | -0,168 | -0,217 | 0,695 | 0,666 | 0,722 | 0,500 | 1,000 | 0,338 | 0,332 | 0,368 | -0,079 | -0,064 | -0,193 | -0,256 | 0,209 |
| TC/HDL | 0,267 | 0,030 | 0,099 | 0,073 | -0,012 | -0,113 | 0,118 | -0,107 | -0,117 | 0,369 | 0,376 | 0,568 | 0,493 | 0,338 | 1,000 | 0,974 | 0,437 | 0,025 | -0,070 | -0,217 | -0,054 | 0,143 |
| LDL/HDL | 0,287 | 0,000 | 0,120 | 0,093 | 0,011 | -0,069 | 0,165 | -0,178 | -0,198 | 0,386 | 0,388 | 0,552 | 0,407 | 0,332 | 0,974 | 1,000 | 0,456 | 0,071 | -0,079 | -0,210 | -0,079 | 0,162 |
| IRCV | 0,838 | 0,036 | 0,291 | 0,281 | -0,082 | 0,069 | 0,397 | -0,285 | -0,402 | 0,294 | 0,303 | 0,389 | 0,396 | 0,368 | 0,437 | 0,456 | 1,000 | 0,271 | -0,075 | -0,184 | -0,353 | 0,342 |
| SMOKE | 0,070 | 0,167 | 0,178 | 0,188 | -0,122 | 0,076 | 0,052 | 0,000 | -0,062 | -0,075 | -0,087 | -0,096 | -0,091 | -0,079 | 0,025 | 0,071 | 0,271 | 1,000 | 0,083 | -0,096 | 0,000 | 0,047 |
| ALCOHOL | -0,170 | 0,014 | -0,049 | -0,040 | -0,017 | 0,143 | 0,035 | -0,109 | -0,065 | -0,112 | -0,106 | -0,117 | -0,088 | -0,064 | -0,070 | -0,079 | -0,075 | 0,083 | 1,000 | 0,180 | 0,069 | -0,017 |
| PHYS_ACT | -0,229 | 0,008 | -0,049 | -0,042 | 0,142 | 0,252 | -0,086 | 0,080 | 0,112 | -0,265 | -0,259 | -0,245 | -0,160 | -0,193 | -0,217 | -0,210 | -0,184 | -0,096 | 0,180 | 1,000 | 0,127 | -0,114 |
| SLEEP_HR | -0,380 | -0,161 | -0,257 | -0,246 | -0,078 | -0,072 | -0,622 | 0,254 | 0,370 | -0,193 | -0,212 | -0,175 | -0,149 | -0,256 | -0,054 | -0,079 | -0,353 | 0,000 | 0,069 | 0,127 | 1,000 | -0,596 |
| SLEEP_SAT | 0,318 | 0,212 | 0,302 | 0,291 | 0,071 | -0,090 | 0,667 | -0,431 | -0,401 | 0,132 | 0,147 | 0,104 | 0,169 | 0,209 | 0,143 | 0,162 | 0,342 | 0,047 | -0,017 | -0,114 | -0,596 | 1,000 |

|  | pValue | | | | | | | | | | | | | | | | | | | | | |
| --- | --- | --- | --- | --- | --- | --- | --- | --- | --- | --- | --- | --- | --- | --- | --- | --- | --- | --- | --- | --- | --- | --- |
| **ALL** | AGE | NSW | NSW_CUM | NSW_YRS | MEDI_LITE | IPAQ | PSQI | WHO | WAI | BMI | WHTR | METSIR | TYG | WYG | TC/HDL | LDL/HDL | IRCV | SMOKE | ALCOHOL | PHYS_ACT | SLEEP_HR | SLEEP_SAT |
| AGE | 1,000 | 0,000 | 0,000 | 0,000 | 0,858 | 0,261 | 0,000 | 0,001 | 0,000 | 0,000 | 0,000 | 0,000 | 0,000 | 0,000 | 0,000 | 0,000 | 0,000 | 0,274 | 0,008 | 0,000 | 0,000 | 0,000 |
| NSW | 0,000 | 1,000 | 0,000 | 0,000 | 0,497 | 0,485 | 0,000 | 0,632 | 0,199 | 0,024 | 0,049 | 0,814 | 0,777 | 0,041 | 0,692 | 0,998 | 0,638 | 0,009 | 0,828 | 0,899 | 0,012 | 0,001 |
| NSW_CUM | 0,000 | 0,000 | 1,000 | 0,000 | 0,510 | 0,320 | 0,000 | 0,363 | 0,006 | 0,002 | 0,004 | 0,096 | 0,135 | 0,001 | 0,192 | 0,134 | 0,000 | 0,005 | 0,447 | 0,443 | 0,000 | 0,000 |
| NSW_YRS | 0,000 | 0,000 | 0,000 | 1,000 | 0,605 | 0,418 | 0,000 | 0,391 | 0,010 | 0,003 | 0,005 | 0,135 | 0,214 | 0,002 | 0,337 | 0,248 | 0,000 | 0,003 | 0,533 | 0,512 | 0,000 | 0,000 |
| MEDI_LITE | 0,858 | 0,497 | 0,510 | 0,605 | 1,000 | 0,997 | 0,397 | 0,334 | 0,581 | 0,080 | 0,123 | 0,296 | 0,494 | 0,329 | 0,878 | 0,891 | 0,286 | 0,058 | 0,792 | 0,027 | 0,227 | 0,269 |
| IPAQ | 0,261 | 0,485 | 0,320 | 0,418 | 0,997 | 1,000 | 0,393 | 0,934 | 0,152 | 0,141 | 0,214 | 0,278 | 0,227 | 0,158 | 0,136 | 0,392 | 0,373 | 0,237 | 0,026 | 0,000 | 0,260 | 0,161 |
| PSQI | 0,000 | 0,000 | 0,000 | 0,000 | 0,397 | 0,393 | 1,000 | 0,000 | 0,000 | 0,021 | 0,009 | 0,149 | 0,303 | 0,013 | 0,119 | 0,039 | 0,000 | 0,420 | 0,587 | 0,180 | 0,000 | 0,000 |
| WHO | 0,001 | 0,632 | 0,363 | 0,391 | 0,334 | 0,934 | 0,000 | 1,000 | 0,000 | 0,256 | 0,141 | 0,147 | 0,045 | 0,009 | 0,160 | 0,026 | 0,000 | 1,000 | 0,090 | 0,213 | 0,000 | 0,000 |
| WAI | 0,000 | 0,199 | 0,006 | 0,010 | 0,581 | 0,152 | 0,000 | 0,000 | 1,000 | 0,002 | 0,001 | 0,007 | 0,108 | 0,001 | 0,123 | 0,013 | 0,000 | 0,333 | 0,310 | 0,082 | 0,000 | 0,000 |
| BMI | 0,000 | 0,024 | 0,002 | 0,003 | 0,080 | 0,141 | 0,021 | 0,256 | 0,002 | 1,000 | 0,000 | 0,000 | 0,000 | 0,000 | 0,000 | 0,000 | 0,000 | 0,242 | 0,083 | 0,000 | 0,002 | 0,040 |
| WHTR | 0,000 | 0,049 | 0,004 | 0,005 | 0,123 | 0,214 | 0,009 | 0,141 | 0,001 | 0,000 | 1,000 | 0,000 | 0,000 | 0,000 | 0,000 | 0,000 | 0,000 | 0,178 | 0,099 | 0,000 | 0,001 | 0,022 |
| METSIR | 0,000 | 0,814 | 0,096 | 0,135 | 0,296 | 0,278 | 0,149 | 0,147 | 0,007 | 0,000 | 0,000 | 1,000 | 0,000 | 0,000 | 0,000 | 0,000 | 0,000 | 0,210 | 0,125 | 0,001 | 0,021 | 0,172 |
| TYG | 0,000 | 0,777 | 0,135 | 0,214 | 0,494 | 0,227 | 0,303 | 0,045 | 0,108 | 0,000 | 0,000 | 0,000 | 1,000 | 0,000 | 0,000 | 0,000 | 0,000 | 0,234 | 0,250 | 0,034 | 0,049 | 0,025 |
| WYG | 0,000 | 0,041 | 0,001 | 0,002 | 0,329 | 0,158 | 0,013 | 0,009 | 0,001 | 0,000 | 0,000 | 0,000 | 0,000 | 1,000 | 0,000 | 0,000 | 0,000 | 0,224 | 0,319 | 0,003 | 0,000 | 0,001 |
| TC/HDL | 0,000 | 0,692 | 0,192 | 0,337 | 0,878 | 0,136 | 0,119 | 0,160 | 0,123 | 0,000 | 0,000 | 0,000 | 0,000 | 0,000 | 1,000 | 0,000 | 0,000 | 0,743 | 0,359 | 0,004 | 0,476 | 0,058 |
| LDL/HDL | 0,000 | 0,998 | 0,134 | 0,248 | 0,891 | 0,392 | 0,039 | 0,026 | 0,013 | 0,000 | 0,000 | 0,000 | 0,000 | 0,000 | 0,000 | 1,000 | 0,000 | 0,376 | 0,325 | 0,008 | 0,324 | 0,042 |
| IRCV | 0,000 | 0,638 | 0,000 | 0,000 | 0,286 | 0,373 | 0,000 | 0,000 | 0,000 | 0,000 | 0,000 | 0,000 | 0,000 | 0,000 | 0,000 | 0,000 | 1,000 | 0,000 | 0,331 | 0,016 | 0,000 | 0,000 |
| SMOKE | 0,274 | 0,009 | 0,005 | 0,003 | 0,058 | 0,237 | 0,420 | 1,000 | 0,333 | 0,242 | 0,178 | 0,210 | 0,234 | 0,224 | 0,743 | 0,376 | 0,000 | 1,000 | 0,198 | 0,134 | 1,000 | 0,467 |
| ALCOHOL | 0,008 | 0,828 | 0,447 | 0,533 | 0,792 | 0,026 | 0,587 | 0,090 | 0,310 | 0,083 | 0,099 | 0,125 | 0,250 | 0,319 | 0,359 | 0,325 | 0,331 | 0,198 | 1,000 | 0,005 | 0,285 | 0,794 |
| PHYS_ACT | 0,000 | 0,899 | 0,443 | 0,512 | 0,027 | 0,000 | 0,180 | 0,213 | 0,082 | 0,000 | 0,000 | 0,001 | 0,034 | 0,003 | 0,004 | 0,008 | 0,016 | 0,134 | 0,005 | 1,000 | 0,048 | 0,077 |
| SLEEP_HR | 0,000 | 0,012 | 0,000 | 0,000 | 0,227 | 0,260 | 0,000 | 0,000 | 0,000 | 0,002 | 0,001 | 0,021 | 0,049 | 0,000 | 0,476 | 0,324 | 0,000 | 1,000 | 0,285 | 0,048 | 1,000 | 0,000 |
| SLEEP_SAT | 0,000 | 0,001 | 0,000 | 0,000 | 0,269 | 0,161 | 0,000 | 0,000 | 0,000 | 0,040 | 0,022 | 0,172 | 0,025 | 0,001 | 0,058 | 0,042 | 0,000 | 0,467 | 0,794 | 0,077 | 0,000 | 1,000 |

|  | Mantel's | |
| --- | --- | --- |
| **ALL** | r | p |
| AGE | 0,039 | 0,081 |
| NSW | 0,214 | 0,001 |
| NSW_CUM | 0,295 | 0,001 |
| NSW_YRS | 0,301 | 0,001 |
| MEDI_LITE | -0,034 | 0,855 |
| IPAQ | 0,536 | 0,001 |
| PSQI | 0,087 | 0,005 |
| WHO | 0,041 | 0,089 |
| WAI | 0,119 | 0,004 |
| BMI | 0,018 | 0,276 |
| WHTR | -0,004 | 0,531 |
| METSIR | -0,003 | 0,508 |
| TYG | -0,045 | 0,926 |
| WYG | -0,003 | 0,522 |
| TC/HDL | -0,036 | 0,839 |
| LDL/HDL | -0,041 | 0,892 |
| IRCV | -0,036 | 0,825 |
| SMOKE | 0,011 | 0,312 |
| ALCOHOL | 0,035 | 0,061 |
| PHYS_ACT | -0,005 | 0,579 |
| SLEEP_HR | 0,056 | 0,038 |
| SLEEP_SAT | 0,029 | 0,151 |

|  | rValue | | | | | | | | | | | | | | | | | | |
| --- | --- | --- | --- | --- | --- | --- | --- | --- | --- | --- | --- | --- | --- | --- | --- | --- | --- | --- | --- |
| **DSW** | AGE | MEDI_LITE | IPAQ | PSQI | WHO | WAI | BMI | WHTR | METSIR | TYG | WYG | TC/HDL | LDL/HDL | IRCV | SMOKE | ALCOHOL | PHYS_ACT | SLEEP_HR | SLEEP_SAT |
| AGE | 1,000 | 0,009 | 0,222 | 0,272 | -0,191 | -0,388 | 0,294 | 0,247 | 0,373 | 0,494 | 0,433 | 0,492 | 0,419 | 0,889 | 0,131 | -0,218 | -0,266 | -0,278 | 0,200 |
| MEDI_LITE | 0,009 | 1,000 | -0,030 | -0,244 | 0,004 | 0,046 | -0,140 | -0,155 | -0,219 | -0,211 | -0,153 | -0,086 | -0,059 | -0,218 | -0,160 | -0,076 | 0,134 | 0,096 | -0,071 |
| IPAQ | 0,222 | -0,030 | 1,000 | 0,194 | -0,087 | -0,128 | -0,008 | -0,021 | 0,083 | 0,236 | 0,060 | 0,034 | 0,074 | 0,258 | -0,075 | 0,040 | 0,264 | -0,151 | -0,012 |
| PSQI | 0,272 | -0,244 | 0,194 | 1,000 | -0,378 | -0,384 | 0,167 | 0,207 | 0,209 | 0,164 | 0,156 | 0,246 | 0,198 | 0,472 | 0,085 | 0,048 | -0,166 | -0,674 | 0,629 |
| WHO | -0,191 | 0,004 | -0,087 | -0,378 | 1,000 | 0,475 | -0,145 | -0,149 | -0,284 | -0,210 | -0,245 | -0,241 | -0,262 | -0,283 | -0,062 | -0,096 | -0,019 | 0,297 | -0,492 |
| WAI | -0,388 | 0,046 | -0,128 | -0,384 | 0,475 | 1,000 | -0,351 | -0,367 | -0,543 | -0,317 | -0,397 | -0,439 | -0,427 | -0,373 | -0,018 | -0,064 | 0,183 | 0,397 | -0,350 |
| BMI | 0,294 | -0,140 | -0,008 | 0,167 | -0,145 | -0,351 | 1,000 | 0,941 | 0,956 | 0,243 | 0,719 | 0,306 | 0,277 | 0,378 | 0,025 | -0,023 | -0,330 | -0,263 | 0,242 |
| WHTR | 0,247 | -0,155 | -0,021 | 0,207 | -0,149 | -0,367 | 0,941 | 1,000 | 0,912 | 0,234 | 0,670 | 0,365 | 0,277 | 0,406 | -0,001 | 0,017 | -0,335 | -0,290 | 0,267 |
| METSIR | 0,373 | -0,219 | 0,083 | 0,209 | -0,284 | -0,543 | 0,956 | 0,912 | 1,000 | 0,376 | 0,694 | 0,507 | 0,459 | 0,487 | -0,071 | -0,035 | -0,330 | -0,254 | 0,275 |
| TYG | 0,494 | -0,211 | 0,236 | 0,164 | -0,210 | -0,317 | 0,243 | 0,234 | 0,376 | 1,000 | 0,454 | 0,596 | 0,498 | 0,480 | 0,037 | 0,060 | -0,113 | -0,183 | 0,231 |
| WYG | 0,433 | -0,153 | 0,060 | 0,156 | -0,245 | -0,397 | 0,719 | 0,670 | 0,694 | 0,454 | 1,000 | 0,205 | 0,140 | 0,438 | 0,025 | 0,008 | -0,213 | -0,278 | 0,283 |
| TC/HDL | 0,492 | -0,086 | 0,034 | 0,246 | -0,241 | -0,439 | 0,306 | 0,365 | 0,507 | 0,596 | 0,205 | 1,000 | 0,974 | 0,590 | 0,035 | 0,060 | -0,396 | -0,181 | 0,243 |
| LDL/HDL | 0,419 | -0,059 | 0,074 | 0,198 | -0,262 | -0,427 | 0,277 | 0,277 | 0,459 | 0,498 | 0,140 | 0,974 | 1,000 | 0,580 | 0,108 | 0,019 | -0,271 | -0,097 | 0,190 |
| IRCV | 0,889 | -0,218 | 0,258 | 0,472 | -0,283 | -0,373 | 0,378 | 0,406 | 0,487 | 0,480 | 0,438 | 0,590 | 0,580 | 1,000 | 0,337 | -0,041 | -0,269 | -0,270 | 0,243 |
| SMOKE | 0,131 | -0,160 | -0,075 | 0,085 | -0,062 | -0,018 | 0,025 | -0,001 | -0,071 | 0,037 | 0,025 | 0,035 | 0,108 | 0,337 | 1,000 | 0,018 | -0,160 | 0,052 | 0,008 |
| ALCOHOL | -0,218 | -0,076 | 0,040 | 0,048 | -0,096 | -0,064 | -0,023 | 0,017 | -0,035 | 0,060 | 0,008 | 0,060 | 0,019 | -0,041 | 0,018 | 1,000 | 0,128 | -0,045 | 0,068 |
| PHYS_ACT | -0,266 | 0,134 | 0,264 | -0,166 | -0,019 | 0,183 | -0,330 | -0,335 | -0,330 | -0,113 | -0,213 | -0,396 | -0,271 | -0,269 | -0,160 | 0,128 | 1,000 | 0,226 | -0,203 |
| SLEEP_HR | -0,278 | 0,096 | -0,151 | -0,674 | 0,297 | 0,397 | -0,263 | -0,290 | -0,254 | -0,183 | -0,278 | -0,181 | -0,097 | -0,270 | 0,052 | -0,045 | 0,226 | 1,000 | -0,655 |
| SLEEP_SAT | 0,200 | -0,071 | -0,012 | 0,629 | -0,492 | -0,350 | 0,242 | 0,267 | 0,275 | 0,231 | 0,283 | 0,243 | 0,190 | 0,243 | 0,008 | 0,068 | -0,203 | -0,655 | 1,000 |

|  | pValue | | | | | | | | | | | | | | | | | | |
| --- | --- | --- | --- | --- | --- | --- | --- | --- | --- | --- | --- | --- | --- | --- | --- | --- | --- | --- | --- |
| **DSW** | AGE | MEDI_LITE | IPAQ | PSQI | WHO | WAI | BMI | WHTR | METSIR | TYG | WYG | TC/HDL | LDL/HDL | IRCV | SMOKE | ALCOHOL | PHYS_ACT | SLEEP_HR | SLEEP_SAT |
| AGE | 1,000 | 0,930 | 0,030 | 0,007 | 0,063 | 0,000 | 0,004 | 0,015 | 0,008 | 0,000 | 0,000 | 0,000 | 0,005 | 0,000 | 0,202 | 0,033 | 0,009 | 0,006 | 0,051 |
| MEDI_LITE | 0,930 | 1,000 | 0,771 | 0,017 | 0,966 | 0,656 | 0,174 | 0,131 | 0,130 | 0,142 | 0,139 | 0,554 | 0,705 | 0,140 | 0,120 | 0,461 | 0,192 | 0,351 | 0,495 |
| IPAQ | 0,030 | 0,771 | 1,000 | 0,059 | 0,400 | 0,213 | 0,942 | 0,836 | 0,569 | 0,099 | 0,566 | 0,816 | 0,636 | 0,079 | 0,465 | 0,698 | 0,009 | 0,141 | 0,911 |
| PSQI | 0,007 | 0,017 | 0,059 | 1,000 | 0,000 | 0,000 | 0,105 | 0,043 | 0,149 | 0,255 | 0,131 | 0,085 | 0,203 | 0,001 | 0,408 | 0,643 | 0,106 | 0,000 | 0,000 |
| WHO | 0,063 | 0,966 | 0,400 | 0,000 | 1,000 | 0,000 | 0,159 | 0,146 | 0,048 | 0,143 | 0,017 | 0,092 | 0,090 | 0,054 | 0,547 | 0,354 | 0,851 | 0,003 | 0,000 |
| WAI | 0,000 | 0,656 | 0,213 | 0,000 | 0,000 | 1,000 | 0,000 | 0,000 | 0,000 | 0,025 | 0,000 | 0,001 | 0,004 | 0,010 | 0,866 | 0,537 | 0,074 | 0,000 | 0,000 |
| BMI | 0,004 | 0,174 | 0,942 | 0,105 | 0,159 | 0,000 | 1,000 | 0,000 | 0,000 | 0,090 | 0,000 | 0,030 | 0,072 | 0,009 | 0,811 | 0,823 | 0,001 | 0,010 | 0,017 |
| WHTR | 0,015 | 0,131 | 0,836 | 0,043 | 0,146 | 0,000 | 0,000 | 1,000 | 0,000 | 0,102 | 0,000 | 0,009 | 0,072 | 0,005 | 0,991 | 0,866 | 0,001 | 0,004 | 0,008 |
| METSIR | 0,008 | 0,130 | 0,569 | 0,149 | 0,048 | 0,000 | 0,000 | 0,000 | 1,000 | 0,008 | 0,000 | 0,000 | 0,002 | 0,001 | 0,627 | 0,811 | 0,020 | 0,078 | 0,056 |
| TYG | 0,000 | 0,142 | 0,099 | 0,255 | 0,143 | 0,025 | 0,090 | 0,102 | 0,008 | 1,000 | 0,001 | 0,000 | 0,001 | 0,001 | 0,797 | 0,677 | 0,436 | 0,204 | 0,106 |
| WYG | 0,000 | 0,139 | 0,566 | 0,131 | 0,017 | 0,000 | 0,000 | 0,000 | 0,000 | 0,001 | 1,000 | 0,153 | 0,369 | 0,002 | 0,811 | 0,942 | 0,038 | 0,006 | 0,005 |
| TC/HDL | 0,000 | 0,554 | 0,816 | 0,085 | 0,092 | 0,001 | 0,030 | 0,009 | 0,000 | 0,000 | 0,153 | 1,000 | 0,000 | 0,000 | 0,810 | 0,677 | 0,004 | 0,208 | 0,089 |
| LDL/HDL | 0,005 | 0,705 | 0,636 | 0,203 | 0,090 | 0,004 | 0,072 | 0,072 | 0,002 | 0,001 | 0,369 | 0,000 | 1,000 | 0,000 | 0,490 | 0,902 | 0,078 | 0,534 | 0,223 |
| IRCV | 0,000 | 0,140 | 0,079 | 0,001 | 0,054 | 0,010 | 0,009 | 0,005 | 0,001 | 0,001 | 0,002 | 0,000 | 0,000 | 1,000 | 0,021 | 0,783 | 0,067 | 0,066 | 0,100 |
| SMOKE | 0,202 | 0,120 | 0,465 | 0,408 | 0,547 | 0,866 | 0,811 | 0,991 | 0,627 | 0,797 | 0,811 | 0,810 | 0,490 | 0,021 | 1,000 | 0,862 | 0,120 | 0,618 | 0,935 |
| ALCOHOL | 0,033 | 0,461 | 0,698 | 0,643 | 0,354 | 0,537 | 0,823 | 0,866 | 0,811 | 0,677 | 0,942 | 0,677 | 0,902 | 0,783 | 0,862 | 1,000 | 0,215 | 0,667 | 0,513 |
| PHYS_ACT | 0,009 | 0,192 | 0,009 | 0,106 | 0,851 | 0,074 | 0,001 | 0,001 | 0,020 | 0,436 | 0,038 | 0,004 | 0,078 | 0,067 | 0,120 | 0,215 | 1,000 | 0,027 | 0,048 |
| SLEEP_HR | 0,006 | 0,351 | 0,141 | 0,000 | 0,003 | 0,000 | 0,010 | 0,004 | 0,078 | 0,204 | 0,006 | 0,208 | 0,534 | 0,066 | 0,618 | 0,667 | 0,027 | 1,000 | 0,000 |
| SLEEP_SAT | 0,051 | 0,495 | 0,911 | 0,000 | 0,000 | 0,000 | 0,017 | 0,008 | 0,056 | 0,106 | 0,005 | 0,089 | 0,223 | 0,100 | 0,935 | 0,513 | 0,048 | 0,000 | 1,000 |

|  | Mantel's | |
| --- | --- | --- |
| **DSW** | r | p |
| AGE | 0,075 | 0,147 |
| MEDI_LITE | -0,028 | 0,619 |
| IPAQ | 0,586 | 0,001 |
| PSQI | 0,140 | 0,029 |
| WHO | -0,001 | 0,451 |
| WAI | 0,078 | 0,148 |
| BMI | -0,021 | 0,591 |
| WHTR | -0,022 | 0,6 |
| METSIR | -0,054 | 0,743 |
| TYG | -0,148 | 0,995 |
| WYG | -0,077 | 0,845 |
| TC/HDL | -0,133 | 0,986 |
| LDL/HDL | -0,118 | 0,959 |
| IRCV | -0,080 | 0,844 |
| SMOKE | -0,043 | 0,669 |
| ALCOHOL | 0,046 | 0,175 |
| PHYS_ACT | -0,065 | 0,936 |
| SLEEP_HR | 0,106 | 0,08 |
| SLEEP_SAT | -0,071 | 0,851 |

|  | rValue | | | | | | | | | | | | | | | | | | |
| --- | --- | --- | --- | --- | --- | --- | --- | --- | --- | --- | --- | --- | --- | --- | --- | --- | --- | --- | --- |
| **NSW** | AGE | MEDI_LITE | IPAQ | PSQI | WHO | WAI | BMI | WHTR | METSIR | TYG | WYG | TC/HDL | LDL/HDL | IRCV | SMOKE | ALCOHOL | PHYS_ACT | SLEEP_HR | SLEEP_SAT |
| AGE | 1,000 | -0,004 | -0,043 | 0,412 | -0,249 | -0,365 | 0,217 | 0,255 | 0,237 | 0,313 | 0,270 | 0,162 | 0,237 | 0,821 | -0,044 | -0,165 | -0,221 | -0,435 | 0,363 |
| MEDI_LITE | -0,004 | 1,000 | 0,021 | 0,053 | 0,092 | -0,080 | -0,109 | -0,086 | -0,046 | 0,014 | -0,020 | 0,012 | 0,041 | -0,037 | -0,115 | 0,015 | 0,145 | -0,172 | 0,153 |
| IPAQ | -0,043 | 0,021 | 1,000 | -0,005 | 0,072 | -0,079 | -0,141 | -0,110 | -0,140 | -0,226 | -0,184 | -0,161 | -0,129 | -0,011 | 0,177 | 0,206 | 0,248 | -0,028 | -0,118 |
| PSQI | 0,412 | 0,053 | -0,005 | 1,000 | -0,498 | -0,571 | 0,068 | 0,102 | 0,055 | 0,052 | 0,113 | 0,064 | 0,162 | 0,371 | -0,021 | 0,021 | -0,041 | -0,572 | 0,664 |
| WHO | -0,249 | 0,092 | 0,072 | -0,498 | 1,000 | 0,559 | -0,052 | -0,085 | -0,041 | -0,128 | -0,135 | -0,045 | -0,143 | -0,289 | 0,022 | -0,115 | 0,130 | 0,254 | -0,422 |
| WAI | -0,365 | -0,080 | -0,079 | -0,571 | 0,559 | 1,000 | -0,064 | -0,108 | -0,070 | -0,052 | -0,074 | 0,009 | -0,110 | -0,397 | -0,067 | -0,065 | 0,065 | 0,348 | -0,426 |
| BMI | 0,217 | -0,109 | -0,141 | 0,068 | -0,052 | -0,064 | 1,000 | 0,940 | 0,938 | 0,351 | 0,669 | 0,391 | 0,425 | 0,249 | -0,174 | -0,177 | -0,231 | -0,096 | -0,004 |
| WHTR | 0,255 | -0,086 | -0,110 | 0,102 | -0,085 | -0,108 | 0,940 | 1,000 | 0,879 | 0,351 | 0,651 | 0,388 | 0,434 | 0,261 | -0,177 | -0,194 | -0,221 | -0,136 | 0,029 |
| METSIR | 0,237 | -0,046 | -0,140 | 0,055 | -0,041 | -0,070 | 0,938 | 0,879 | 1,000 | 0,499 | 0,721 | 0,594 | 0,586 | 0,345 | -0,116 | -0,158 | -0,210 | -0,127 | 0,024 |
| TYG | 0,313 | 0,014 | -0,226 | 0,052 | -0,128 | -0,052 | 0,351 | 0,351 | 0,499 | 1,000 | 0,515 | 0,450 | 0,378 | 0,368 | -0,140 | -0,145 | -0,173 | -0,140 | 0,168 |
| WYG | 0,270 | -0,020 | -0,184 | 0,113 | -0,135 | -0,074 | 0,669 | 0,651 | 0,721 | 0,515 | 1,000 | 0,391 | 0,396 | 0,338 | -0,162 | -0,124 | -0,192 | -0,217 | 0,129 |
| TC/HDL | 0,162 | 0,012 | -0,161 | 0,064 | -0,045 | 0,009 | 0,391 | 0,388 | 0,594 | 0,450 | 0,391 | 1,000 | 0,973 | 0,362 | 0,007 | -0,128 | -0,155 | -0,005 | 0,107 |
| LDL/HDL | 0,237 | 0,041 | -0,129 | 0,162 | -0,143 | -0,110 | 0,425 | 0,434 | 0,586 | 0,378 | 0,396 | 0,973 | 1,000 | 0,404 | 0,050 | -0,120 | -0,185 | -0,077 | 0,156 |
| IRCV | 0,821 | -0,037 | -0,011 | 0,371 | -0,289 | -0,397 | 0,249 | 0,261 | 0,345 | 0,368 | 0,338 | 0,362 | 0,404 | 1,000 | 0,251 | -0,098 | -0,157 | -0,378 | 0,376 |
| SMOKE | -0,044 | -0,115 | 0,177 | -0,021 | 0,022 | -0,067 | -0,174 | -0,177 | -0,116 | -0,140 | -0,162 | 0,007 | 0,050 | 0,251 | 1,000 | 0,118 | -0,065 | 0,022 | 0,019 |
| ALCOHOL | -0,165 | 0,015 | 0,206 | 0,021 | -0,115 | -0,065 | -0,177 | -0,194 | -0,158 | -0,145 | -0,124 | -0,128 | -0,120 | -0,098 | 0,118 | 1,000 | 0,214 | 0,151 | -0,071 |
| PHYS_ACT | -0,221 | 0,145 | 0,248 | -0,041 | 0,130 | 0,065 | -0,231 | -0,221 | -0,210 | -0,173 | -0,192 | -0,155 | -0,185 | -0,157 | -0,065 | 0,214 | 1,000 | 0,072 | -0,064 |
| SLEEP_HR | -0,435 | -0,172 | -0,028 | -0,572 | 0,254 | 0,348 | -0,096 | -0,136 | -0,127 | -0,140 | -0,217 | -0,005 | -0,077 | -0,378 | 0,022 | 0,151 | 0,072 | 1,000 | -0,546 |
| SLEEP_SAT | 0,363 | 0,153 | -0,118 | 0,664 | -0,422 | -0,426 | -0,004 | 0,029 | 0,024 | 0,168 | 0,129 | 0,107 | 0,156 | 0,376 | 0,019 | -0,071 | -0,064 | -0,546 | 1,000 |

|  | pValue | | | | | | | | | | | | | | | | | | |
| --- | --- | --- | --- | --- | --- | --- | --- | --- | --- | --- | --- | --- | --- | --- | --- | --- | --- | --- | --- |
| **NSW** | AGE | MEDI_LITE | IPAQ | PSQI | WHO | WAI | BMI | WHTR | METSIR | TYG | WYG | TC/HDL | LDL/HDL | IRCV | SMOKE | ALCOHOL | PHYS_ACT | SLEEP_HR | SLEEP_SAT |
| AGE | 1,000 | 0,964 | 0,606 | 0,000 | 0,002 | 0,000 | 0,008 | 0,002 | 0,008 | 0,000 | 0,001 | 0,070 | 0,011 | 0,000 | 0,599 | 0,046 | 0,007 | 0,000 | 0,000 |
| MEDI_LITE | 0,964 | 1,000 | 0,803 | 0,520 | 0,269 | 0,337 | 0,190 | 0,302 | 0,613 | 0,875 | 0,810 | 0,893 | 0,662 | 0,680 | 0,166 | 0,853 | 0,080 | 0,037 | 0,064 |
| IPAQ | 0,606 | 0,803 | 1,000 | 0,951 | 0,389 | 0,344 | 0,089 | 0,183 | 0,121 | 0,011 | 0,026 | 0,072 | 0,173 | 0,906 | 0,032 | 0,012 | 0,002 | 0,733 | 0,153 |
| PSQI | 0,000 | 0,520 | 0,951 | 1,000 | 0,000 | 0,000 | 0,411 | 0,220 | 0,545 | 0,566 | 0,174 | 0,481 | 0,085 | 0,000 | 0,803 | 0,802 | 0,624 | 0,000 | 0,000 |
| WHO | 0,002 | 0,269 | 0,389 | 0,000 | 1,000 | 0,000 | 0,531 | 0,309 | 0,653 | 0,157 | 0,105 | 0,620 | 0,130 | 0,001 | 0,789 | 0,165 | 0,118 | 0,002 | 0,000 |
| WAI | 0,000 | 0,337 | 0,344 | 0,000 | 0,000 | 1,000 | 0,440 | 0,193 | 0,440 | 0,568 | 0,374 | 0,921 | 0,243 | 0,000 | 0,417 | 0,435 | 0,431 | 0,000 | 0,000 |
| BMI | 0,008 | 0,190 | 0,089 | 0,411 | 0,531 | 0,440 | 1,000 | 0,000 | 0,000 | 0,000 | 0,000 | 0,000 | 0,000 | 0,005 | 0,035 | 0,032 | 0,005 | 0,246 | 0,958 |
| WHTR | 0,002 | 0,302 | 0,183 | 0,220 | 0,309 | 0,193 | 0,000 | 1,000 | 0,000 | 0,000 | 0,000 | 0,000 | 0,000 | 0,003 | 0,032 | 0,018 | 0,007 | 0,100 | 0,726 |
| METSIR | 0,008 | 0,613 | 0,121 | 0,545 | 0,653 | 0,440 | 0,000 | 0,000 | 1,000 | 0,000 | 0,000 | 0,000 | 0,000 | 0,000 | 0,199 | 0,080 | 0,019 | 0,159 | 0,791 |
| TYG | 0,000 | 0,875 | 0,011 | 0,566 | 0,157 | 0,568 | 0,000 | 0,000 | 0,000 | 1,000 | 0,000 | 0,000 | 0,000 | 0,000 | 0,122 | 0,107 | 0,055 | 0,122 | 0,062 |
| WYG | 0,001 | 0,810 | 0,026 | 0,174 | 0,105 | 0,374 | 0,000 | 0,000 | 0,000 | 0,000 | 1,000 | 0,000 | 0,000 | 0,000 | 0,051 | 0,136 | 0,020 | 0,009 | 0,122 |
| TC/HDL | 0,070 | 0,893 | 0,072 | 0,481 | 0,620 | 0,921 | 0,000 | 0,000 | 0,000 | 0,000 | 0,000 | 1,000 | 0,000 | 0,000 | 0,937 | 0,155 | 0,084 | 0,952 | 0,234 |
| LDL/HDL | 0,011 | 0,662 | 0,173 | 0,085 | 0,130 | 0,243 | 0,000 | 0,000 | 0,000 | 0,000 | 0,000 | 0,000 | 1,000 | 0,000 | 0,600 | 0,202 | 0,049 | 0,416 | 0,097 |
| IRCV | 0,000 | 0,680 | 0,906 | 0,000 | 0,001 | 0,000 | 0,005 | 0,003 | 0,000 | 0,000 | 0,000 | 0,000 | 0,000 | 1,000 | 0,005 | 0,281 | 0,081 | 0,000 | 0,000 |
| SMOKE | 0,599 | 0,166 | 0,032 | 0,803 | 0,789 | 0,417 | 0,035 | 0,032 | 0,199 | 0,122 | 0,051 | 0,937 | 0,600 | 0,005 | 1,000 | 0,156 | 0,436 | 0,787 | 0,824 |
| ALCOHOL | 0,046 | 0,853 | 0,012 | 0,802 | 0,165 | 0,435 | 0,032 | 0,018 | 0,080 | 0,107 | 0,136 | 0,155 | 0,202 | 0,281 | 0,156 | 1,000 | 0,009 | 0,068 | 0,391 |
| PHYS_ACT | 0,007 | 0,080 | 0,002 | 0,624 | 0,118 | 0,431 | 0,005 | 0,007 | 0,019 | 0,055 | 0,020 | 0,084 | 0,049 | 0,081 | 0,436 | 0,009 | 1,000 | 0,389 | 0,443 |
| SLEEP_HR | 0,000 | 0,037 | 0,733 | 0,000 | 0,002 | 0,000 | 0,246 | 0,100 | 0,159 | 0,122 | 0,009 | 0,952 | 0,416 | 0,000 | 0,787 | 0,068 | 0,389 | 1,000 | 0,000 |
| SLEEP_SAT | 0,000 | 0,064 | 0,153 | 0,000 | 0,000 | 0,000 | 0,958 | 0,726 | 0,791 | 0,062 | 0,122 | 0,234 | 0,097 | 0,000 | 0,824 | 0,391 | 0,443 | 0,000 | 1,000 |

|  | Mantel's | |
| --- | --- | --- |
| **NSW** | r | p |
| AGE | -0,020 | 0,712 |
| MEDI_LITE | -0,008 | 0,557 |
| IPAQ | 0,565 | 0,001 |
| PSQI | 0,064 | 0,051 |
| WHO | 0,080 | 0,027 |
| WAI | 0,143 | 0,004 |
| BMI | 0,008 | 0,427 |
| WHTR | -0,018 | 0,692 |
| METSIR | 0,005 | 0,434 |
| TYG | -0,018 | 0,673 |
| WYG | 0,010 | 0,393 |
| TC/HDL | -0,015 | 0,610 |
| LDL/HDL | -0,026 | 0,715 |
| IRCV | -0,047 | 0,819 |
| SMOKE | 0,037 | 0,089 |
| ALCOHOL | 0,035 | 0,099 |
| PHYS_ACT | 0,064 | 0,004 |
| SLEEP_HR | 0,030 | 0,237 |
| SLEEP_SAT | 0,042 | 0,097 |

|  | rValue | | | | | | | | | | | | | | | | | | | | |
| --- | --- | --- | --- | --- | --- | --- | --- | --- | --- | --- | --- | --- | --- | --- | --- | --- | --- | --- | --- | --- | --- |
| **LOW** | NSW_CUM | NSW_YRS | AGE | MEDI_LITE | IPAQ | PSQI | WHO | WAI | BMI | WHTR | METSIR | TYG | WYG | TC/HDL | LDL/HDL | IRCV | SMOKE | ALCOHOL | PHYS_ACT | SLEEP_HR | SLEEP_SAT |
| NSW_CUM | 1,000 | 0,698 | -0,063 | -0,027 | 0,076 | 0,124 | -0,152 | -0,134 | 0,086 | 0,031 | 0,115 | -0,126 | -0,011 | 0,071 | -0,022 | -0,168 | 0,124 | 0,072 | -0,186 | 0,021 | 0,031 |
| NSW_YRS | 0,698 | 1,000 | -0,192 | 0,002 | 0,023 | -0,161 | -0,192 | 0,012 | 0,034 | 0,012 | 0,120 | 0,057 | 0,052 | 0,091 | -0,011 | -0,184 | 0,094 | 0,309 | 0,003 | 0,152 | -0,091 |
| AGE | -0,063 | -0,192 | 1,000 | -0,059 | -0,120 | 0,237 | -0,146 | -0,258 | 0,311 | 0,295 | 0,291 | 0,331 | 0,255 | 0,196 | 0,146 | 0,820 | -0,045 | -0,348 | -0,362 | -0,284 | 0,314 |
| MEDI_LITE | -0,027 | 0,002 | -0,059 | 1,000 | 0,045 | -0,029 | -0,059 | -0,228 | -0,235 | -0,205 | -0,211 | -0,074 | -0,029 | -0,100 | -0,119 | -0,300 | -0,095 | -0,039 | 0,099 | -0,363 | -0,028 |
| IPAQ | 0,076 | 0,023 | -0,120 | 0,045 | 1,000 | -0,018 | 0,039 | -0,235 | -0,025 | 0,035 | -0,013 | -0,100 | 0,013 | -0,042 | -0,033 | 0,083 | 0,189 | 0,040 | -0,100 | -0,076 | -0,046 |
| PSQI | 0,124 | -0,161 | 0,237 | -0,029 | -0,018 | 1,000 | -0,297 | -0,375 | 0,102 | 0,092 | -0,007 | -0,216 | 0,001 | 0,043 | 0,157 | 0,326 | 0,055 | -0,181 | -0,057 | -0,449 | 0,782 |
| WHO | -0,152 | -0,192 | -0,146 | -0,059 | 0,039 | -0,297 | 1,000 | 0,383 | -0,191 | -0,245 | -0,097 | -0,124 | -0,200 | -0,003 | -0,071 | -0,153 | -0,001 | -0,125 | -0,004 | 0,102 | -0,279 |
| WAI | -0,134 | 0,012 | -0,258 | -0,228 | -0,235 | -0,375 | 0,383 | 1,000 | -0,169 | -0,215 | -0,218 | 0,139 | -0,157 | -0,052 | -0,102 | -0,372 | -0,062 | 0,086 | -0,005 | 0,307 | -0,410 |
| BMI | 0,086 | 0,034 | 0,311 | -0,235 | -0,025 | 0,102 | -0,191 | -0,169 | 1,000 | 0,940 | 0,919 | 0,357 | 0,580 | 0,452 | 0,438 | 0,403 | -0,216 | -0,070 | -0,283 | 0,052 | 0,237 |
| WHTR | 0,031 | 0,012 | 0,295 | -0,205 | 0,035 | 0,092 | -0,245 | -0,215 | 0,940 | 1,000 | 0,872 | 0,345 | 0,535 | 0,396 | 0,364 | 0,376 | -0,257 | -0,084 | -0,240 | 0,019 | 0,228 |
| METSIR | 0,115 | 0,120 | 0,291 | -0,211 | -0,013 | -0,007 | -0,097 | -0,218 | 0,919 | 0,872 | 1,000 | 0,490 | 0,591 | 0,651 | 0,606 | 0,444 | -0,130 | -0,063 | -0,282 | -0,013 | 0,234 |
| TYG | -0,126 | 0,057 | 0,331 | -0,074 | -0,100 | -0,216 | -0,124 | 0,139 | 0,357 | 0,345 | 0,490 | 1,000 | 0,233 | 0,479 | 0,373 | 0,363 | -0,069 | 0,115 | -0,276 | 0,053 | -0,055 |
| WYG | -0,011 | 0,052 | 0,255 | -0,029 | 0,013 | 0,001 | -0,200 | -0,157 | 0,580 | 0,535 | 0,591 | 0,233 | 1,000 | 0,348 | 0,387 | 0,404 | -0,032 | -0,026 | -0,032 | -0,007 | 0,176 |
| TC/HDL | 0,071 | 0,091 | 0,196 | -0,100 | -0,042 | 0,043 | -0,003 | -0,052 | 0,452 | 0,396 | 0,651 | 0,479 | 0,348 | 1,000 | 0,939 | 0,422 | -0,049 | -0,196 | -0,246 | -0,038 | 0,272 |
| LDL/HDL | -0,022 | -0,011 | 0,146 | -0,119 | -0,033 | 0,157 | -0,071 | -0,102 | 0,438 | 0,364 | 0,606 | 0,373 | 0,387 | 0,939 | 1,000 | 0,399 | -0,029 | -0,257 | -0,179 | -0,113 | 0,374 |
| IRCV | -0,168 | -0,184 | 0,820 | -0,300 | 0,083 | 0,326 | -0,153 | -0,372 | 0,403 | 0,376 | 0,444 | 0,363 | 0,404 | 0,422 | 0,399 | 1,000 | 0,423 | -0,312 | -0,361 | -0,341 | 0,508 |
| SMOKE | 0,124 | 0,094 | -0,045 | -0,095 | 0,189 | 0,055 | -0,001 | -0,062 | -0,216 | -0,257 | -0,130 | -0,069 | -0,032 | -0,049 | -0,029 | 0,423 | 1,000 | 0,189 | 0,045 | 0,019 | 0,021 |
| ALCOHOL | 0,072 | 0,309 | -0,348 | -0,039 | 0,040 | -0,181 | -0,125 | 0,086 | -0,070 | -0,084 | -0,063 | 0,115 | -0,026 | -0,196 | -0,257 | -0,312 | 0,189 | 1,000 | 0,388 | 0,416 | -0,177 |
| PHYS_ACT | -0,186 | 0,003 | -0,362 | 0,099 | -0,100 | -0,057 | -0,004 | -0,005 | -0,283 | -0,240 | -0,282 | -0,276 | -0,032 | -0,246 | -0,179 | -0,361 | 0,045 | 0,388 | 1,000 | 0,035 | -0,053 |
| SLEEP_HR | 0,021 | 0,152 | -0,284 | -0,363 | -0,076 | -0,449 | 0,102 | 0,307 | 0,052 | 0,019 | -0,013 | 0,053 | -0,007 | -0,038 | -0,113 | -0,341 | 0,019 | 0,416 | 0,035 | 1,000 | -0,448 |
| SLEEP_SAT | 0,031 | -0,091 | 0,314 | -0,028 | -0,046 | 0,782 | -0,279 | -0,410 | 0,237 | 0,228 | 0,234 | -0,055 | 0,176 | 0,272 | 0,374 | 0,508 | 0,021 | -0,177 | -0,053 | -0,448 | 1,000 |

|  | pValue | | | | | | | | | | | | | | | | | | | | |
| --- | --- | --- | --- | --- | --- | --- | --- | --- | --- | --- | --- | --- | --- | --- | --- | --- | --- | --- | --- | --- | --- |
| **LOW** | NSW_CUM | NSW_YRS | AGE | MEDI_LITE | IPAQ | PSQI | WHO | WAI | BMI | WHTR | METSIR | TYG | WYG | TC/HDL | LDL/HDL | IRCV | SMOKE | ALCOHOL | PHYS_ACT | SLEEP_HR | SLEEP_SAT |
| NSW_CUM | 1,000 | 0,000 | 0,659 | 0,851 | 0,595 | 0,386 | 0,286 | 0,350 | 0,548 | 0,829 | 0,518 | 0,476 | 0,938 | 0,691 | 0,903 | 0,341 | 0,386 | 0,616 | 0,191 | 0,886 | 0,827 |
| NSW_YRS | 0,000 | 1,000 | 0,176 | 0,992 | 0,872 | 0,258 | 0,177 | 0,934 | 0,811 | 0,932 | 0,499 | 0,750 | 0,716 | 0,607 | 0,951 | 0,297 | 0,511 | 0,028 | 0,985 | 0,288 | 0,524 |
| AGE | 0,659 | 0,176 | 1,000 | 0,680 | 0,402 | 0,095 | 0,307 | 0,068 | 0,026 | 0,036 | 0,095 | 0,056 | 0,071 | 0,267 | 0,417 | 0,000 | 0,754 | 0,012 | 0,009 | 0,043 | 0,025 |
| MEDI_LITE | 0,851 | 0,992 | 0,680 | 1,000 | 0,753 | 0,838 | 0,682 | 0,107 | 0,097 | 0,150 | 0,231 | 0,676 | 0,840 | 0,574 | 0,511 | 0,084 | 0,506 | 0,786 | 0,491 | 0,009 | 0,846 |
| IPAQ | 0,595 | 0,872 | 0,402 | 0,753 | 1,000 | 0,901 | 0,786 | 0,098 | 0,862 | 0,806 | 0,942 | 0,574 | 0,926 | 0,814 | 0,856 | 0,642 | 0,183 | 0,779 | 0,485 | 0,597 | 0,748 |
| PSQI | 0,386 | 0,258 | 0,095 | 0,838 | 0,901 | 1,000 | 0,035 | 0,007 | 0,475 | 0,519 | 0,968 | 0,220 | 0,993 | 0,808 | 0,382 | 0,060 | 0,703 | 0,204 | 0,692 | 0,001 | 0,000 |
| WHO | 0,286 | 0,177 | 0,307 | 0,682 | 0,786 | 0,035 | 1,000 | 0,005 | 0,178 | 0,083 | 0,585 | 0,484 | 0,159 | 0,988 | 0,694 | 0,388 | 0,994 | 0,383 | 0,978 | 0,474 | 0,047 |
| WAI | 0,350 | 0,934 | 0,068 | 0,107 | 0,098 | 0,007 | 0,005 | 1,000 | 0,237 | 0,130 | 0,215 | 0,431 | 0,271 | 0,771 | 0,573 | 0,030 | 0,666 | 0,549 | 0,970 | 0,028 | 0,003 |
| BMI | 0,548 | 0,811 | 0,026 | 0,097 | 0,862 | 0,475 | 0,178 | 0,237 | 1,000 | 0,000 | 0,000 | 0,038 | 0,000 | 0,007 | 0,011 | 0,018 | 0,128 | 0,626 | 0,044 | 0,716 | 0,093 |
| WHTR | 0,829 | 0,932 | 0,036 | 0,150 | 0,806 | 0,519 | 0,083 | 0,130 | 0,000 | 1,000 | 0,000 | 0,045 | 0,000 | 0,021 | 0,037 | 0,028 | 0,068 | 0,558 | 0,090 | 0,896 | 0,108 |
| METSIR | 0,518 | 0,499 | 0,095 | 0,231 | 0,942 | 0,968 | 0,585 | 0,215 | 0,000 | 0,000 | 1,000 | 0,003 | 0,000 | 0,000 | 0,000 | 0,009 | 0,465 | 0,723 | 0,106 | 0,943 | 0,183 |
| TYG | 0,476 | 0,750 | 0,056 | 0,676 | 0,574 | 0,220 | 0,484 | 0,431 | 0,038 | 0,045 | 0,003 | 1,000 | 0,185 | 0,004 | 0,032 | 0,035 | 0,696 | 0,517 | 0,114 | 0,765 | 0,758 |
| WYG | 0,938 | 0,716 | 0,071 | 0,840 | 0,926 | 0,993 | 0,159 | 0,271 | 0,000 | 0,000 | 0,000 | 0,185 | 1,000 | 0,044 | 0,026 | 0,018 | 0,822 | 0,855 | 0,823 | 0,960 | 0,215 |
| TC/HDL | 0,691 | 0,607 | 0,267 | 0,574 | 0,814 | 0,808 | 0,988 | 0,771 | 0,007 | 0,021 | 0,000 | 0,004 | 0,044 | 1,000 | 0,000 | 0,013 | 0,781 | 0,265 | 0,160 | 0,832 | 0,120 |
| LDL/HDL | 0,903 | 0,951 | 0,417 | 0,511 | 0,856 | 0,382 | 0,694 | 0,573 | 0,011 | 0,037 | 0,000 | 0,032 | 0,026 | 0,000 | 1,000 | 0,022 | 0,871 | 0,149 | 0,319 | 0,532 | 0,032 |
| IRCV | 0,341 | 0,297 | 0,000 | 0,084 | 0,642 | 0,060 | 0,388 | 0,030 | 0,018 | 0,028 | 0,009 | 0,035 | 0,018 | 0,013 | 0,022 | 1,000 | 0,013 | 0,073 | 0,036 | 0,048 | 0,002 |
| SMOKE | 0,386 | 0,511 | 0,754 | 0,506 | 0,183 | 0,703 | 0,994 | 0,666 | 0,128 | 0,068 | 0,465 | 0,696 | 0,822 | 0,781 | 0,871 | 0,013 | 1,000 | 0,184 | 0,754 | 0,897 | 0,886 |
| ALCOHOL | 0,616 | 0,028 | 0,012 | 0,786 | 0,779 | 0,204 | 0,383 | 0,549 | 0,626 | 0,558 | 0,723 | 0,517 | 0,855 | 0,265 | 0,149 | 0,073 | 0,184 | 1,000 | 0,005 | 0,002 | 0,213 |
| PHYS_ACT | 0,191 | 0,985 | 0,009 | 0,491 | 0,485 | 0,692 | 0,978 | 0,970 | 0,044 | 0,090 | 0,106 | 0,114 | 0,823 | 0,160 | 0,319 | 0,036 | 0,754 | 0,005 | 1,000 | 0,808 | 0,710 |
| SLEEP_HR | 0,886 | 0,288 | 0,043 | 0,009 | 0,597 | 0,001 | 0,474 | 0,028 | 0,716 | 0,896 | 0,943 | 0,765 | 0,960 | 0,832 | 0,532 | 0,048 | 0,897 | 0,002 | 0,808 | 1,000 | 0,001 |
| SLEEP_SAT | 0,827 | 0,524 | 0,025 | 0,846 | 0,748 | 0,000 | 0,047 | 0,003 | 0,093 | 0,108 | 0,183 | 0,758 | 0,215 | 0,120 | 0,032 | 0,002 | 0,886 | 0,213 | 0,710 | 0,001 | 1,000 |

|  | Mantel's | |
| --- | --- | --- |
| **LOW** | r | p |
| NSW_CUM | 0,073 | 0,110 |
| NSW_YRS | 0,068 | 0,178 |
| AGE | 0,109 | 0,056 |
| MEDI_LITE | -0,057 | 0,762 |
| IPAQ | 0,710 | 0,001 |
| PSQI | -0,057 | 0,751 |
| WHO | 0,035 | 0,297 |
| WAI | 0,145 | 0,033 |
| BMI | 0,105 | 0,115 |
| WHTR | 0,077 | 0,159 |
| METSIR | 0,054 | 0,230 |
| TYG | 0,073 | 0,147 |
| WYG | 0,020 | 0,411 |
| TC/HDL | 0,061 | 0,234 |
| LDL/HDL | 0,052 | 0,278 |
| IRCV | 0,228 | 0,022 |
| SMOKE | 0,132 | 0,071 |
| ALCOHOL | 0,046 | 0,243 |
| PHYS_ACT | 0,064 | 0,065 |
| SLEEP_HR | 0,019 | 0,388 |
| SLEEP_SAT | -0,003 | 0,497 |

|  | rValue | | | | | | | | | | | | | | | | | | | | |
| --- | --- | --- | --- | --- | --- | --- | --- | --- | --- | --- | --- | --- | --- | --- | --- | --- | --- | --- | --- | --- | --- |
| **HIGH** | NSW_CUM | NSW_YRS | AGE | MEDI_LITE | IPAQ | PSQI | WHO | WAI | BMI | WHTR | METSIR | TYG | WYG | TC/HDL | LDL/HDL | IRCV | SMOKE | ALCOHOL | PHYS_ACT | SLEEP_HR | SLEEP_SAT |
| NSW_CUM | 1,000 | 0,914 | 0,590 | -0,046 | 0,028 | 0,382 | -0,285 | -0,316 | 0,138 | 0,147 | 0,167 | 0,213 | 0,207 | 0,163 | 0,205 | 0,486 | -0,070 | -0,003 | 0,002 | -0,355 | 0,248 |
| NSW_YRS | 0,914 | 1,000 | 0,566 | -0,102 | 0,093 | 0,345 | -0,257 | -0,281 | 0,114 | 0,106 | 0,111 | 0,123 | 0,168 | 0,063 | 0,102 | 0,450 | -0,016 | -0,010 | -0,009 | -0,316 | 0,199 |
| AGE | 0,590 | 0,566 | 1,000 | 0,027 | 0,058 | 0,474 | -0,284 | -0,393 | 0,052 | 0,086 | 0,105 | 0,277 | 0,180 | 0,150 | 0,239 | 0,748 | -0,206 | 0,053 | -0,037 | -0,449 | 0,268 |
| MEDI_LITE | -0,046 | -0,102 | 0,027 | 1,000 | 0,015 | 0,094 | 0,161 | -0,007 | -0,056 | -0,043 | -0,028 | 0,021 | -0,030 | 0,025 | 0,060 | -0,006 | -0,132 | 0,047 | 0,172 | -0,093 | 0,263 |
| IPAQ | 0,028 | 0,093 | 0,058 | 0,015 | 1,000 | 0,031 | 0,060 | -0,039 | -0,164 | -0,143 | -0,167 | -0,261 | -0,225 | -0,185 | -0,132 | -0,009 | 0,199 | 0,252 | 0,376 | -0,031 | -0,125 |
| PSQI | 0,382 | 0,345 | 0,474 | 0,094 | 0,031 | 1,000 | -0,567 | -0,637 | 0,006 | 0,056 | 0,018 | 0,092 | 0,114 | 0,056 | 0,164 | 0,355 | -0,091 | 0,158 | 0,008 | -0,593 | 0,578 |
| WHO | -0,285 | -0,257 | -0,284 | 0,161 | 0,060 | -0,567 | 1,000 | 0,619 | 0,014 | -0,018 | 0,000 | -0,126 | -0,089 | -0,054 | -0,159 | -0,333 | 0,044 | -0,135 | 0,176 | 0,315 | -0,473 |
| WAI | -0,316 | -0,281 | -0,393 | -0,007 | -0,039 | -0,637 | 0,619 | 1,000 | 0,005 | -0,040 | 0,012 | -0,091 | -0,009 | 0,041 | -0,093 | -0,371 | -0,043 | -0,173 | 0,061 | 0,329 | -0,398 |
| BMI | 0,138 | 0,114 | 0,052 | -0,056 | -0,164 | 0,006 | 0,014 | 0,005 | 1,000 | 0,934 | 0,936 | 0,330 | 0,695 | 0,366 | 0,399 | 0,136 | -0,188 | -0,191 | -0,178 | -0,136 | -0,183 |
| WHTR | 0,147 | 0,106 | 0,086 | -0,043 | -0,143 | 0,056 | -0,018 | -0,040 | 0,934 | 1,000 | 0,868 | 0,339 | 0,687 | 0,380 | 0,430 | 0,161 | -0,175 | -0,212 | -0,175 | -0,181 | -0,121 |
| METSIR | 0,167 | 0,111 | 0,105 | -0,028 | -0,167 | 0,018 | 0,000 | 0,012 | 0,936 | 0,868 | 1,000 | 0,481 | 0,750 | 0,580 | 0,571 | 0,266 | -0,136 | -0,171 | -0,172 | -0,144 | -0,116 |
| TYG | 0,213 | 0,123 | 0,277 | 0,021 | -0,261 | 0,092 | -0,126 | -0,091 | 0,330 | 0,339 | 0,481 | 1,000 | 0,554 | 0,444 | 0,355 | 0,359 | -0,214 | -0,190 | -0,117 | -0,196 | 0,200 |
| WYG | 0,207 | 0,168 | 0,180 | -0,030 | -0,225 | 0,114 | -0,089 | -0,009 | 0,695 | 0,687 | 0,750 | 0,554 | 1,000 | 0,389 | 0,349 | 0,204 | -0,252 | -0,118 | -0,239 | -0,303 | 0,070 |
| TC/HDL | 0,163 | 0,063 | 0,150 | 0,025 | -0,185 | 0,056 | -0,054 | 0,041 | 0,366 | 0,380 | 0,580 | 0,444 | 0,389 | 1,000 | 0,983 | 0,394 | 0,028 | -0,101 | -0,129 | 0,012 | 0,048 |
| LDL/HDL | 0,205 | 0,102 | 0,239 | 0,060 | -0,132 | 0,164 | -0,159 | -0,093 | 0,399 | 0,430 | 0,571 | 0,355 | 0,349 | 0,983 | 1,000 | 0,424 | 0,070 | -0,055 | -0,171 | -0,070 | 0,064 |
| IRCV | 0,486 | 0,450 | 0,748 | -0,006 | -0,009 | 0,355 | -0,333 | -0,371 | 0,136 | 0,161 | 0,266 | 0,359 | 0,204 | 0,394 | 0,424 | 1,000 | 0,167 | 0,034 | -0,041 | -0,362 | 0,292 |
| SMOKE | -0,070 | -0,016 | -0,206 | -0,132 | 0,199 | -0,091 | 0,044 | -0,043 | -0,188 | -0,175 | -0,136 | -0,214 | -0,252 | 0,028 | 0,070 | 0,167 | 1,000 | 0,127 | -0,092 | 0,079 | -0,034 |
| ALCOHOL | -0,003 | -0,010 | 0,053 | 0,047 | 0,252 | 0,158 | -0,135 | -0,173 | -0,191 | -0,212 | -0,171 | -0,190 | -0,118 | -0,101 | -0,055 | 0,034 | 0,127 | 1,000 | 0,102 | -0,018 | 0,040 |
| PHYS_ACT | 0,002 | -0,009 | -0,037 | 0,172 | 0,376 | 0,008 | 0,176 | 0,061 | -0,178 | -0,175 | -0,172 | -0,117 | -0,239 | -0,129 | -0,171 | -0,041 | -0,092 | 0,102 | 1,000 | 0,037 | -0,008 |
| SLEEP_HR | -0,355 | -0,316 | -0,449 | -0,093 | -0,031 | -0,593 | 0,315 | 0,329 | -0,136 | -0,181 | -0,144 | -0,196 | -0,303 | 0,012 | -0,070 | -0,362 | 0,079 | -0,018 | 0,037 | 1,000 | -0,527 |
| SLEEP_SAT | 0,248 | 0,199 | 0,268 | 0,263 | -0,125 | 0,578 | -0,473 | -0,398 | -0,183 | -0,121 | -0,116 | 0,200 | 0,070 | 0,048 | 0,064 | 0,292 | -0,034 | 0,040 | -0,008 | -0,527 | 1,000 |

|  | pValue | | | | | | | | | | | | | | | | | | | | |
| --- | --- | --- | --- | --- | --- | --- | --- | --- | --- | --- | --- | --- | --- | --- | --- | --- | --- | --- | --- | --- | --- |
| **HIGH** | NSW_CUM | NSW_YRS | AGE | MEDI_LITE | IPAQ | PSQI | WHO | WAI | BMI | WHTR | METSIR | TYG | WYG | TC/HDL | LDL/HDL | IRCV | SMOKE | ALCOHOL | PHYS_ACT | SLEEP_HR | SLEEP_SAT |
| NSW_CUM | 1,000 | 0,000 | 0,000 | 0,659 | 0,789 | 0,000 | 0,005 | 0,002 | 0,182 | 0,154 | 0,115 | 0,044 | 0,045 | 0,124 | 0,066 | 0,000 | 0,499 | 0,976 | 0,988 | 0,000 | 0,016 |
| NSW_YRS | 0,000 | 1,000 | 0,000 | 0,324 | 0,372 | 0,001 | 0,012 | 0,006 | 0,270 | 0,306 | 0,299 | 0,250 | 0,106 | 0,551 | 0,364 | 0,000 | 0,876 | 0,927 | 0,933 | 0,002 | 0,053 |
| AGE | 0,000 | 0,000 | 1,000 | 0,795 | 0,578 | 0,000 | 0,005 | 0,000 | 0,615 | 0,407 | 0,324 | 0,008 | 0,083 | 0,157 | 0,032 | 0,000 | 0,046 | 0,607 | 0,723 | 0,000 | 0,009 |
| MEDI_LITE | 0,659 | 0,324 | 0,795 | 1,000 | 0,882 | 0,366 | 0,118 | 0,944 | 0,591 | 0,679 | 0,792 | 0,845 | 0,777 | 0,813 | 0,596 | 0,955 | 0,201 | 0,652 | 0,095 | 0,372 | 0,010 |
| IPAQ | 0,789 | 0,372 | 0,578 | 0,882 | 1,000 | 0,768 | 0,565 | 0,707 | 0,113 | 0,166 | 0,116 | 0,013 | 0,029 | 0,079 | 0,242 | 0,931 | 0,053 | 0,014 | 0,000 | 0,764 | 0,228 |
| PSQI | 0,000 | 0,001 | 0,000 | 0,366 | 0,768 | 1,000 | 0,000 | 0,000 | 0,957 | 0,592 | 0,867 | 0,389 | 0,272 | 0,597 | 0,143 | 0,001 | 0,381 | 0,126 | 0,936 | 0,000 | 0,000 |
| WHO | 0,005 | 0,012 | 0,005 | 0,118 | 0,565 | 0,000 | 1,000 | 0,000 | 0,894 | 0,865 | 0,998 | 0,237 | 0,393 | 0,613 | 0,155 | 0,001 | 0,674 | 0,192 | 0,087 | 0,002 | 0,000 |
| WAI | 0,002 | 0,006 | 0,000 | 0,944 | 0,707 | 0,000 | 0,000 | 1,000 | 0,959 | 0,699 | 0,908 | 0,394 | 0,929 | 0,703 | 0,409 | 0,000 | 0,681 | 0,093 | 0,554 | 0,001 | 0,000 |
| BMI | 0,182 | 0,270 | 0,615 | 0,591 | 0,113 | 0,957 | 0,894 | 0,959 | 1,000 | 0,000 | 0,000 | 0,001 | 0,000 | 0,000 | 0,000 | 0,200 | 0,067 | 0,064 | 0,084 | 0,189 | 0,076 |
| WHTR | 0,154 | 0,306 | 0,407 | 0,679 | 0,166 | 0,592 | 0,865 | 0,699 | 0,000 | 1,000 | 0,000 | 0,001 | 0,000 | 0,000 | 0,000 | 0,129 | 0,090 | 0,039 | 0,090 | 0,080 | 0,244 |
| METSIR | 0,115 | 0,299 | 0,324 | 0,792 | 0,116 | 0,867 | 0,998 | 0,908 | 0,000 | 0,000 | 1,000 | 0,000 | 0,000 | 0,000 | 0,000 | 0,012 | 0,201 | 0,108 | 0,105 | 0,174 | 0,275 |
| TYG | 0,044 | 0,250 | 0,008 | 0,845 | 0,013 | 0,389 | 0,237 | 0,394 | 0,001 | 0,001 | 0,000 | 1,000 | 0,000 | 0,000 | 0,001 | 0,001 | 0,043 | 0,073 | 0,270 | 0,064 | 0,059 |
| WYG | 0,045 | 0,106 | 0,083 | 0,777 | 0,029 | 0,272 | 0,393 | 0,929 | 0,000 | 0,000 | 0,000 | 0,000 | 1,000 | 0,000 | 0,001 | 0,054 | 0,014 | 0,256 | 0,020 | 0,003 | 0,505 |
| TC/HDL | 0,124 | 0,551 | 0,157 | 0,813 | 0,079 | 0,597 | 0,613 | 0,703 | 0,000 | 0,000 | 0,000 | 0,000 | 0,000 | 1,000 | 0,000 | 0,000 | 0,791 | 0,342 | 0,224 | 0,909 | 0,650 |
| LDL/HDL | 0,066 | 0,364 | 0,032 | 0,596 | 0,242 | 0,143 | 0,155 | 0,409 | 0,000 | 0,000 | 0,000 | 0,001 | 0,001 | 0,000 | 1,000 | 0,000 | 0,537 | 0,628 | 0,127 | 0,536 | 0,572 |
| IRCV | 0,000 | 0,000 | 0,000 | 0,955 | 0,931 | 0,001 | 0,001 | 0,000 | 0,200 | 0,129 | 0,012 | 0,001 | 0,054 | 0,000 | 0,000 | 1,000 | 0,116 | 0,754 | 0,701 | 0,000 | 0,005 |
| SMOKE | 0,499 | 0,876 | 0,046 | 0,201 | 0,053 | 0,381 | 0,674 | 0,681 | 0,067 | 0,090 | 0,201 | 0,043 | 0,014 | 0,791 | 0,537 | 0,116 | 1,000 | 0,220 | 0,373 | 0,446 | 0,745 |
| ALCOHOL | 0,976 | 0,927 | 0,607 | 0,652 | 0,014 | 0,126 | 0,192 | 0,093 | 0,064 | 0,039 | 0,108 | 0,073 | 0,256 | 0,342 | 0,628 | 0,754 | 0,220 | 1,000 | 0,326 | 0,859 | 0,699 |
| PHYS_ACT | 0,988 | 0,933 | 0,723 | 0,095 | 0,000 | 0,936 | 0,087 | 0,554 | 0,084 | 0,090 | 0,105 | 0,270 | 0,020 | 0,224 | 0,127 | 0,701 | 0,373 | 0,326 | 1,000 | 0,724 | 0,939 |
| SLEEP_HR | 0,000 | 0,002 | 0,000 | 0,372 | 0,764 | 0,000 | 0,002 | 0,001 | 0,189 | 0,080 | 0,174 | 0,064 | 0,003 | 0,909 | 0,536 | 0,000 | 0,446 | 0,859 | 0,724 | 1,000 | 0,000 |
| SLEEP_SAT | 0,016 | 0,053 | 0,009 | 0,010 | 0,228 | 0,000 | 0,000 | 0,000 | 0,076 | 0,244 | 0,275 | 0,059 | 0,505 | 0,650 | 0,572 | 0,005 | 0,745 | 0,699 | 0,939 | 0,000 | 1,000 |

|  | Mantel's | |
| --- | --- | --- |
| **HIGH** | r | p |
| NSW_CUM | 0,241 | 0,001 |
| NSW_YRS | 0,268 | 0,001 |
| AGE | 0,047 | 0,165 |
| MEDI_LITE | -0,035 | 0,724 |
| IPAQ | 0,716 | 0,001 |
| PSQI | 0,092 | 0,045 |
| WHO | 0,008 | 0,417 |
| WAI | 0,143 | 0,011 |
| BMI | -0,038 | 0,749 |
| WHTR | -0,067 | 0,936 |
| METSIR | -0,045 | 0,765 |
| TYG | -0,021 | 0,637 |
| WYG | -0,004 | 0,468 |
| TC/HDL | -0,061 | 0,864 |
| LDL/HDL | -0,067 | 0,920 |
| IRCV | -0,076 | 0,910 |
| SMOKE | 0,033 | 0,127 |
| ALCOHOL | -0,009 | 0,533 |
| PHYS_ACT | 0,058 | 0,028 |
| SLEEP_HR | 0,018 | 0,339 |
| SLEEP_SAT | 0,032 | 0,209 |

|  | rValue | | | | | | | | | | | | | | | | | | | | |
| --- | --- | --- | --- | --- | --- | --- | --- | --- | --- | --- | --- | --- | --- | --- | --- | --- | --- | --- | --- | --- | --- |
| **SHORT** | NSW_CUM | NSW_YRS | AGE | MEDI_LITE | IPAQ | PSQI | WHO | WAI | BMI | WHTR | METSIR | TYG | WYG | TC/HDL | LDL/HDL | IRCV | SMOKE | ALCOHOL | PHYS_ACT | SLEEP_HR | SLEEP_SAT |
| NSW_CUM | 1,000 | 0,896 | 0,317 | 0,044 | -0,119 | 0,093 | -0,037 | -0,048 | 0,091 | 0,091 | 0,113 | 0,139 | 0,108 | 0,059 | 0,103 | 0,067 | 0,100 | -0,140 | -0,229 | -0,017 | 0,141 |
| NSW_YRS | 0,896 | 1,000 | 0,265 | 0,015 | -0,116 | -0,024 | -0,021 | 0,023 | 0,049 | 0,067 | 0,108 | 0,102 | 0,131 | 0,064 | 0,099 | 0,040 | 0,180 | -0,026 | -0,161 | 0,013 | 0,102 |
| AGE | 0,317 | 0,265 | 1,000 | -0,052 | -0,044 | 0,224 | -0,117 | -0,263 | 0,206 | 0,231 | 0,230 | 0,187 | 0,185 | 0,197 | 0,205 | 0,799 | 0,004 | -0,207 | -0,357 | -0,221 | 0,278 |
| MEDI_LITE | 0,044 | 0,015 | -0,052 | 1,000 | 0,061 | 0,078 | -0,060 | -0,201 | -0,103 | -0,076 | 0,000 | 0,069 | 0,066 | 0,001 | -0,016 | -0,116 | -0,101 | -0,050 | 0,148 | -0,275 | 0,093 |
| IPAQ | -0,119 | -0,116 | -0,044 | 0,061 | 1,000 | 0,098 | -0,046 | -0,184 | -0,064 | -0,006 | -0,020 | -0,207 | -0,055 | 0,040 | 0,073 | 0,093 | 0,167 | 0,115 | 0,111 | 0,036 | -0,009 |
| PSQI | 0,093 | -0,024 | 0,224 | 0,078 | 0,098 | 1,000 | -0,382 | -0,494 | -0,014 | -0,001 | -0,078 | -0,111 | -0,008 | 0,000 | 0,141 | 0,311 | -0,047 | 0,007 | 0,010 | -0,473 | 0,679 |
| WHO | -0,037 | -0,021 | -0,117 | -0,060 | -0,046 | -0,382 | 1,000 | 0,462 | 0,048 | 0,022 | 0,099 | -0,080 | -0,023 | 0,049 | -0,054 | -0,220 | 0,072 | -0,206 | 0,020 | 0,209 | -0,377 |
| WAI | -0,048 | 0,023 | -0,263 | -0,201 | -0,184 | -0,494 | 0,462 | 1,000 | 0,032 | -0,006 | 0,018 | 0,093 | 0,042 | 0,053 | -0,080 | -0,391 | -0,002 | -0,102 | 0,014 | 0,314 | -0,356 |
| BMI | 0,091 | 0,049 | 0,206 | -0,103 | -0,064 | -0,014 | 0,048 | 0,032 | 1,000 | 0,936 | 0,932 | 0,272 | 0,633 | 0,322 | 0,376 | 0,145 | -0,201 | -0,168 | -0,263 | 0,073 | 0,071 |
| WHTR | 0,091 | 0,067 | 0,231 | -0,076 | -0,006 | -0,001 | 0,022 | -0,006 | 0,936 | 1,000 | 0,869 | 0,235 | 0,570 | 0,301 | 0,370 | 0,093 | -0,224 | -0,198 | -0,225 | 0,059 | 0,062 |
| METSIR | 0,113 | 0,108 | 0,230 | 0,000 | -0,020 | -0,078 | 0,099 | 0,018 | 0,932 | 0,869 | 1,000 | 0,417 | 0,689 | 0,547 | 0,519 | 0,242 | -0,145 | -0,119 | -0,261 | -0,006 | 0,137 |
| TYG | 0,139 | 0,102 | 0,187 | 0,069 | -0,207 | -0,111 | -0,080 | 0,093 | 0,272 | 0,235 | 0,417 | 1,000 | 0,377 | 0,329 | 0,246 | 0,220 | -0,165 | -0,090 | -0,235 | -0,105 | 0,110 |
| WYG | 0,108 | 0,131 | 0,185 | 0,066 | -0,055 | -0,008 | -0,023 | 0,042 | 0,633 | 0,570 | 0,689 | 0,377 | 1,000 | 0,301 | 0,360 | 0,289 | -0,079 | -0,081 | -0,088 | -0,068 | 0,148 |
| TC/HDL | 0,059 | 0,064 | 0,197 | 0,001 | 0,040 | 0,000 | 0,049 | 0,053 | 0,322 | 0,301 | 0,547 | 0,329 | 0,301 | 1,000 | 0,951 | 0,273 | -0,091 | 0,002 | -0,232 | 0,037 | 0,127 |
| LDL/HDL | 0,103 | 0,099 | 0,205 | -0,016 | 0,073 | 0,141 | -0,054 | -0,080 | 0,376 | 0,370 | 0,519 | 0,246 | 0,360 | 0,951 | 1,000 | 0,295 | -0,022 | -0,030 | -0,226 | 0,000 | 0,180 |
| IRCV | 0,067 | 0,040 | 0,799 | -0,116 | 0,093 | 0,311 | -0,220 | -0,391 | 0,145 | 0,093 | 0,242 | 0,220 | 0,289 | 0,273 | 0,295 | 1,000 | 0,283 | -0,018 | -0,261 | -0,299 | 0,447 |
| SMOKE | 0,100 | 0,180 | 0,004 | -0,101 | 0,167 | -0,047 | 0,072 | -0,002 | -0,201 | -0,224 | -0,145 | -0,165 | -0,079 | -0,091 | -0,022 | 0,283 | 1,000 | 0,160 | -0,002 | 0,015 | 0,009 |
| ALCOHOL | -0,140 | -0,026 | -0,207 | -0,050 | 0,115 | 0,007 | -0,206 | -0,102 | -0,168 | -0,198 | -0,119 | -0,090 | -0,081 | 0,002 | -0,030 | -0,018 | 0,160 | 1,000 | 0,275 | 0,302 | -0,124 |
| PHYS_ACT | -0,229 | -0,161 | -0,357 | 0,148 | 0,111 | 0,010 | 0,020 | 0,014 | -0,263 | -0,225 | -0,261 | -0,235 | -0,088 | -0,232 | -0,226 | -0,261 | -0,002 | 0,275 | 1,000 | 0,079 | -0,030 |
| SLEEP_HR | -0,017 | 0,013 | -0,221 | -0,275 | 0,036 | -0,473 | 0,209 | 0,314 | 0,073 | 0,059 | -0,006 | -0,105 | -0,068 | 0,037 | 0,000 | -0,299 | 0,015 | 0,302 | 0,079 | 1,000 | -0,525 |
| SLEEP_SAT | 0,141 | 0,102 | 0,278 | 0,093 | -0,009 | 0,679 | -0,377 | -0,356 | 0,071 | 0,062 | 0,137 | 0,110 | 0,148 | 0,127 | 0,180 | 0,447 | 0,009 | -0,124 | -0,030 | -0,525 | 1,000 |

|  | pValue | | | | | | | | | | | | | | | | | | | | |
| --- | --- | --- | --- | --- | --- | --- | --- | --- | --- | --- | --- | --- | --- | --- | --- | --- | --- | --- | --- | --- | --- |
| **SHORT** | NSW_CUM | NSW_YRS | AGE | MEDI_LITE | IPAQ | PSQI | WHO | WAI | BMI | WHTR | METSIR | TYG | WYG | TC/HDL | LDL/HDL | IRCV | SMOKE | ALCOHOL | PHYS_ACT | SLEEP_HR | SLEEP_SAT |
| NSW_CUM | 1,000 | 0,000 | 0,003 | 0,690 | 0,284 | 0,402 | 0,739 | 0,666 | 0,415 | 0,414 | 0,379 | 0,278 | 0,332 | 0,645 | 0,436 | 0,599 | 0,367 | 0,208 | 0,037 | 0,877 | 0,203 |
| NSW_YRS | 0,000 | 1,000 | 0,015 | 0,896 | 0,292 | 0,831 | 0,848 | 0,837 | 0,658 | 0,548 | 0,402 | 0,426 | 0,236 | 0,615 | 0,454 | 0,751 | 0,102 | 0,815 | 0,142 | 0,905 | 0,357 |
| AGE | 0,003 | 0,015 | 1,000 | 0,641 | 0,690 | 0,041 | 0,291 | 0,016 | 0,060 | 0,035 | 0,070 | 0,142 | 0,092 | 0,118 | 0,119 | 0,000 | 0,968 | 0,059 | 0,001 | 0,044 | 0,010 |
| MEDI_LITE | 0,690 | 0,896 | 0,641 | 1,000 | 0,581 | 0,481 | 0,587 | 0,067 | 0,349 | 0,493 | 0,998 | 0,591 | 0,552 | 0,991 | 0,901 | 0,360 | 0,362 | 0,651 | 0,178 | 0,011 | 0,399 |
| IPAQ | 0,284 | 0,292 | 0,690 | 0,581 | 1,000 | 0,377 | 0,676 | 0,094 | 0,564 | 0,956 | 0,877 | 0,104 | 0,617 | 0,756 | 0,584 | 0,465 | 0,129 | 0,297 | 0,314 | 0,743 | 0,934 |
| PSQI | 0,402 | 0,831 | 0,041 | 0,481 | 0,377 | 1,000 | 0,000 | 0,000 | 0,903 | 0,995 | 0,542 | 0,388 | 0,940 | 1,000 | 0,285 | 0,012 | 0,671 | 0,949 | 0,928 | 0,000 | 0,000 |
| WHO | 0,739 | 0,848 | 0,291 | 0,587 | 0,676 | 0,000 | 1,000 | 0,000 | 0,663 | 0,843 | 0,439 | 0,534 | 0,838 | 0,703 | 0,686 | 0,081 | 0,513 | 0,061 | 0,858 | 0,056 | 0,000 |
| WAI | 0,666 | 0,837 | 0,016 | 0,067 | 0,094 | 0,000 | 0,000 | 1,000 | 0,770 | 0,955 | 0,889 | 0,467 | 0,705 | 0,680 | 0,549 | 0,001 | 0,988 | 0,357 | 0,901 | 0,004 | 0,001 |
| BMI | 0,415 | 0,658 | 0,060 | 0,349 | 0,564 | 0,903 | 0,663 | 0,770 | 1,000 | 0,000 | 0,000 | 0,031 | 0,000 | 0,009 | 0,003 | 0,252 | 0,067 | 0,127 | 0,016 | 0,511 | 0,522 |
| WHTR | 0,414 | 0,548 | 0,035 | 0,493 | 0,956 | 0,995 | 0,843 | 0,955 | 0,000 | 1,000 | 0,000 | 0,064 | 0,000 | 0,016 | 0,004 | 0,466 | 0,041 | 0,071 | 0,040 | 0,597 | 0,576 |
| METSIR | 0,379 | 0,402 | 0,070 | 0,998 | 0,877 | 0,542 | 0,439 | 0,889 | 0,000 | 0,000 | 1,000 | 0,001 | 0,000 | 0,000 | 0,000 | 0,056 | 0,258 | 0,353 | 0,039 | 0,964 | 0,285 |
| TYG | 0,278 | 0,426 | 0,142 | 0,591 | 0,104 | 0,388 | 0,534 | 0,467 | 0,031 | 0,064 | 0,001 | 1,000 | 0,002 | 0,008 | 0,061 | 0,083 | 0,196 | 0,482 | 0,063 | 0,412 | 0,393 |
| WYG | 0,332 | 0,236 | 0,092 | 0,552 | 0,617 | 0,940 | 0,838 | 0,705 | 0,000 | 0,000 | 0,000 | 0,002 | 1,000 | 0,015 | 0,005 | 0,021 | 0,473 | 0,464 | 0,425 | 0,537 | 0,178 |
| TC/HDL | 0,645 | 0,615 | 0,118 | 0,991 | 0,756 | 1,000 | 0,703 | 0,680 | 0,009 | 0,016 | 0,000 | 0,008 | 0,015 | 1,000 | 0,000 | 0,029 | 0,474 | 0,988 | 0,065 | 0,773 | 0,317 |
| LDL/HDL | 0,436 | 0,454 | 0,119 | 0,901 | 0,584 | 0,285 | 0,686 | 0,549 | 0,003 | 0,004 | 0,000 | 0,061 | 0,005 | 0,000 | 1,000 | 0,023 | 0,871 | 0,819 | 0,086 | 0,999 | 0,172 |
| IRCV | 0,599 | 0,751 | 0,000 | 0,360 | 0,465 | 0,012 | 0,081 | 0,001 | 0,252 | 0,466 | 0,056 | 0,083 | 0,021 | 0,029 | 0,023 | 1,000 | 0,024 | 0,890 | 0,037 | 0,017 | 0,000 |
| SMOKE | 0,367 | 0,102 | 0,968 | 0,362 | 0,129 | 0,671 | 0,513 | 0,988 | 0,067 | 0,041 | 0,258 | 0,196 | 0,473 | 0,474 | 0,871 | 0,024 | 1,000 | 0,146 | 0,988 | 0,896 | 0,939 |
| ALCOHOL | 0,208 | 0,815 | 0,059 | 0,651 | 0,297 | 0,949 | 0,061 | 0,357 | 0,127 | 0,071 | 0,353 | 0,482 | 0,464 | 0,988 | 0,819 | 0,890 | 0,146 | 1,000 | 0,011 | 0,005 | 0,260 |
| PHYS_ACT | 0,037 | 0,142 | 0,001 | 0,178 | 0,314 | 0,928 | 0,858 | 0,901 | 0,016 | 0,040 | 0,039 | 0,063 | 0,425 | 0,065 | 0,086 | 0,037 | 0,988 | 0,011 | 1,000 | 0,475 | 0,783 |
| SLEEP_HR | 0,877 | 0,905 | 0,044 | 0,011 | 0,743 | 0,000 | 0,056 | 0,004 | 0,511 | 0,597 | 0,964 | 0,412 | 0,537 | 0,773 | 0,999 | 0,017 | 0,896 | 0,005 | 0,475 | 1,000 | 0,000 |
| SLEEP_SAT | 0,203 | 0,357 | 0,010 | 0,399 | 0,934 | 0,000 | 0,000 | 0,001 | 0,522 | 0,576 | 0,285 | 0,393 | 0,178 | 0,317 | 0,172 | 0,000 | 0,939 | 0,260 | 0,783 | 0,000 | 1,000 |

|  | Mantel's | |
| --- | --- | --- |
| **SHORT** | r | p |
| NSW_CUM | 0,077 | 0,154 |
| NSW_YRS | 0,039 | 0,245 |
| AGE | 0,070 | 0,138 |
| MEDI_LITE | -0,008 | 0,455 |
| IPAQ | 1,000 | 0,001 |
| PSQI | 0,106 | 0,122 |
| WHO | 0,149 | 0,053 |
| WAI | 0,265 | 0,016 |
| BMI | -0,061 | 0,733 |
| WHTR | -0,001 | 0,395 |
| METSIR | -0,091 | 0,885 |
| TYG | -0,077 | 0,857 |
| WYG | -0,067 | 0,777 |
| TC/HDL | 0,056 | 0,210 |
| LDL/HDL | 0,082 | 0,163 |
| IRCV | 0,095 | 0,123 |
| SMOKE | 0,166 | 0,042 |
| ALCOHOL | 0,102 | 0,135 |
| PHYS_ACT | -0,025 | 0,766 |
| SLEEP_HR | 0,055 | 0,222 |
| SLEEP_SAT | 0,116 | 0,111 |

|  | rValue | | | | | | | | | | | | | | | | | | | | |
| --- | --- | --- | --- | --- | --- | --- | --- | --- | --- | --- | --- | --- | --- | --- | --- | --- | --- | --- | --- | --- | --- |
| **LONG** | NSW_CUM | NSW_YRS | AGE | MEDI_LITE | IPAQ | PSQI | WHO | WAI | BMI | WHTR | METSIR | TYG | WYG | TC/HDL | LDL/HDL | IRCV | SMOKE | ALCOHOL | PHYS_ACT | SLEEP_HR | SLEEP_SAT |
| NSW_CUM | 1,000 | 0,791 | 0,453 | 0,022 | -0,002 | 0,327 | -0,291 | -0,384 | 0,131 | 0,124 | 0,154 | 0,163 | 0,229 | 0,118 | 0,194 | 0,323 | -0,108 | 0,203 | -0,075 | -0,294 | 0,179 |
| NSW_YRS | 0,791 | 1,000 | 0,503 | -0,082 | 0,152 | 0,300 | -0,310 | -0,410 | 0,083 | 0,067 | 0,052 | 0,074 | 0,147 | -0,064 | 0,009 | 0,275 | -0,119 | 0,135 | -0,112 | -0,205 | 0,118 |
| AGE | 0,453 | 0,503 | 1,000 | 0,091 | -0,023 | 0,355 | -0,234 | -0,313 | 0,129 | 0,138 | 0,135 | 0,326 | 0,271 | 0,092 | 0,224 | 0,667 | -0,297 | 0,095 | 0,004 | -0,398 | 0,206 |
| MEDI_LITE | 0,022 | -0,082 | 0,091 | 1,000 | -0,027 | 0,054 | 0,273 | 0,081 | -0,116 | -0,120 | -0,079 | -0,058 | -0,136 | 0,022 | 0,078 | 0,064 | -0,129 | 0,088 | 0,138 | -0,051 | 0,236 |
| IPAQ | -0,002 | 0,152 | -0,023 | -0,027 | 1,000 | -0,062 | 0,172 | 0,035 | -0,214 | -0,223 | -0,218 | -0,248 | -0,300 | -0,305 | -0,246 | -0,104 | 0,202 | 0,300 | 0,399 | -0,101 | -0,268 |
| PSQI | 0,327 | 0,300 | 0,355 | 0,054 | -0,062 | 1,000 | -0,525 | -0,613 | 0,016 | 0,062 | 0,043 | 0,066 | 0,123 | 0,066 | 0,141 | 0,319 | -0,033 | 0,173 | -0,089 | -0,580 | 0,572 |
| WHO | -0,291 | -0,310 | -0,234 | 0,273 | 0,172 | -0,525 | 1,000 | 0,648 | -0,080 | -0,138 | -0,107 | -0,133 | -0,158 | -0,121 | -0,258 | -0,313 | -0,025 | -0,083 | 0,249 | 0,263 | -0,407 |
| WAI | -0,384 | -0,410 | -0,313 | 0,081 | 0,035 | -0,613 | 0,648 | 1,000 | -0,099 | -0,140 | -0,103 | -0,116 | -0,102 | 0,009 | -0,105 | -0,332 | -0,138 | -0,121 | 0,116 | 0,306 | -0,463 |
| BMI | 0,131 | 0,083 | 0,129 | -0,116 | -0,214 | 0,016 | -0,080 | -0,099 | 1,000 | 0,933 | 0,942 | 0,394 | 0,702 | 0,420 | 0,410 | 0,241 | -0,210 | -0,155 | -0,193 | -0,219 | -0,198 |
| WHTR | 0,124 | 0,067 | 0,138 | -0,120 | -0,223 | 0,062 | -0,138 | -0,140 | 0,933 | 1,000 | 0,887 | 0,402 | 0,719 | 0,443 | 0,435 | 0,338 | -0,152 | -0,171 | -0,198 | -0,268 | -0,111 |
| METSIR | 0,154 | 0,052 | 0,135 | -0,079 | -0,218 | 0,043 | -0,107 | -0,103 | 0,942 | 0,887 | 1,000 | 0,545 | 0,736 | 0,625 | 0,599 | 0,364 | -0,146 | -0,181 | -0,172 | -0,178 | -0,149 |
| TYG | 0,163 | 0,074 | 0,326 | -0,058 | -0,248 | 0,066 | -0,133 | -0,116 | 0,394 | 0,402 | 0,545 | 1,000 | 0,608 | 0,555 | 0,460 | 0,452 | -0,176 | -0,144 | -0,102 | -0,099 | 0,096 |
| WYG | 0,229 | 0,147 | 0,271 | -0,136 | -0,300 | 0,123 | -0,158 | -0,102 | 0,702 | 0,719 | 0,736 | 0,608 | 1,000 | 0,415 | 0,352 | 0,264 | -0,288 | -0,124 | -0,326 | -0,327 | 0,029 |
| TC/HDL | 0,118 | -0,064 | 0,092 | 0,022 | -0,305 | 0,066 | -0,121 | 0,009 | 0,420 | 0,443 | 0,625 | 0,555 | 0,415 | 1,000 | 0,978 | 0,493 | 0,060 | -0,204 | -0,098 | -0,004 | 0,066 |
| LDL/HDL | 0,194 | 0,009 | 0,224 | 0,078 | -0,246 | 0,141 | -0,258 | -0,105 | 0,410 | 0,435 | 0,599 | 0,460 | 0,352 | 0,978 | 1,000 | 0,545 | 0,080 | -0,130 | -0,139 | -0,122 | 0,113 |
| IRCV | 0,323 | 0,275 | 0,667 | 0,064 | -0,104 | 0,319 | -0,313 | -0,332 | 0,241 | 0,338 | 0,364 | 0,452 | 0,264 | 0,493 | 0,545 | 1,000 | 0,179 | 0,010 | -0,045 | -0,303 | 0,234 |
| SMOKE | -0,108 | -0,119 | -0,297 | -0,129 | 0,202 | -0,033 | -0,025 | -0,138 | -0,210 | -0,152 | -0,146 | -0,176 | -0,288 | 0,060 | 0,080 | 0,179 | 1,000 | 0,109 | -0,139 | 0,099 | -0,013 |
| ALCOHOL | 0,203 | 0,135 | 0,095 | 0,088 | 0,300 | 0,173 | -0,083 | -0,121 | -0,155 | -0,171 | -0,181 | -0,144 | -0,124 | -0,204 | -0,130 | 0,010 | 0,109 | 1,000 | 0,127 | -0,147 | 0,077 |
| PHYS_ACT | -0,075 | -0,112 | 0,004 | 0,138 | 0,399 | -0,089 | 0,249 | 0,116 | -0,193 | -0,198 | -0,172 | -0,102 | -0,326 | -0,098 | -0,139 | -0,045 | -0,139 | 0,127 | 1,000 | 0,020 | -0,093 |
| SLEEP_HR | -0,294 | -0,205 | -0,398 | -0,051 | -0,101 | -0,580 | 0,263 | 0,306 | -0,219 | -0,268 | -0,178 | -0,099 | -0,327 | -0,004 | -0,122 | -0,303 | 0,099 | -0,147 | 0,020 | 1,000 | -0,457 |
| SLEEP_SAT | 0,179 | 0,118 | 0,206 | 0,236 | -0,268 | 0,572 | -0,407 | -0,463 | -0,198 | -0,111 | -0,149 | 0,096 | 0,029 | 0,066 | 0,113 | 0,234 | -0,013 | 0,077 | -0,093 | -0,457 | 1,000 |

|  | pValue | | | | | | | | | | | | | | | | | | | | |
| --- | --- | --- | --- | --- | --- | --- | --- | --- | --- | --- | --- | --- | --- | --- | --- | --- | --- | --- | --- | --- | --- |
| **LONG** | NSW_CUM | NSW_YRS | AGE | MEDI_LITE | IPAQ | PSQI | WHO | WAI | BMI | WHTR | METSIR | TYG | WYG | TC/HDL | LDL/HDL | IRCV | SMOKE | ALCOHOL | PHYS_ACT | SLEEP_HR | SLEEP_SAT |
| NSW_CUM | 1,000 | 0,000 | 0,000 | 0,864 | 0,987 | 0,009 | 0,021 | 0,002 | 0,305 | 0,333 | 0,235 | 0,209 | 0,074 | 0,363 | 0,156 | 0,012 | 0,399 | 0,111 | 0,559 | 0,020 | 0,161 |
| NSW_YRS | 0,000 | 1,000 | 0,000 | 0,522 | 0,236 | 0,017 | 0,013 | 0,001 | 0,517 | 0,599 | 0,693 | 0,573 | 0,254 | 0,622 | 0,946 | 0,033 | 0,352 | 0,292 | 0,383 | 0,106 | 0,359 |
| AGE | 0,000 | 0,000 | 1,000 | 0,480 | 0,857 | 0,004 | 0,065 | 0,012 | 0,314 | 0,279 | 0,300 | 0,010 | 0,033 | 0,481 | 0,100 | 0,000 | 0,018 | 0,460 | 0,978 | 0,001 | 0,106 |
| MEDI_LITE | 0,864 | 0,522 | 0,480 | 1,000 | 0,832 | 0,677 | 0,031 | 0,530 | 0,363 | 0,349 | 0,545 | 0,659 | 0,291 | 0,868 | 0,571 | 0,629 | 0,312 | 0,491 | 0,281 | 0,693 | 0,063 |
| IPAQ | 0,987 | 0,236 | 0,857 | 0,832 | 1,000 | 0,631 | 0,178 | 0,787 | 0,093 | 0,079 | 0,092 | 0,054 | 0,018 | 0,017 | 0,070 | 0,429 | 0,113 | 0,017 | 0,001 | 0,431 | 0,034 |
| PSQI | 0,009 | 0,017 | 0,004 | 0,677 | 0,631 | 1,000 | 0,000 | 0,000 | 0,898 | 0,627 | 0,740 | 0,616 | 0,339 | 0,612 | 0,304 | 0,013 | 0,799 | 0,174 | 0,488 | 0,000 | 0,000 |
| WHO | 0,021 | 0,013 | 0,065 | 0,031 | 0,178 | 0,000 | 1,000 | 0,000 | 0,533 | 0,281 | 0,413 | 0,306 | 0,220 | 0,354 | 0,057 | 0,015 | 0,846 | 0,517 | 0,049 | 0,037 | 0,001 |
| WAI | 0,002 | 0,001 | 0,012 | 0,530 | 0,787 | 0,000 | 0,000 | 1,000 | 0,440 | 0,275 | 0,429 | 0,372 | 0,430 | 0,942 | 0,445 | 0,010 | 0,282 | 0,346 | 0,364 | 0,015 | 0,000 |
| BMI | 0,305 | 0,517 | 0,314 | 0,363 | 0,093 | 0,898 | 0,533 | 0,440 | 1,000 | 0,000 | 0,000 | 0,002 | 0,000 | 0,001 | 0,002 | 0,064 | 0,098 | 0,224 | 0,130 | 0,084 | 0,119 |
| WHTR | 0,333 | 0,599 | 0,279 | 0,349 | 0,079 | 0,627 | 0,281 | 0,275 | 0,000 | 1,000 | 0,000 | 0,001 | 0,000 | 0,000 | 0,001 | 0,008 | 0,235 | 0,179 | 0,120 | 0,034 | 0,386 |
| METSIR | 0,235 | 0,693 | 0,300 | 0,545 | 0,092 | 0,740 | 0,413 | 0,429 | 0,000 | 0,000 | 1,000 | 0,000 | 0,000 | 0,000 | 0,000 | 0,004 | 0,263 | 0,164 | 0,184 | 0,170 | 0,253 |
| TYG | 0,209 | 0,573 | 0,010 | 0,659 | 0,054 | 0,616 | 0,306 | 0,372 | 0,002 | 0,001 | 0,000 | 1,000 | 0,000 | 0,000 | 0,000 | 0,000 | 0,175 | 0,268 | 0,433 | 0,448 | 0,462 |
| WYG | 0,074 | 0,254 | 0,033 | 0,291 | 0,018 | 0,339 | 0,220 | 0,430 | 0,000 | 0,000 | 0,000 | 0,000 | 1,000 | 0,001 | 0,008 | 0,042 | 0,023 | 0,337 | 0,010 | 0,010 | 0,821 |
| TC/HDL | 0,363 | 0,622 | 0,481 | 0,868 | 0,017 | 0,612 | 0,354 | 0,942 | 0,001 | 0,000 | 0,000 | 0,000 | 0,001 | 1,000 | 0,000 | 0,000 | 0,647 | 0,115 | 0,450 | 0,973 | 0,616 |
| LDL/HDL | 0,156 | 0,946 | 0,100 | 0,571 | 0,070 | 0,304 | 0,057 | 0,445 | 0,002 | 0,001 | 0,000 | 0,000 | 0,008 | 0,000 | 1,000 | 0,000 | 0,560 | 0,344 | 0,310 | 0,374 | 0,413 |
| IRCV | 0,012 | 0,033 | 0,000 | 0,629 | 0,429 | 0,013 | 0,015 | 0,010 | 0,064 | 0,008 | 0,004 | 0,000 | 0,042 | 0,000 | 0,000 | 1,000 | 0,172 | 0,940 | 0,733 | 0,018 | 0,072 |
| SMOKE | 0,399 | 0,352 | 0,018 | 0,312 | 0,113 | 0,799 | 0,846 | 0,282 | 0,098 | 0,235 | 0,263 | 0,175 | 0,023 | 0,647 | 0,560 | 0,172 | 1,000 | 0,394 | 0,277 | 0,439 | 0,920 |
| ALCOHOL | 0,111 | 0,292 | 0,460 | 0,491 | 0,017 | 0,174 | 0,517 | 0,346 | 0,224 | 0,179 | 0,164 | 0,268 | 0,337 | 0,115 | 0,344 | 0,940 | 0,394 | 1,000 | 0,320 | 0,249 | 0,546 |
| PHYS_ACT | 0,559 | 0,383 | 0,978 | 0,281 | 0,001 | 0,488 | 0,049 | 0,364 | 0,130 | 0,120 | 0,184 | 0,433 | 0,010 | 0,450 | 0,310 | 0,733 | 0,277 | 0,320 | 1,000 | 0,874 | 0,470 |
| SLEEP_HR | 0,020 | 0,106 | 0,001 | 0,693 | 0,431 | 0,000 | 0,037 | 0,015 | 0,084 | 0,034 | 0,170 | 0,448 | 0,010 | 0,973 | 0,374 | 0,018 | 0,439 | 0,249 | 0,874 | 1,000 | 0,000 |
| SLEEP_SAT | 0,161 | 0,359 | 0,106 | 0,063 | 0,034 | 0,000 | 0,001 | 0,000 | 0,119 | 0,386 | 0,253 | 0,462 | 0,821 | 0,616 | 0,413 | 0,072 | 0,920 | 0,546 | 0,470 | 0,000 | 1,000 |

|  | Mantel's | |
| --- | --- | --- |
| **LONG** | r | p |
| NSW_CUM | 0,182 | 0,009 |
| NSW_YRS | 0,184 | 0,003 |
| AGE | 0,038 | 0,275 |
| MEDI_LITE | -0,041 | 0,699 |
| IPAQ | 0,748 | 0,001 |
| PSQI | 0,014 | 0,378 |
| WHO | -0,031 | 0,660 |
| WAI | 0,054 | 0,224 |
| BMI | -0,030 | 0,634 |
| WHTR | -0,064 | 0,823 |
| METSIR | -0,023 | 0,528 |
| TYG | -0,013 | 0,495 |
| WYG | 0,024 | 0,329 |
| TC/HDL | -0,012 | 0,519 |
| LDL/HDL | -0,022 | 0,556 |
| IRCV | -0,082 | 0,865 |
| SMOKE | 0,021 | 0,202 |
| ALCOHOL | 0,007 | 0,291 |
| PHYS_ACT | 0,093 | 0,012 |
| SLEEP_HR | -0,012 | 0,529 |
| SLEEP_SAT | -0,028 | 0,668 |

Variable definitions: AGE, Age (years); NSW, Ever worked night shifts (0 = no, 1 = yes); NSW_CUM, Cumulative night-shift exposure (lifetime number of night shifts); NSW_YRS, Total years of night-shift work; MEDI_LITE, Mediterranean diet adherence score; IPAQ, International Physical Activity Questionnaire; PSQI, Pittsburgh Sleep Quality Index; WHO, WHO-5 well-being questionnaire; WAI, Work Ability Index; BMI, Body mass index (kg/m²); WHTR, Waist-to-height ratio; METSIR, Metabolic Score for Insulin Resistance; TYG, Triglycerides–glucose index; WYG, Waist–triglycerides–glucose index; TC/HDL, Total cholesterol / HDL cholesterol ratio; LDL/HDL, LDL cholesterol / HDL cholesterol ratio; IRCV, Cardiovascular Risk Index; SMOKE, Smoking status (0 = never, 1 = former, 2 = current); ALCOHOL, Alcohol consumption (0 = no, 1 = yes); PHYS_ACT, Regular physical activity at least once per week (0 = no, 1 = yes); SLEEP_HR, Average sleep duration over the past year (hours/night); SLEEP_SAT, Overall satisfaction with sleep quality (0 = very, 1 = fairly, 2 = little, 3 = not at all).

# References

1. Sofi F, Dinu M, Pagliai G, Marcucci R, Casini A. Validation of a literature-based adherence score to Mediterranean diet: the MEDI-LITE score. *Int J Food Sci Nutr* (2017) 68:757–762. doi: 10.1080/09637486.2017.1287884

2. Hagströmer M, Oja P, Sjöström M. The International Physical Activity Questionnaire (IPAQ): a study of concurrent and construct validity. *Public Health Nutr* (2006) 9:755–762. doi: 10.1079/PHN2005898

3. Buysse DJ, Reynolds CF, Monk TH, Berman SR, Kupfer DJ. The Pittsburgh sleep quality index: A new instrument for psychiatric practice and research. *Psychiatry Res* (1989) 28:193–213. doi: 10.1016/0165-1781(89)90047-4

4. Topp CW, Østergaard SD, Søndergaard S, Bech P. The WHO-5 Well-Being Index: A Systematic Review of the Literature. *Psychother Psychosom* (2015) 84:167–176. doi: 10.1159/000376585

5. Ilmarinen J. The Work Ability Index (WAI). *Occup Med (Chic Ill)* (2006) 57:160–160. doi: 10.1093/occmed/kqm008

6. Khanna D, Peltzer C, Kahar P, Parmar MS. Body Mass Index (BMI): A Screening Tool Analysis. *Cureus* (2022) doi: 10.7759/cureus.22119

7. Lu Y, Liu S, Qiao Y, Li G, Wu Y, Ke C. Waist-to-height ratio, waist circumference, body mass index, waist divided by height0.5 and the risk of cardiometabolic multimorbidity: A national longitudinal cohort study. *Nutr Metab Cardiovasc Dis* (2021) 31:2644–2651. doi: 10.1016/j.numecd.2021.05.026

8. Duan M, Zhao X, Li S, Miao G, Bai L, Zhang Q, Yang W, Zhao X. Metabolic score for insulin resistance (METS-IR) predicts all-cause and cardiovascular mortality in the general population: evidence from NHANES 2001–2018. *Cardiovasc Diabetol* (2024) 23:243. doi: 10.1186/s12933-024-02334-8

9. Avagimyan A, Pogosova N, Fogacci F, Aghajanova E, Djndoyan Z, Patoulias D, Sasso L Lo, Bernardi M, Faggiano A, Mohammadifard N, et al. Triglyceride-glucose index (TyG) as a novel biomarker in the era of cardiometabolic medicine. *Int J Cardiol* (2025) 418:132663. doi: 10.1016/j.ijcard.2024.132663

10. Zhao X, Song B, Yao T, Fan H, Liu T, Gao G, Wang K, Lu W, Liu C. Waist circumference glucose, a novel and effective predictor of type 2 diabetes: a prospective cohort study. *Front Endocrinol (Lausanne)* (2024) 15: doi: 10.3389/fendo.2024.1427785

11. Jacobs DR, Mebane IL, Bangdiwala SI, Criqui MH, Tyroler HA. High density lipoprotein cholesterol as a predictor of cardiovascular disease mortality in men and women: the follow-up study of the lipid research clinics prevalence study. *Am J Epidemiol* (1990) 131:32–47. doi: 10.1093/oxfordjournals.aje.a115483

12. Pende A, Grondona C, Bertolini S. Correlation between Progetto Cuore risk score and early cardiovascular damage in never treated subjects. *Cardiovasc Ultrasound* (2008) 6:47. doi: 10.1186/1476-7120-6-47

13. Palmieri L, Donfrancesco C, Giampaoli S, Trojani M, Panico S, Vanuzzo D, Pilottoc L, Cesana G, Ferrario M, Chiodini P, et al. Favorable cardiovascular risk profile and 10-year coronary heart disease incidence in women and men: results from the Progetto CUORE. *Eur J Cardiovasc Prev Rehabil* (2006) 13:562–570. doi: 10.1097/01.hjr.0000221866.27039.4b
